# *Pseudomonas aeruginosa* PA1006, Which Plays a Role in Molybdenum Homeostasis, Is Required for Nitrate Utilization, Biofilm Formation, and Virulence

**DOI:** 10.1371/journal.pone.0055594

**Published:** 2013-02-08

**Authors:** Melanie J. Filiatrault, Gregory Tombline, Victoria E. Wagner, Nadine Van Alst, Kendra Rumbaugh, Pam Sokol, Johanna Schwingel, Barbara H. Iglewski

**Affiliations:** 1 Department of Microbiology and Immunology, University of Rochester School of Medicine and Dentistry, Rochester, New York, United States of America; 2 Department of Surgery, Texas Tech University Health Sciences Center, Lubbock, Texas, United States of America; 3 Department of Microbiology and Infectious Diseases, University of Calgary, Calgary, Alberta, Canada; Centre National de la Recherche Scientifique, Aix-Marseille Université, France

## Abstract

*Pseudomonas aeruginosa (Pae)* is a clinically important opportunistic pathogen. Herein, we demonstrate that the PA1006 protein is critical for all nitrate reductase activities, growth as a biofilm in a continuous flow system, as well as virulence in mouse burn and rat lung model systems. Microarray analysis revealed that Δ*PA1006* cells displayed extensive alterations in gene expression including nitrate-responsive, quorum sensing (including PQS production), and iron-regulated genes, as well as molybdenum cofactor and Fe-S cluster biosynthesis factors, members of the TCA cycle, and Type VI Secretion System components. Phenotype Microarray™ profiles of Δ*PA1006* aerobic cultures using Biolog plates also revealed a reduced ability to utilize a number of TCA cycle intermediates as well as a failure to utilize xanthine as a sole source of nitrogen. As a whole, these data indicate that the loss of *PA1006* confers extensive changes in *Pae* metabolism. Based upon homology of PA1006 to the *E. coli* YhhP protein and data from the accompanying study, loss of PA1006 persulfuration and/or molybdenum homeostasis are likely the cause of extensive metabolic alterations that impact biofilm development and virulence in the Δ*PA1006* mutant.

## Introduction

*P. aeruginosa* (*Pae*) is a ubiquitous opportunistic nosocomial pathogen that infects individuals with pre-disposing conditions such as cancer, AIDS, burns, and importantly, Cystic Fibrosis (CF). *Pae* is highly clinically relevant since it causes ∼10% of the 2 million life-threatening nosocomial infections that occur annually in the United States [1]. *Pae* possesses numerous virulence factors that contribute to pathogenesis including proteases, exotoxin A, hydrogen cyanide, and phenazines [2], [3]. In addition, despite intensive treatment, eradication of *Pae* is extremely difficult due to its intrinsic ability to resist a variety of antimicrobial agents [4], [5].

Mounting evidence indicates that *Pae* experiences microaerobic as well as anaerobic environments during biofilm development and during infection *in vivo* [6], [7], [8], [9], [10], [11], [12], [13], [14]. In biofilms, cell density and secreted factors may contribute to oxygen depletion/availability. During infection, a mucoidy phenotype, resulting from the expression of the polysaccharide alginate, often develops. It has been speculated that this dense covering provides a barrier to oxygen diffusion. In the CF lung, a failure in the Cl channel CFTR (Cystic Fibrosis transmembrane conductance regulator) produces salt imbalances that allow thick mucus to develop. Excess mucus hinders the beating of epithelial cilia likely allowing bacteria to settle. *Pae* appears well-suited for this niche. Experiments measuring the effect of prepared mucus on *Pae* biofilms *in vitro* suggest that the dense mucus prevalent in a CF lung may exert a positive effect by slowing swimming, increasing the local concentration of autoinducers, and restricting access of host factors such as lactoferrin [15]. Reduced oxygen tension does not appear to be a problem in infection, since biofilms can form under anaerobic conditions [8]. Compounding matters, biofilms and anaerobic growth also appear to contribute to increased antibiotic resistance [6]. Proteins that enable biofilm development and anaerobic metabolism *in vivo* (in the host) are not well defined and likely also contribute to virulence potential.

In the absence of oxygen, *Pae* may respire by utilizing nitrate or nitrite as alternative terminal electron acceptors via denitrification [16]. Denitrification enzymes reduce nitrate (NO_3_−) to nitrite (NO_2_−), and subsequently to nitric oxide (NO), nitrous oxide (N_2_O), and finally, dinitrogen gas (N_2_). In addition to denitrification enzymes *per se*, nitrate alters the expression of a number of genes involved in virulence factor production [17]. Consistent with a correlation between virulence, biofilms, and anaerobic growth by denitrification, NarGHI (the membrane associated nitrate reductase) is required for growth in the CF lung [18]. NarGHI is also required for biofilm formation in the flow system as well as for virulence in the *C. elegans* model of infection [19]. It is also notable that NO elicits biofilm dispersal suggesting that denitrification intermediates may also serve as signals and their accumulation must be carefully modulated by *Pae* [20]. Considering these promising initial results, further investigations into the relationship between anaerobic growth, denitrification (and associated signaling pathways), biofilm formation, and virulence are likely to yield novel drug targets.

A number of novel genes involved in anaerobic growth of *Pae* have been recently identified by transposon (Tn) mutagenesis [21]. A Tn insertion in one gene, PA1006, resulted in an inability to grow anaerobically using nitrate as the terminal electron acceptor but did not affect the ability of *Pae* to grow anaerobically with nitrite or arginine. This suggested that PA1006 encodes for a protein that plays a role in nitrate utilization. The current annotation for PA1006 (GI:15596203) indicates that it is SirA-like or related to the *E. coli* YhhP/TusA protein and that it may function as a mediator of disulfide bonds. SirA is Two-Component System transcriptional regulator that responds to environmental cues via phosphorylation-mediated signals arising from sensor kinases [22]. PA1006 is not the *Pae* SirA equivalent. Alignment of their sequences shows that PA1006 is only ∼10% homologous to SirA (data not shown). In fact, it is already known that PA2586/GacA (GI:15597782) is the *Pae* equivalent of *E. coli* SirA/UvrY/GacA (GI:16129861). *E. coli* YhhP/TusA is a persulfide-sulfur trafficking protein that is required to make 2′-thiouridine present in certain tRNAs [23]. PA1006 is also not the functional homolog of *E. coli* YhhP/TusA. Rather, PA1564 appears to be the equivalent (see alignment in Figure 1 of [24]). *E. coli* YhhP/TusA mutants are barely viable, showing a severe growth arrest phenotype due to filamentation [24]. In contrast, PA1006 deletion mutants do not display filamentation. YhhP/TusA is modified in the form of a persulfide on a highly conserved Cys residue that is required for its activity. It is likely due to this conserved Cys that these proteins are generally classified as mediators of disulfide bonds. However, there is currently no evidence to support the idea that YhhP/TusA or other persulfide-modified proteins function as general mediators of disulfide bonds analogous to protein disulfide isomerases or glutathione. Rather, Yhhp/TusA-like proteins appear to mediate very specific transfer of sulfur via a transient persulfide group. For example, in the case of YhhP/TusA, the sulfur transfers specifically to TusBCD [23]. Therefore, when the studies presented herein were initiated, the biological/functional role of PA1006 could not be easily predicted and was not expected to be generalized.

**Figure 1:**
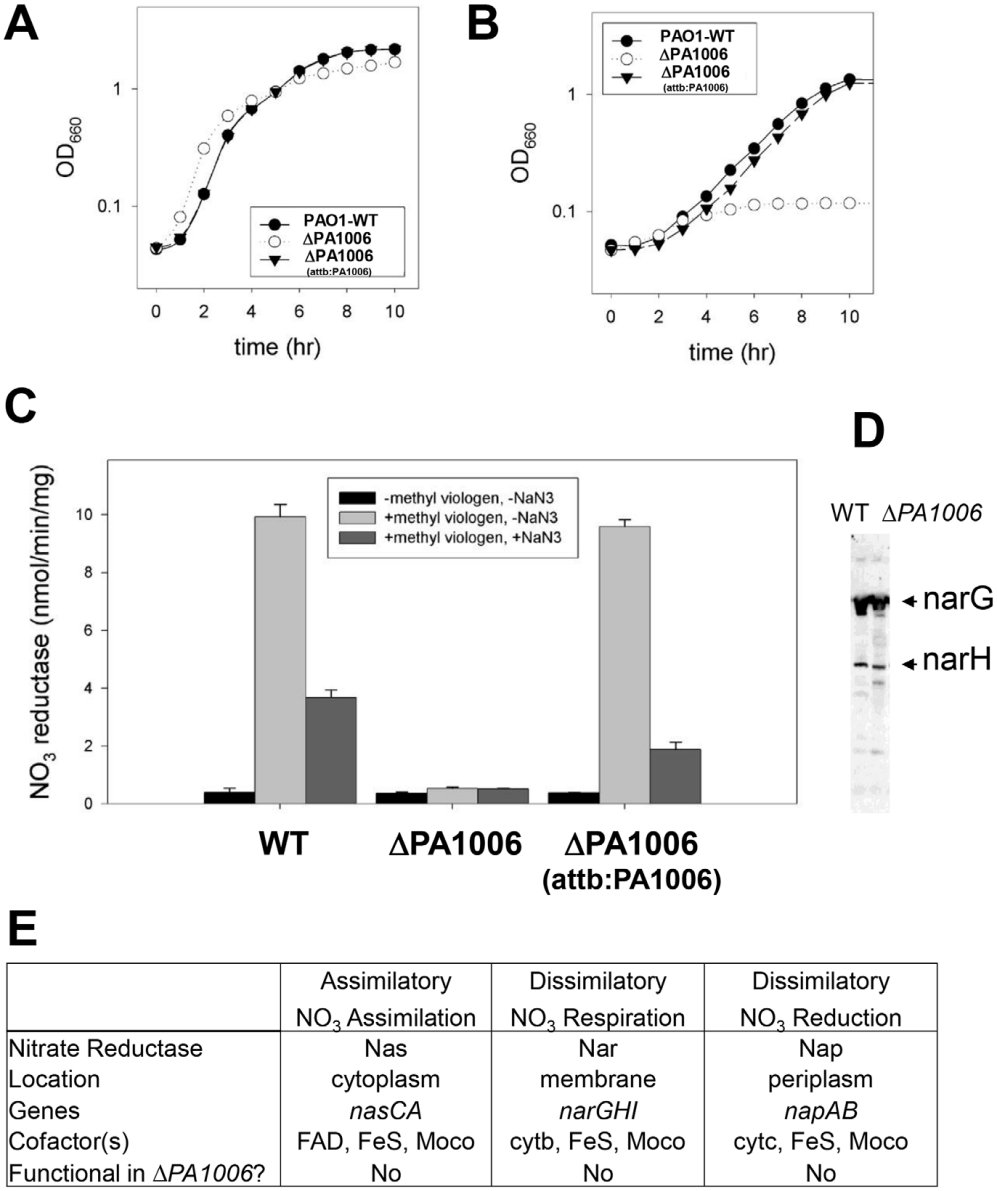
*PA1006* is critical for nitrate reductase activity. A/B) *PA1006* does not appear to affect aerobic growth in rich media but is required for anaerobic growth with nitrate. (•) WT; (○)Δ*PA1006*; (▾)Δ*PA1006:attb:PA1006*. Growth curves were performed in duplicate as indicated in the Methods average values are plotted. Data showed excellent agreement. C) Δ*PA1006* whole cell suspensions lack periplasmic and membrane nitrate reductase activity. D) Western blot with α-NarGH antisera of whole cell extract of wild-type (wt) and Δ*PA1006* (Δ) cells indicates that the membrane nitrate reductase is present but inactive. E) Summary of nitrate and nitrite reductases in *Pae*, their cofactors, and what is known about functionality in the Δ*PA1006* mutant.

In an accompanying manuscript we present a more detailed bioinformatics and biochemical analysis of PA1006 [24]. We provide evidence that the PA1006 protein is a persulfide-modified protein. In addition, we also demonstrate that PA1006 protein interacts with several molybdenum cofactor biogenesis proteins and is critical for molybdenum homeostasis.

In this paper, we demonstrate that PA1006 is essential for full virulence and biofilm formation. We also present data indicating that, in addition to a failure to utilize nitrate, loss of PA1006 causes multiple pleiotropic alterations in gene expression and metabolism including a prominent inability to utilize xanthine. The biological results presented in this manuscript are consistent with a role for PA1006 as a mediator of molybdenum homeostasis described in the accompanying paper [24]. Combined, these studies indicate that PA1006 is a novel target for inhibiting biofilm formation and virulence.

## Results

### *PA1006* is Critical for Nitrate Utilization

Previously we reported that a Tn mutant in the gene PA1006 was defective in anaerobic growth with nitrate as a terminal electron acceptor [21]. Since anaerobic growth and denitrification pathways appear to contribute to virulence; [8], [19], we set out to thoroughly characterize the role of PA1006 in *Pae*. To confirm specificity of the original Tn mutant and further investigate the function of PA1006, an in-frame deletion mutant (Δ*PA1006*) was constructed [25] in *Pae* strain PAO1. For anaerobic growth studies, aerobically grown overnight cultures of PAO1, the Δ*PA1006* mutant and a complemented mutant (Δ*PA1006*(*attB:PA1006*)) containing a single copy of the wild-type (WT) *PA1006* gene reintroduced onto the chromosome [26], were used to inoculate pre-reduced NY media supplemented with 100 mM KNO_3_ as the terminal electron acceptor. While aerobic growth was unaffected (Fig. 1A), in contrast to the WT strain PAO1, the Δ*PA1006* mutant was unable to grow anaerobically (Fig. 1B). Even after 24 hrs, no significant change in optical density was observed (data not shown). Single copy chromosomal complementation of the Δ*PA1006* mutant with the WT gene restored anaerobic growth (Fig. 1B) indicating that loss of *PA1006* was solely responsible for the anaerobic growth defect with nitrate.

Since *Pae* can use electron acceptor sources other than nitrate for anaerobic growth, the ability of the mutant to use alternative sources for anaerobic growth was analyzed. Notably, Δ*PA1006* was fully capable of growing anaerobically with nitrite and arginine (File S1). These data suggest that *PA1006* is specifically involved in the reduction of nitrate.

*Pae* can reduce nitrate either through assimilation or dissimilation. In *Pae,* dissimilatory reduction of nitrate (where nitrate acts as a terminal electron acceptor) normally occurs under anaerobic conditions but may also occur during aerobic growth [16], [27], [28]. Previously, microarray data indicated the nitrate reductase genes (*nar* and *nap*) are induced aerobically when 100 mM KNO_3_ is added to the media [17]. Therefore, nitrate reductase activity was assayed using whole cell suspensions from cultures grown aerobically in the presence of nitrate [29], [30]. Methyl viologen was used as the electron donor avoiding oxygen inhibition. Membrane (Nar) and periplasmic (Nap) activities were differentiated by their respective sensitivity or insensitivity to sodium azide (NaN_3_) which only inhibits the membrane bound nitrate reductase NarGHI [30]. Apparent Nar and Nap-dependent nitrate reductase activities were measurable in WT suspensions whereas Δ*PA1006* failed to show significant levels of either activity (Fig. 1C). Once again, the complemented Δ*PA1006* strain showed activity equivalent to WT (Fig. 1C).

The lack of nitrate reductase activity in the Δ*PA1006* strain may be due to the lack of expression of the *nar* and *nap* transcripts or proteins. To determine if the Δ*PA1006* strain expressed NarGH peptides, Western blot analysis was performed using rabbit polyclonal α-NarGH antisera. The Δ*PA1006* mutant displayed peptides that reacted with α-NarGH at levels comparable to WT (Fig. 1D). These data suggest that the Δ*PA1006* strain is capable of expressing the transcripts and protein subunits for the membrane tethered nitrate reductase NarGHI. In this case, the lack of nitrate reductase activity may be due to an inability to post-translationally process and/or assemble functional nitrate reductase enzymes.

Because nitrate reduction can also occur aerobically via the assimilatory Nas complex [31], and Nas also contains iron-sulfur clusters and molybdenum cofactor, we investigated if *PA1006* also played a role in this process. Δ*PA1006* was unable to grow aerobically when nitrate was provided as the sole nitrogen source (Files S1 and S8). These data indicate that the assimilatory nitrate reductase is also nonfunctional. Figure 1E is a compilation of *Pae* nitrate reductases and the cofactors required by each enzyme. A summary of the results of the growth and enzyme assays for the Δ*PA1006* strain are also shown. Loss of both assimilatory and dissimilatory nitrate reductase activities suggests that essential co-factors common to the nitrate reductases such as iron-sulfur clusters or molybdenum cofactor (we will use the generic term “MoCo” throughout this manuscript) may be lacking.

Given that the *PA1006* gene exists in an operon with *PA1007* which appears to be a transmembrane protein, it is possible that the PA1007 protein may cooperate with PA1006. To address this point, we obtained and confirmed the Δ*PA1007* transposon mutant from the PAO1 transposon insertion strain collection from the University of Washington. Since the Δ*PA1007* mutant was able to grow anaerobically with nitrate (data not shown), we did not perform additional analyses of the Δ*PA1007* mutant since it displayed non-equivalence with the Δ*PA1006* deletion strain.

### *PA1006* is Required for Virulence

*Pae* virulence has been examined by using different animal as well as plant and invertebrate models [32], [33], [34], [35], [36], [37], [38]. Since the *PA1006* Tn mutant previously displayed diminished virulence in the lettuce leaf model [21], we hypothesized that *PA1006* may play a role in *Pae* pathogenesis in other model systems.

**Figure 2:**
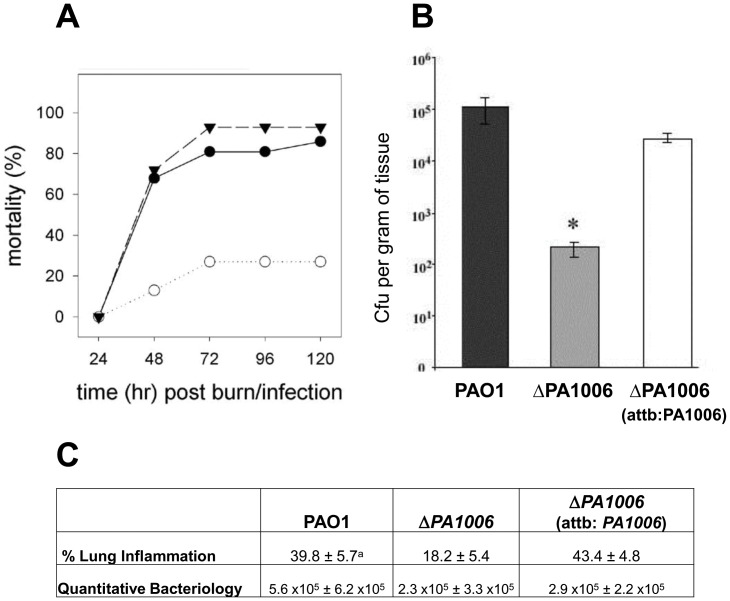
*PA1006* is necessary for virulence. A/B) Mouse thermal injury. A) Mice were scalded as described in Materials and Methods and a total of 1×10^3^ CFU of the *Pae* strain to be tested was injected subcutaneously in the burn eschar immediately after burning. Mortality was observed for 5 days post-burn/infection. Three separate experiments were conducted with each strain. The average percent mortality values are shown (** = p<0.01, n = 15/strain tested). (•) WT; (○)Δ*PA1006*; (▾)Δ*PA1006:attb:PA1006*. B) *PA1006* is required for full dissemination in the mouse thermal injury model. Quantitation of bacteria recovered from the livers of burned and infected mice. The number of CFU was calculated per gram of tissue. p = 0.04 (between PAO1 and PA1006), and p = 0.0002 (between PA1006 and the complemented strain), via student t-test. There were 10 mice total for each group. C) Effect of Δ*PA1006* on inflammation in a rat lung model of infection. ^a^Mean ± SD. ANOVA, Bonferroni multiple comparisons test indicated: P<0.001 for PAO1 vs *ΔPA1006*, P>0.05 for PAO1 vs Δ*PA1006*:*attb:PA1006*), and P<0.001 for Δ*PA1006* vs Δ*PA1006*:*attb:PA1006*).

The burned mouse model has been extensively used to examine the pathogenesis of *Pae* infection of burn wounds as well as to demonstrate the critical roles of virulence factors [38]. We employed the burned-mouse model to test the requirement of *PA1006* for *Pae* pathogenesis. Three groups of mice (n = 15 total) were burned and inoculated with approximately 3×10^3^ CFU of each of the strains as described in the materials and methods. Three separate experiments were performed with each strain. Survival of the mice was followed for a total of 5 days. [38]. As shown in Figure 2A, the parental WT PAO1 strain caused a significantly higher percent mortality than the Δ*PA1006* mutant (48 hours, p = 0.077, 72 hours, p = 0.0092, 96 hours, p = 0.0092, 120 hours, p = 0.0025). Importantly, complementation of the Δ*PA1006* mutant, with a single copy of the *PA1006* gene integrated into the chromosome, fully restored virulence to wild-type levels (93%) confirming a role for *PA1006* in *Pae* virulence and infection in this acute infection animal model. Reduced mortality observed by the *PA1006* mutant may have been the result of reduced dissemination (the systemic spread) of *Pae* within the mice. The systemic spread of PAO1, Δ*PA1006*, and the complemented mutant were examined by determining the numbers of CFU of these strains within the livers of the burned and infected mice. As shown in Figure 2B, at 24 h post burn infection, the numbers of bacteria (CFU per gram of tissue) that were recovered from the livers of the Δ*PA1006* mutant-infected mice were significantly lower than that of WT PAO1- or complemented mutant-infected, with almost a 3-log difference (p<0.01). Colonies were obtained from the livers of the mice were examined for the ability to grow anaerobically (data not shown). One hundred percent of the colonies examined retained the mutant phenotype, which confirmed that the Δ*PA1006* mutant did not revert to WT with concomitant ability to grow anaerobically. These results suggest that a mutation in *PA1006* interferes with the systemic spread of *Pae* within the burned and infected mice. Next, we chose to evaluate the role of *PA1006* in the agar bead rat lung model which is representative of the chronic infection observed in the CF lung [35]. Quantitative bacteriology (colony forming units recovered from tissue) showed that the *ΔPA1006* mutant survived as well as the WT bacteria when the mutant was embedded in agar beads and placed in rat lungs (Figure 2C). However, the Δ*PA1006* mutant appeared to cause significantly (p≤0.001) less damage to the lung tissue compared to the WT strain (Figure 2C). The WT strain showed ∼38% lung inflammation whereas the mutant displayed about half as much inflammation (∼16%). The decrease in inflammation was completely restored in the complemented strain (Δ*PA1006:attb:PA1006*), indicating expression of *PA1006* is required for full virulence of *Pae* in the lung model. These data suggest that *PA1006* may also be a critical determinant of chronic pathogenesis in the CF patient’s lung.

### *PA1006* is Critical for Biofilm Maturation

**Figure 3:**
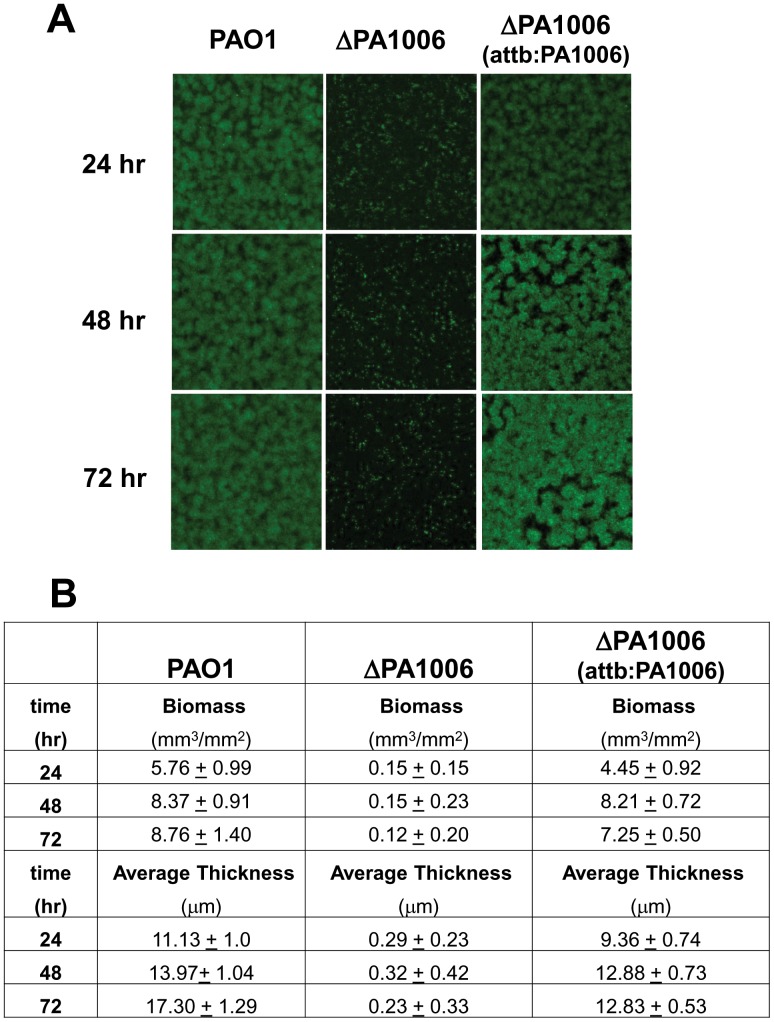
*PA1006* is required for biofilm formation in a flow through system. Biofilms were grown and analyzed as indicated in methods. A) Representative confocal images of *Pae* strains expressing GFP in flow through biofilms. Images were taken at random locations of each flow cell using confocal laser scanning microscope. B) COMSTAT analysis of biofilms.

Bacterial biofilms have been implicated in chronic lung infections caused by *Pae* [39], [40]. Since *PA1006* is required for anaerobic growth with nitrate and virulence, we hypothesized that *PA1006* could play a role in biofilm formation. To test this hypothesis, PAO1, Δ*PA1006*, and the complemented strains (Δ*PA1006*:*attB:PA1006*)) were tagged with the green fluorescent protein (GFP) [41]. We first tested biofilm formation in the “static-dish” assay which does not entail a flow system and relatively immature biofilms may form 24 h after initial inoculation. Confocal scanning laser microscopy (CSLM) and COMSTAT analysis were used to analyze the biofilms that covered the glass window at the bottom of the petri plate. In this system, WT, Δ*PA1006* mutant and complemented strains all appeared identical with mean thicknesses of approximately 10 µm each (File S2). Next, biofilm *maturation* was studied using a flow cell system which allows a relatively more mature biofilm to develop. Mature biofilms consist of a progression from microcolonies to tower-like structures that resemble mushrooms and are able to withstand the shear forces associated with the flow system. In addition to more complex architectures, water channels are also apparent. In the flow system assay, biofilm architecture was followed over a period of 3 days by CSLM and data analyzed using COMSAT. At 24 h and thereafter up to 72 h, the WT and complemented strains formed microcolonies and channels characteristic of mature *Pae* biofilms. In contrast to the static system, the Δ*PA1006* mutant displayed a severe defect in the continuous flow biofilm system (Fig. 3). Even after 72 h, very few bacteria were observed in the Δ*PA1006* chamber and the few cells present appeared as a thin layer of undifferentiated cells that failed to form microcolonies. Quantitative analysis with COMSTAT confirmed that the Δ*PA1006* mutant displayed severely reduced biomass, lower average thickness, and lower surface coverage compared to WT and complemented strains (Fig. 3). The calculated roughness coefficient, reflecting heterogeneity in thickness of the biofilm, was significantly greater for the Δ*PA1006* mutant (data not shown). Since complementation of Δ*PA1006* fully restored normal biofilm architecture, this dramatic phenotype is clearly due to the loss of *PA1006*. It should also be noted that the media used to grow biofilms in both static and flow-systems were identical and contained ammonia as the nitrogen source. Thus, failure of the Δ*PA1006* mutant to form a biofilm in the flow-system is not simply due to a failure to grow or utilize nitrate as a nitrogen source. More likely, the Δ*PA1006* mutant is less able to withstand the shear forces imposed by the flow system.

Since flagellar-based swimming and swarming motilities as well as type IV pili-mediated twitching motility are important for *Pae* biofilm formation and dispersal [42], we investigated whether the Δ*PA1006* mutation influenced these forms of motility. The Δ*PA1006* mutant exhibited twitching (17 mm ±1 mm) and swimming (32 mm ±1 mm) motilities undistinguishable from the WT strain PAO1 (twitching; 18 mm ±1 mm) and (swimming; 32 mm ±1 mm), indicating that the defects in biofilm maturation are not due to deficiencies in motility. Next, we measured swarming. Initially, the Δ*PA1006* mutant appeared to fail to exhibit swarming motility compared to WT PAO1. However, the original swarm agar plates contained nitrate as the sole source of nitrogen and growth was minimal. Given the failure of the Δ*PA1006* mutant to utilize nitrate, we substituted glutamate as the nitrogen source. In this case, swarming of the Δ*PA1006* mutant (10 mm ±1 mm) was comparable to WT (9 mm ±1 mm). Since rhamnolipid production also affects virulence, biofilms, as well as swarming motility, we also examined rhamnolipid production. Cells were grown for 90 h in M 8 minimal salts-based media containing glucose and glutamate as the sole sources of carbon and nitrogen respectively [43] and rhamnolipid production was determined by extraction of culture supernatants with diethyl ether followed by the orcinol assay as previously described [44]. Similar to swarming, rhamnolipid production by the Δ*PA1006* mutant appeared equivalent to WT when glutamate was substituted for nitrate as the source of nitrogen (all cell culture supernatants contained ∼ 100±10 mg/mL rhamnolipids). These data indicate that the Δ*PA1006* mutant does not resemble other previously characterized mutants defective in biofilm formation, such as those deficient in flagella, Type IV pilus, GacA, or Crc which display poor surface attachment or alterations in swimming, swarming, or twitching [42], [45].

*Pae* virulence and biofilms have been affected by changes in or a loss of lipopolysachharide (LPS) [36], [44]. When LPS profiles were analyzed and compared, the parent, mutant, and complemented strains expressed identical LPS profiles, confirming that the reduced virulence and dissemination as well as failure to form a mature biofilm in the flow systems observed with the Δ*PA1006* mutant were not due to altered LPS expression (data not shown).

### Loss of *PA1006* Affects Global Gene Expression

The combination of reduced virulence and biofilm phenotypes displayed by the Δ*PA1006* mutant suggested that expression of additional genes or activities of other proteins may be influenced by *PA1006*. In addition, previous transcriptome analysis from our laboratory revealed that expression of greater than 500 genes were altered by the addition of nitrate at early stationary phase under aerobic conditions [17]. Since the Δ*PA1006* mutant failed to dissimilate or assimilate nitrate, we were interested to test the effect of nitrate on gene expression in the Δ*PA1006* mutant. Microarray analysis was performed on both WT and the Δ*PA1006* mutant grown aerobically in the presence and absence of nitrate (Tables 1, 2; Files S4, S5, S6). It should be noted that the list of genes presented in Table 1 is not exhaustive, but rather lists genes that showed robust changes or those whose products are involved in denitrification, or represent known or putative virulence factors. The complete set of genes that showed altered expression can be found in Files S4 and S5.

**Table 1:** Notable nitrate metabolism and virulence genes whose expression levels are altered in the ΔPA1006 mutant in the absence of nitrate.

ORF	Gene	Fold change	Protein description
PA3870	*moaA1*	+17.6	molybdopterin biosynthetic protein A1
PA3871		+21.5	probable peptidyl-prolyl cis-trans isomerase
PA3872	*narI*	+22.2	respiratory nitrate reductase gamma chain
PA3873	*narJ*	+26.9	respiratory nitrate reductase delta chain
PA3874	*narH*	+34.2	respiratory nitrate reductase beta chain
PA3875	*narG*	+38.0	respiratory nitrate reductase alpha chain
PA3876	*narK2*	+51.8	nitrite extrusion protein 2
PA3877	*narK1*	+63.9	nitrite extrusion protein 1
PA3911	*yhbT*	+7.0	conserved hypothetical protein
PA3912	*yhbV*	+11.2	conserved hypothetical protein
PA3913	*yhbU*	+15.1	probable protease
PA3914	*moeA1*	+209.4	molybdenum cofactor biosynthesis
PA3915	*moaB1*	+52.8	molybdopterin biosynthetic protein B1
PA3916	*moeE*	+11.6	molybdopterin converting factor
PA3917	*moaD*	+10.1	molybdopterin converting factor,
PA3918	*moaC*	+10.5	molybdopterin biosynthetic protein C
PA0074	*ppkA*	−2.4	Ser/Thr protein kinase- TypeVI SS
PA0075	*pppA*	− 2.0	Ser/Thr protein phosphatase- TypeVI SS
PA0077	*icmF1*	− 2.0	TypeVI SS component
PA0078		− 2.4	TypeVI SS component
PA0085	*Hcp1*	− 2.0	TypeVI SS component
PA0090	*clpV1*	− 2.0	TypeVI SS component
PA0262		− 2.0	TypeVI SS component
PA3294		− 2.0	TypeVI SS component

**Table 2:** Notable nitrate metabolism and virulence genes whose expression levels are altered in the ΔPA1006 mutant in the presence of nitrate.

ORF	Gene	Fold change	Protein description	
PA0996	*pqsA*	+11.8	probable coenzyme A ligase	
PA0997	*pqsB*	+6.4	3-oxoacyl-[acyl-carrier-protein	
PA0998	*pqsC*	+6.0	3-oxoacyl-[acyl-carrier-protein	
PA0999	*pqsD*	+4.6	3-oxoacyl-[acyl-carrier-protein] synthase III	
PA1000	*pqsE*	+9.2	quinolone signal response protein	
PA1001	*phnA*	+8.0	anthranilate synthase component I	
PA1002	*phnB*	+5.1	anthranilate synthase component II	
PA1172	*napC*	+2.4	cytochrome c-type protein	
PA1173	*napB*	+3.4	Nitrate reductase cytochrome c-type subunit	
PA1174	*napA*	+3.7	periplasmic nitrate reductase protein	
PA1175	*napD*	+4.8	NapD protein of periplasmic nitrate reductase	
PA1176	*napF*	+4.3	ferredoxin protein NapF	
PA1177	*napE*	+4.6	periplasmic nitrate reductase protein	
PA1871	*lasA*	+13.1	LasA protease precursor	
PA2193	*hcnA*	+8.1	hydrogen cyanide synthase HcnA	
PA2194	*hcnB*	+5.3	hydrogen cyanide synthase HcnB	
PA2195	*hcnC*	+5.1	hydrogen cyanide synthase HcnC	
PA3478	*rhlB*	+30.0	rhamnosyltransferase chain B	
PA3479	*rhlA*	+10.5	rhamnosyltransferase chain A	
PA3724	*lasB*	+3.8	elastase LasB	
PA3878	*narX*	+2.1	two-component sensor NarX	
PA5170	*arcD*	+3.3	arginine/ornithine antiporter	
PA5171	*arcA*	+3.0	arginine deiminase	
PA5172	*arcB*	+2.3	ornithine carbamoyltransferase, catabolic	
PA0509	*nirN*	− 9.4	probable c-type cytochrome	
PA0510	*nirE*	− 9.2	probable methyltransferase	
PA0511	*nirJ*	− 9.1	heme d1 biosynthesis protein	
PA0512	*nirH*	− 10.1	conserved hypothetical protein	
PA0514	*nirL*	− 11	heme d1 biosynthesis protein	
PA0515	*nirD*	− 8.3	probable transcriptional regulator	
PA0517	*nirC*	− 6.2	probable c-type cytochrome precursor	
PA0518	*nirM*	− 7.7	cytochrome c-551 precursor	
PA0519	*nirS*	− 7.0	nitrite reductase precursor	
PA0520	*nirQ*	− 10.6	regulatory protein	
PA0521	*norE*	− 56.9	probable cytochrome c oxidase subunit	
PA0522		− 15.3	hypothetical protein	
PA0523	*norC*	− 294.4	nitric-oxide reductase subunit C	
PA0524	*norB*	− 348.9	nitric-oxide reductase subunit B	
PA0525	*norD*	− 306.6	probable denitrification protein	
PA0526		− 7.4	hypothetical protein	
PA3391	*nosR*	− 140.3	regulatory protein NosR	
PA3392	*nosZ*	− 165.9	nitrous-oxide reductase precursor	
PA3393	*nosD*	− 42.7	nitrous oxidase accessory protein	
PA3394	*nosF*	− 71.8	ABC-type transport system,	
PA3395	*nosy*	− 117.9	copper enzyme maturation, permease	
PA3396	*nosL*	− 124.7	predicted lipoprotein, nitrous oxide reduction	

In the absence of nitrate supplementation (NY media), 73 and 16 genes displayed decreased or increased expression respectively in the Δ*PA1006* mutant (using a 2-fold cutoff). Especially notable were significantly higher levels of RNAs for the Nar gene cluster which codes for the membrane nitrate reductase, accessory factors, and nitrate transport proteins (PA3871-PA3877), as well as genes involved in molybdopterin biosynthesis (PA3914-18; and PA3870; Table 1). This was unexpected because nitrate reductase activity appeared to be lacking in the Δ*PA1006* mutant. Also notable was the fact that the *ΔPA1006* mutant also displayed reduced expression of several Type-VI Secretion System components (PA0074-75, PA0077-78, PA0085, PA0090, PA0262, and PA3294). Since Type-VI Secretion System components have already been implicated in *Pae* virulence [46] these data may be correlated with the loss of virulence potential displayed by the Δ*PA1006* mutant in the mouse burn and rat lung.

Next, we examined the effect of NO_3_ on gene expression. 230 and 328 genes displayed decreased or increased expression respectively (with a 2-fold cutoff) in the *ΔPA1006* mutant compared to the WT when 100 mM KNO_3_ was added to the growth media. In this case, other denitrification pathway genes such as nitrite reductase (Nir), nitric oxide reductase (Nor), and nitrous oxide reductase (Nos) displayed dramatically reduced expression in the Δ*PA1006* mutant (PA0509-26 and PA3391-96 clusters; Table 2). Generally, compared to WT, it appears that denitrification pathway enzymes downstream of Nar are not induced by nitrate in the Δ*PA1006* mutant. Loss of *PA1006* also altered the expression of several other notable genes (Table 2). For example, Δ*PA1006* cells also showed increased expression of the *Pseudomonas* quinolone signal (PQS) biosynthesis genes (PA0996-PA1001) as well as anthranilate components (PA1001-1002) when grown in the presence of nitrate. Rhamnolipid biosynthesis (*rhlAB; PA3479-78*) and elastase (*las/PA1871* and *lasB/PA3724*) also showed significantly increased expression in the Δ*PA1006* mutant compared to WT when nitrate was added. It is notable that *rhlAB* levels appeared to be increased since rhamnolipid production appeared unaltered (see above). This apparent discrepancy may be due to differences in growth and media conditions required to measure rhamnolipid production versus microarray analyses. Several other QS-regulated genes [47], [48], [49] including hydrogen cyanide biosynthesis components (*hcn*A-C; PA2193-95), the cbb-3 type cytochrome c oxidase (PA4133), a putative sulfite reductase (PA4130), cyt P450 (PA3331), and *fabH2* (PA3333) also displayed aberrantly high expression levels when the Δ*PA1006* mutant was grown with nitrate (Table 1). Normally, in the WT strain, QS and denitrification (nitrate-responsive) genes display reciprocal expression patterns [17], [47], [50]. That is, in the presence of nitrate, QS regulated genes are normally repressed. However, in the Δ*PA1006* mutant, many QS genes that are normally repressed by nitrate supplementation remain expressed at relatively high levels.

In addition to nitrate-responsive and QS-regulated genes, we also observed altered gene expression of several Fur-regulated genes [51] in the Δ*PA1006* mutant only when grown in the presence of nitrate (File S6). In WT, Fur-regulated genes are derepressed when nitrate is added, whereas these genes remain repressed in the Δ*PA1006* mutant (suggesting that when nitrate is added, Fe is replete in the Δ*PA1006* mutant yet depleted in WT). The IscR operon consisting of genes involved in sulfur trafficking (*PA3815-PA3808*) is another notable example of a set of genes whose expression remains at the basal level in Δ*PA1006* but increased levels in WT when grown with nitrate. Taken together, the data suggests that the *PA1006* mutant fails respond to nitrate.

While we cannot presently explain deregulation of the QS genes, inactivation by reactive nitrogen species such as NO may account for the effects seen with Fur-regulated genes. Consistent with *E. coli* studies [52], in WT *Pae* grown with nitrate, denitrification will generate reactive nitrogen species such as NO which can inactivate Fur allowing the expression of Fur-regulated genes. In the Δ*PA1006* mutant, reactive nitrogen species such as NO would not be generated from NO_3_, since the mutant cannot utilize NO_3_, and Fur-regulated genes remain repressed. These data suggest that *Pae* may employ strategies to balance QS signaling with denitrification needs and products, and vice versa. Disruption of this balance may also prevent growth as a biofilm or in a host organism.

Transcriptional changes observed by the microarray analyses were validated using transcriptional *lacZ* fusions. While the WT strain displays reduced expression of *rhlA, rhlI,* and *rhlR,* when grown aerobically in the presence of nitrate, the Δ*PA1006* mutant continues to express these transcripts at similar levels regardless of whether nitrate is added to the growth medium. (File S3). These data confirm that the *ΔPA1006* mutant over-expresses genes required for rhamnolipid production when grown in the presence of nitrate.

### *PA1006* Affects Production of PQS

**Figure 4:**
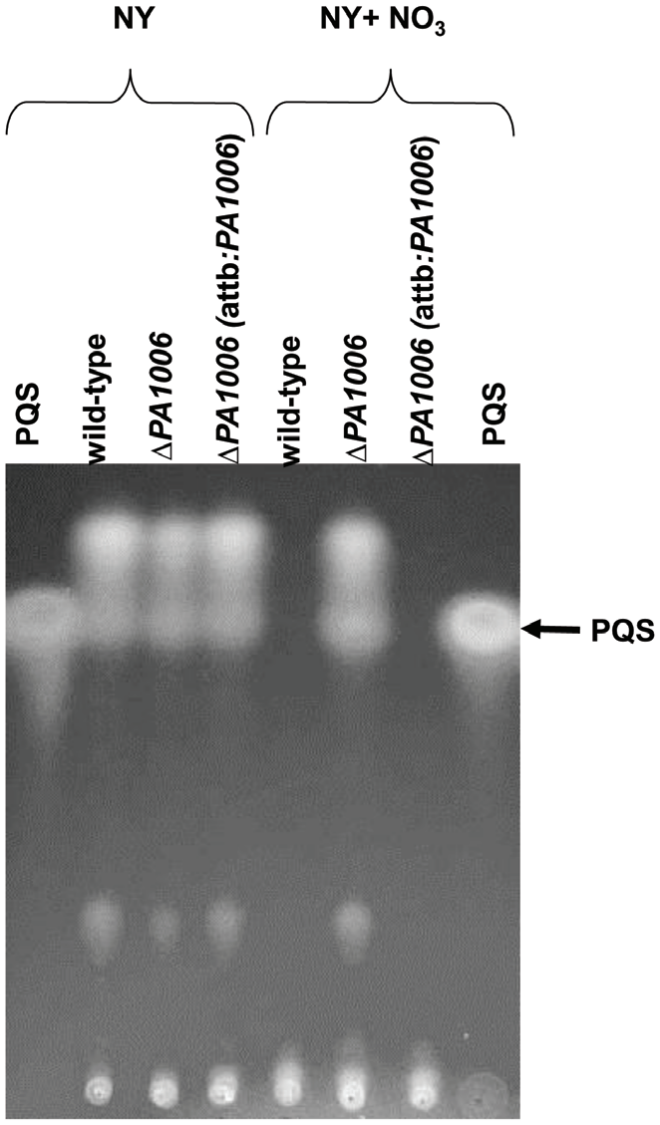
*PA1006* affects PQS production. PQS production by *Pae* strains. PQS samples extracted from 24 h cultures were analyzed by TLC. The arrowhead indicates the position of PQS.

Since Δ*PA1006* cells also showed an increased expression of the PQS biosynthetic genes in the presence of nitrate, we examined PQS production by Δ*PA1006* compared to WT. TLC analysis revealed that Δ*PA1006* cells continue to produce PQS when grown in the presence of nitrate, whereas nitrate suppresses PQS production in WT and complemented strains (Fig. 4). These data further validate the microarrray analysis results and also show that PQS production may be regulated by nitrate availability (or downstream products of denitrification). Since PQS levels were altered in the presence of nitrate, we also evaluated the production of other QS signaling molecules 3-oxy-C12-homoserine lactone (C12-HSL), and 3-oxy-C4-homoserine lactone (C4-HSL). Both C12-HSL and C4-HSL levels appeared similar to WT (data not shown) unlike the PAO-JP-2 mutant (Δ*lasI*Δ*rhlI)* which fails to produce autoinducers [53]. Therefore, a failure to produce autoinducers can be ruled out as the root cause of biofilm instability in the Δ*PA1006* mutant.

While the reason for aberrant regulation of PQS in the presence of nitrate remains unknown, PQS deregulation may also have a significant impact on the Δ*PA1006* mutant’s metabolism. Aside from its role as an QS signal [54], recent studies revealed other functions for PQS. It has been shown that excess PQS appears to block denitrification by inhibiting nitrate reductase activity [55]. PQS can also bind to iron (III) and may sequester it near the surface of the cell [56]. Since addition of excess iron relieved the inhibition of denitrification activity by PQS, it was suggested that free PQS levels must be tightly regulated to control iron (III) surface levels or availability during dentrification [55]. In order to rule out the possibility that iron sequestration by PQS was causing defects in nitrate reductase, we supplemented NY+NO_3_ media with additional iron (50 µM each of FeSO_4_ and FeCl_3_) and tested anaerobic growth. Additional iron failed to restore anaerobic growth/nitrate utilization by the Δ*PA1006* mutant (data not shown) indicating that chelation of iron by PQS is not likely the root cause of a failure to grow anaerobically. The failure to rescue nitrate utilization with additional iron also agrees with the microarray data which clearly show that, in the presence of nitrate, Fur-regulated genes remain repressed in the Δ*PA1006* mutant (even though extracellular PQS remains in excess) indicating that intracellular iron is not being significantly depleted by the PQS.

### *PA1006* Displays an Altered Metabolic Profile

The microarray gene expression profile indicated that the Δ*PA1006* mutant displayed changes in enzymes associated with metabolism. Therefore, to study the Δ*PA1006* mutant’s metabolic capacity in more detail, Biolog Phenotype Microarrays™, which have been used successfully to probe *Pae* metabolism [57], [58], were used to comparatively assess the metabolic requirements of the Δ*PA1006* PAO1 mutant versus WT strain. Biolog results are provided in File S7. In addition, functional annotation of genes that showed expression changes in microarray experiments and substrates that showed altered utilization in Biolog experiments were superimposed upon KEGG pathway maps [59] and this extended analysis is provided in Files S7, S8, S9. Several global observations could be made which indicated several trends. For example, many TCA cycle (or nearby) intermediates such as succinate, citrate, fumarate, malate, and α-ketoglutarate could not be utilized as effectively as sole sources of carbon by the Δ*PA1006* mutant. In addition, the Δ*PA1006* mutant appeared less capable of utilizing acetate as a sole source of carbon suggesting that the key metabolic enzyme isocitrate lyase (encoded by *PA2634*/*aceA*) or other glyoxylate cycle enzymes may also be compromised [60], [61]. Similarly, several purines such as adenosine, guanine, gaunosine, xanthine, and xanthosine were not utilized as effectively as sole sources of nitrogen by the Δ*PA1006* mutant. In fact, the most severe deficiencies in Δ*PA1006* were found with nitrate and xanthine as nitrogen sources. When nitrate or xanthine was supplied as the nitrogen source, the tetrazolium dye color did not exceed background in Δ*PA1006* whereas WT gave a relatively robust signal (we also confirmed in separate experiments that the Δ*PA1006* mutant is unable to grow on hypoxanthine as a sole nitrogen source (data not shown)). These data may be revealing with regard to PA1006 function since an inability to utilize xanthine combined with a loss of all nitrate reductase activities is consistent with ‘shared nitrate reductase mutants” where MoCo is lacking [62].

## Discussion

The goal of these studies was to shed light on the biological role of the PA1006 protein in *Pae* metabolism and virulence.

Our data suggest the *ΔPA1006* mutant phenotype is due to *metabolic differences*. Since the most robust changes in gene expression in the *ΔPA1006* mutant reflect a loss of nitrate responsiveness (and lack of coordination of denitrification with QS regulation) it is likely that a loss of signaling via denitrification pathway intermediates contributes significantly to the *ΔPA1006* mutant phenotype. A concrete example of this idea is inactivation of Fur by reactive nitrogen species such as NO and how it affects Fur-regulated gene expression. *E. coli* studies [52] predict that *Pae* denitrification would generate reactive nitrogen species such as NO which can inactivate Fur allowing the expression of Fur-regulated genes. In the Δ*PA1006* mutant we find that Fur-regulated genes remain repressed (in contrast to WT which are induced by nitrate) most likely because reactive nitrogen species such as NO are not generated from NO_3_ since the mutant cannot reduce NO_3_. In further support of the idea that a loss of denitrification signaling pathways critically alters *Pae* metabolism, Δ*narGH* and Δ*nirS* mutants were also shown to be defective in biofilm formation and virulence [19], [63]. Reciprocally, addition of *excess* denitrification intermediate NO was also found to disperse biofilms that had already formed [20]. Therefore, the differences in Fur-regulated gene expression that we observe in the Δ*PA1006* mutant may only be indicative of other significant changes affected by loss of denitrification that we cannot appreciate presently.

Given the additional alterations in metabolic gene expression and substrate utilization profile revealed by the Biolog studies, metabolic changes beyond the loss of denitrification may further contribute to the Δ*PA1006* mutant phenotype. For example, recent publications have revealed that excess carbon sources (such as the TCA cycle intermediate succinate), as well as D-amino acids (generated by racemases) are all effective in dispersing biofilms [64], [65]. Similarly, the loss of virulence displayed by the Δ*PA1006* mutant may also result from global metabolic changes. For example, two genes (PA0887 and PA3234) that showed *decreased* RNA levels in the Δ*PA1006* mutant in the presence of nitrate were reciprocally *up-regulated* in CF clinical isolates of *Pae* in three separate reports [12], [13], [66], [67]. While the function of PA3234 is unknown, PA0887 or AcsA (acetyl-coA synthase) is particularly interesting because acetyl-coA is an important metabolite that connects many aspects of central metabolism [68]. It may also be noteworthy that the metabolite/signaling molecule c-di-GMP has been shown to mediate NO-induced dispersal of biofilms [69] and is also linked to Type VI secretion systems [70], both of which are altered in the Δ*PA1006* mutant.

When we initiated the studies presented in this manuscript, the function of PA1006 could not be predicted using a bioinformatics approach and the robust phenotypes we observed in virulence and biofilm assays were entirely unexpected. In the companion manuscript, we provide evidence that PA1006 may function in a similar but not identical manner compared to YhhP/TusA [24]. First, we clearly demonstrate that PA1006 is modified by a persulfide group in *Pae* on the highly conserved Cys that in YhhP/TusA is also modified as a persulfide. Although sulfur trafficking via persulfide sulfur carriers is a relatively new concept, many metabolites such as Fe-S clusters, MoCo, thiamine, and 2- or 4-thiouridine derivatives are concrete examples of metabolites that require sulfur trafficking for their biosynthesis and additional metabolites may be forthcoming [71]. In this light, one possible explanation of our data is that the loss of *PA1006* may affect sulfur trafficking for MoCo biosynthesis or Fe-S cluster assembly. Both MoCo and Fe-S clusters are essential cofactors for electron transfer in the catalytic subunits of assimilatory and dissimilatory nitrate reductase enzyme complexes, the periplasmic nitrate reductase of *Pae* (see Fig. 1E and ref. [72]), as well as xanthine dehydrogenase [73], and all of these activities are lacking in the Δ*PA1006* mutant. In the accompanying paper, we also demonstrate that PA1006 interacts with several MoCo biosynthesis factors, and that PA1006 is required for molybdenum homeostasis [24]. These data are consistent with the loss of nitrate reductase activity and a failure to utilize xanthine/hypoxanthine displayed by the Δ*PA1006* mutant detailed herein. Together, these studies provide a novel and unexpected connection between metabolism, biofilm formation, and virulence.

## Methods

### Bacterial Strains and Growth Conditions

*P. aeruginosa* strain PAO1 was maintained at 37°C on NY (2.5% nutrient broth and 0.5% yeast extract) agar plates supplemented with 100 mM KNO_3_ or peptone tryptic soy broth (PTSB) agar plates [74]. The transposon mutant containing a disruption in NapA was obtained from PA14 Mutant Library (http://ausubellab.mgh.harvard.edu/cgi-bin/pa14/home.cgi) [75]. Anaerobic media preparation and cell cultivation were performed as previously described [47]. Anaerobic growth of *P. aeruginosa* was performed in a Coy anaerobic chamber (85% N_2_, 10% H_2_, and 5% CO_2_; Coy Laboratories Inc.). For anaerobic growth, Luria-agar base media was used with the electron acceptors KNO_3_ or KNO_2_ at 100 mM and 10 mM final concentrations respectively and arginine at a concentration of 40 mM [21], [76]. For biofilm experiments, bacteria were grown overnight in Luria-Bertani (LB), and modified FAB medium with 10 mM sodium citrate as the carbon source [41] was used as the flow-through media. Aerobic growth was determined by plating on VBMM plates containing either 100 mM KNO_3_, 10 mMKNO_2_, or NH_4_Cl, as the sole nitrogen source, and incubating under aerobic conditions for 48 h [62], [77]. *E. coli* cultures were grown using LB agar plates and broth. When necessary, media was supplemented with ampicillin (100 µg per ml) for *E. coli* and carbenicillin (200 µg per ml) for *Pae*.

Aerobic growth was determined by plating on VBMM plates containing either 100 mM KNO_3_, 10 mMKNO_2_, or NH_4_Cl, as the sole nitrogen source, and incubating under aerobic conditions for 48 h [62], [77].

### Recombinant DNA Techniques

Cloning was performed using standard methods [78]. *Pseudomonas* chromosomal DNA was isolated using a previously described method [79]. DNA sequences of all constructs were obtained using automated DNA sequencing with Big Dye terminator mix (U. of Rochester Core Facility).

### Construction of the Δ*PA1006* Mutant

An in-frame deletion of *PA1006* (Gene ID: 879448) was created by overlap extension PCR as described previously [80], [81] with the following modifications. Two DNA fragments consisting of a region of PAO1 genomic DNA containing approximately 1.5 kb upstream and 0.75 kb downstream respectively, of *PA1006* were obtained from PCR-amplification of p1E1, a plasmid from the initial complementation of the Tn mutant with a transposon insertion in *PA1006* (unpublished), with primers sets A [5′-CAGCTATGACCATGATTACGAATTCG-3′] and B [5′-GACAGCCTCTCCCGAAGGGC-3′] and C [5′-GCCCTTCGGGAGAGGCTGTCATGATCAAGGTGTTACGC-3′] and D [5′-AAACGACGGCCAGTGCAAGCTT-3′]. Following electrophoresis, amplicons were gel extracted and a mixture of these DNA fragments (∼100 ng each) was used as template in a third PCR amplification using primers A and D. The final product containing the in-frame deletion of *PA1006* was digested with *Hind*III and *Kpn*I and ligated with *Hind*III/*Kpn*I-digested pEX18Ap [25]. This plasmid was then mobilized from *E. coli* SM10 into PAO1 [25]. Conjugants were selected on VBMM [25] containing carbencillin (200 µg per ml) to select for incorporation of the plasmid carrying the deletion construct. To resolve the plasmid sequence, colonies were plated onto VBMM containing 5% sucrose. The deletion was verified using PCR and Southern blotting.

### Complementation of Δ*PA1006* Unmarked Deletion Mutant

For single copy chromosomal insertion complementation, the plasmid p1E1 (see above) was used to subclone the complete *PA1006* ORF, its putative promoter region, and flanking DNA with flanking *Pst*I sites. The *Pst*I fragment was then moved into pUCP18. pUCP18 containing the PA1006 fragment was digested with *Sph*I and a resulting 650 bp fragment was made blunt with T4 polymerase and ligated into *EcoR*V-digested miniCTX1 [26]. The resulting plasmid was verified by restriction analysis and PCR and introduced into *E. coli* strain SM10. This strain was used to mobilize the complementing fragment into the Δ*PA1006* mutant by conjugation, as described previously [26]. Chromosomal insertion at the *attB* site was verified by PCR and Southern blot analyses.

### Virulence Assays

#### Lethality in burned mouse model

The virulence of the PAO1 strains was examined by using the burned-mouse model as described [82]. Briefly, *Pae* strains were subcultured and grown at 37°C to an optical density at 540 nm of approximately 0.9. Cells were then pelleted, washed, and serially diluted in PBS. Thermal injury was induced by scalding 15% of the body surface in 90°C water for 10 s and 1×10^3^ CFU of each *Pae* strain was injected subcutaneously within the burn eschar. Mortality was monitored for 5 days. The Fisher’s exact test (Statview; Abacus Concepts, Inc.) was used to determine the significant differences between groups of mice for the mortality experiments.

#### Dissemination in burned mouse model

At 24 h post burn infection, the mice were euthanized by intracardial injection of 0.2 ml of Sleepaway (sodium pentobarbital-7.8% isopropyl alcohol euthanasia solution; Fort Dodge Laboratories, Inc. Fort Dodge, Iowa). The livers of each animal were obtained from both control and challenged mice. Livers were weighed, suspended in PBS, and homogenized (Wheaton overhead stirrer; Wheaton instruments, Millville, N.J.). A 100 µl aliquot of each homogenate was plated on LB agar plates to determine the number of post burn infection CFU. The number of CFU from each liver was calculated per gram of tissue. The student t-test was used to determine the significant differences between the strains.

#### Rat lung model

The rat chronic-lung infection assay was performed as previously described [32], [35]. Briefly, rats (Sprague-Dawley male 180–200 g) were inoculated with approximately 1×10^4^ cfu of either the wild-type bacteria, the Δ*PA1006* mutant, or the complemented mutant (Δ*PA1006* (*attB:PA1006*)) encased in agar beads and placed in the left lungs of the rats. At 10 days post infection, lungs were removed for either quantitative bacteriology or quantitative pathology analysis [32], [35]. Sections were coded, analyzed and scored as previously described [32], [35].

### Ethics Statement

All efforts were made to minimize suffering in all animal experiments. Mouse experiments were carried out in strict accordance with the recommendations in the Guide for the Care and Use of Laboratory Animals of the National Institutes of Health. The protocol was approved by the Institutional Animal Care and Use Committee of Texas Tech University Health Sciences Center (Protocol Number: 07044). Rat experiments were conducted according to the guidelines of the Canadian Council of Animal Care for the care and use of experimental animals approved by the University of Calgary Animal Care Committee (Protocol Number: M08089).

### Biofilm Analysis

#### Static dish biofilms

Static dish biofilms assays were carried out as described previously [19]. Plasmid pTDKgfp [41], which constitutively expresses green fluorescent protein (GFP), was introduced into each strain by electroporation. Overnight cultures containing the plasmid pTDK-gfp were grown in LB and then diluted into modified FAB-citrate media (final O.D._600_ = 0.5) prior to inoculation. Next, 2.0 mL of cells (O.D_600_ = 1.2) were inoculated into 35 mm petri plates with glass coverslips incorporated into the bottom chamber (Matek Corp. ) to allow visualization. After 24 h incubation at 37°C under aerobic conditions, media was carefully removed and replaced with three consecutive 1.0 mL portions of modified FAB-citrate. Next, biofilms were visualized by a Leica inverted laser scanning confocal microscopy (CSLM). Analysis of biofilm images was done with COMSTAT [85]. Five separate fields measuring 250−×250 µm were analyzed for each condition.

#### Continuous flow biofilms

Biofilms were cultivated in flow chambers at 37°C as described [41], with slight modifications. Plasmid pTDKgfp [41], which constitutively expresses GFP, was introduced into each strain by electroporation. Early stationary phase cultures (OD_600_ ∼1.2) were used as inoculum. After bacteria adhered to the glass slides for 2 h, medium was pumped at a constant flow rate of 0.2 ml/min. At the designated time, flow cells were visualized with a Leica laser scanning confocal microscope (URMC Pathology Imaging Core, Rochester, NY). Analysis was performed as described above for static biofilms. It is worth noting that in the FAB-citrate media, the nitrogen source is ammonia and not nitrate. Moreover, we found that the Δ*PA1006* mutant was able to utilize ammonia as a sole source of nitrogen in minimal media (data not shown). Therefore, in these biofilm studies, a lack of nitrate utilization displayed by the Δ*PA1006* mutant would not prevent the Δ*PA1006* mutant from obtaining nitrogen.

### Twitching and Motility Assays

Twitching motility was assessed on LB (1%) agar plates at 37°C as described by Glessner [83]. Swimming assay was performed via the method described by Rashid by inoculating plates containing 1% tryptone, 0.5% NaCl, and 0.3% agar and incubating at 30°C for 16 h. [84]. Swarming motility was performed as described by Kohler et al. [43].

### Nitrate Reductase Activity

Nitrate reductase activity was determined in whole cell suspensions [29], [30]. Cultures were grown aerobically in NY with 100 mM KNO_3_. To inhibit protein synthesis, 1.5 ml of 50 µg/ml chloramphenicol was added to 1.5 ml of culture. If indicated, 1.5 µl of 50 mM NaN_3_ was added to inhibit the membrane nitrate reductase NarGHI. Cells were centrifuged, washed twice, and re-suspended in an equal volume of 50 mM phosphate buffer, pH 7.2, and optical density at 660 nm (OD_660_) was determined. An 800 µl aliquot of cells was mixed with 100 µl freshly prepared, 0.5 mg/ml methyl viologen solution. Nitrate reduction was initiated by adding 100 µl of a solution containing 4 mg/ml sodium dithionite, 4 mg/ml sodium bicarbonate, and 100 mM KNO_3_. Control reactions replaced sodium dithionite with water. Reactions were incubated at room temperature for 5 min, and stopped by vortexing until the solution became clear, indicating the electron donor was oxidized. 1 ml of 1% w/v sulfanilic acid in 20% HCl was added immediately to the stopped reaction, and vortexed for 15 s. 1 ml of 1.3 mg/ml N-(1-naphthyl) ethylenediamine-HCL was added to allow formation of red azo dye, and the suspension was centrifuged to pellet debris. Optical density at 540 nm (OD_540_) of the supernatant was measured spectrophotometrically to quantitate dye formation, and optical density at 420 nm (OD_420_) was measured to account for absorbance due to light scattering by residual cells or cell fragments. Activity is expressed in arbitrary units based on the formula 100× [OD_540_– (0.72×OD_420_)]/(T×V×OD_660_) [85]. T = time in minutes, V = volume of reaction used in ml, OD_660_ corresponds to the optical density of the culture used. Assays were performed in triplicate in three separate experiments. To detect NarGH protein, Western Blot analysis was performed. Overnight cultures grown in LB were diluted to OD_600_ ∼0.05 in NY+100 mM KNO_3_ broth and were grown to OD_600_ ∼1.0. Cells were pelleted and resuspended in SDS gel loading buffer containing 1% SDS (3× volume of cell pellet) and boiled. Approximately 20 mg of total cell lysate were resolved by SDS PAGE. Rabbit polyclonal α-NarGH primary was kindly provided by Axel Magalon (CNRS, Marseille FR). The secondary antibody was conjugated to HRP and the blot was developed using chemilluminescence.

### Microarray Analysis

Parental PAO1 WT and mutant Δ*PA1006* strains were grown aerobically at 37°C in nutrient broth supplemented with yeast extract containing 100 mM KNO_3_ or lacking KNO_3_. RNA was extracted at early stationary phase (OD_600_ = 1.2) using TRI-regent (Ambion, Austin, TX). Residual DNA was removed with amplification grade DNAse I (Invitrogen) and RNA was concentrated using a MinElute kit (Qiagen). RNA integrity was assessed using a Bioanalyzer (Agilent Technologies, Foster, City, CA). cDNA synthesis, labeling, fragmentation, hybridization, and chip scanning was performed as described previously [47]. Microarray Suite 5.0 (Affymetrix, Santa Clara, CA), GeneSpring 6.2 (Silicon Genetics, Redwood City, CA) and Significance Analysis of Microarrays (SAM), version 1.15 [86] were used to analyze the data. Microarrays were globally scaled to a target intensity of 500 and filtered for transcripts present in two of three arrays. Statistical significance was determined using a t-test with p value of <0.05. Differentially expressed genes showing ≥2-fold change were filtered using the above criteria and are listed in Table 2. For SAM analyses, transcripts which demonstrated >2-fold change or greater and a false discovery rate of 5% were considered statistically significant. SAM analyses results are available in Files S4 and S5. Microarray data were deposited into Gene Expression Omnibus repository (GEO) at the National Center for Biotechnology Information (http://www.ncbi.nlm.nih.gov/) and may be found with the following accession numbers: GSM711446- PAO1 delta PA1006 strain grown in NY media, replicate 1; GSM711447- PAO1 delta PA1006 strain grown in NY media, replicate 2; GSM711448- PAO1 wild-type strain grown in NY media, replicate 1; GSM711449- PAO1 wild-type strain grown in NY media, replicate 2; GSM711450- PAO1 delta PA1006 strain grown in NY+NO3 media, replicate 1; GSM711451- PAO1 delta PA1006 strain grown in NY+NO3 media, replicate 2; GSM711452- PAO1 delta PA1006 strain grown in NY+NO3 media, replicate 3; GSM711453- PAO1 wild-type strain grown in NY+NO3 media, replicate 1; GSM711454- PAO1 wild-type strain grown in NY+NO3 media, replicate 2; GSM711455- PAO1 wild-type strain grown in NY+NO3 media, replicate 3.

### Metabolic Profile Analysis

Phenotype Microarray (Biolog; Hayward, CA) PM1 and PM2 (carbon), PM3B (nitrogen), and PM4A (sulfur and phosphorus) plates were employed to assess the ability of the strains to utilize various sources of carbon, nitrogen, sulfur, and phosphorus. Experiments were performed according to the manufacturer’s instructions. Provided minimal media was used to grow overnight cultures of WT and Δ*PA1006* PAO1 and these were used to inoculate the 96-well plates provided by Biolog. These were incubated without shaking at 37°C and growth was monitored by optical density of the tetrazolium dye at 590 nm at 24 and 48 h in a SpectraMax M5 plate reader (Molecular Devices; MDS Analytical; Toronto, ON).

### *Pseudomonas* Quinolone Signal (PQS) Analysis

PQS was assayed as previously described [54]. Briefly, bacteria grown aerobically overnight in NY or NY supplemented with 100 mM KNO_3_ were extracted twice with 0.001% glacial acetic acid-acidified ethyl acetate. Extracts were evaporated and re-suspended in ethyl acetate:acetonitrile**.** Samples were separated by Thin Layer Chromatography on silica gel plates and visualized using a hand-held UV lamp [54].

## Supporting Information

File S1 **Table showing that** ***PA1006*** **is not required for anaerobic growth with nitrite or arginine.**
(PDF)

File S2 ***PA1006*** **is not required for biofilm formation in a static dish system.** Biofilms were grown and analyzed as indicated in methods. A) Representative confocal images of *Pae* strains expressing GFP in flow-cell biofilms. Images were taken at random locations of each flow cell using confocal laser scanning microscope. B) COMSTAT analysis of biofilms.
(PDF)

File S3 **Confirmation of altered** ***rhlA, rhlI*** **, and** ***rhlR*** **gene expression in the Δ** ***PA1006*** **mutant to WT when grown in the presence of nitrate.** β-galactosidase -promoter fusion reporter constructs were used to determine expression levels.
(PDF)

File S4 **Microarray analysis of gene expression comparing Δ** ***PA1006*** **mutant to WT PAO1 in the absence of nitrate (worksheet 1).** This shows SAM analysis of microarray data of gene expression comparing Δ*PA1006* mutant to WT PAO1 in the absence of nitrate (worksheet 2).
(XLSX)

File S5 **Microarray analysis of gene expression comparing Δ** ***PA1006*** **mutant to WT PAO1 in the presence of nitrate (worksheet 1).** This shows SAM analysis of microarray data of gene expression comparing Δ*PA1006* mutant to WT PAO1 in the presence of nitrate (worksheet 2).
(XLS)

File S6 **Iron-regulated genes (with putative fur-boxes) show altered expression in the Δ** ***PA1006*** **mutant compared to WT PAO1.**
(XLSX)

File S7 **Phenotype Microarray™ analysis (Biolog) comparing metabolic profiles of Δ** ***PA1006*** **mutant to WT PAO1 in the absence of nitrate (excel spreadsheet).**
(XLSX)

File S8 **KEGG Pathway analysis results in Excel Spreadsheet format.**
(XLSX)

File S9 **KEGG Pathway analysis results in PowerPoint file format.**
(PPT)
