# Supplementary material for: Pseudomonas aeruginosa PA1006, Which Plays a Role in Molybdenum Homeostasis, Is Required for Nitrate Utilization, Biofilm Formation, and Virulence
Source: PLoS One. 2013 Feb 8;8(2):e55594. doi: 10.1371/journal.pone.0055594 (PMC3568122; doi:10.1371/journal.pone.0055594)
Supplement: File S1 — Table showing that PA1006 is not required for anaerobic growth with nitrite or arginine. (PDF) [file pone.0055594.s001.pdf]

Supplemental File 1

Table. PA1006 is not required for anaerobic growth with nitrite or arginine.

	**Anaerobic**				**Aerobic[d]**		
**Strain**	**KNO_3[a]**	**KNO_2[b]**	**Arginine[c]**	**-**	**KNO_3**	**KNO_2**	**NH_4Cl**
**PAO1**	+	+	+	-	+	+	+
**ΔPA1006**	-	+	+	-	-	-	+
**ΔPA1006 (attb:PA1006)**	+	+	+	-	+	+	+

[a] Anaerobic growth on KNO_3 was determined by plating on LB plates containing 100 mM KNO_3 and incubating under anaerobic conditions for 48 hrs. The WT strain PAO1 grows on this media after 24 hrs.

[b]Anaerobic growth on KNO_2 was determined by plating strains on LB plates containing 10mM KNO_2 and incubating under anaerobic conditions for 72 hours. Growth of the WT strain PAO1 appears after 72 hours. (+), growth, (-), no growth.

[c]Anaerobic growth on arginine was determined by plating on LB plates containing 40mM L-Arginine and incubating under anaerobic conditions for 5 days. Growth of the WT strain PAO1 appears after 5 days. (+), growth, (-), no growth.

[d]Aerobic growth was determined by plating on VBMM plates containing either 100 mM KNO_3 , 10 mMKNO_2, or NH_4Cl, as the sole nitrogen source, and incubating under aerobic conditions for 48 hrs. (+), growth, (-), no growth.
