# Supplementary material for: Pseudomonas aeruginosa PA1006, Which Plays a Role in Molybdenum Homeostasis, Is Required for Nitrate Utilization, Biofilm Formation, and Virulence
Source: PLoS One. 2013 Feb 8;8(2):e55594. doi: 10.1371/journal.pone.0055594 (PMC3568122; doi:10.1371/journal.pone.0055594)
Supplement: File S2 — PA1006 is not required for biofilm formation in a static dish system. Biofilms were grown and analyzed as indicated in methods. A) Representative confocal images of Pae strains expressing GFP in flow-cell biofilms. Images were taken at random locations of each flow cell using confocal laser scanning microscope. B) COMSTAT analysis of biofilms. (PDF) [file pone.0055594.s002.pdf]

PAO1 | ΔPA1006 | ΔPA1006 (attb:PA1006)

A

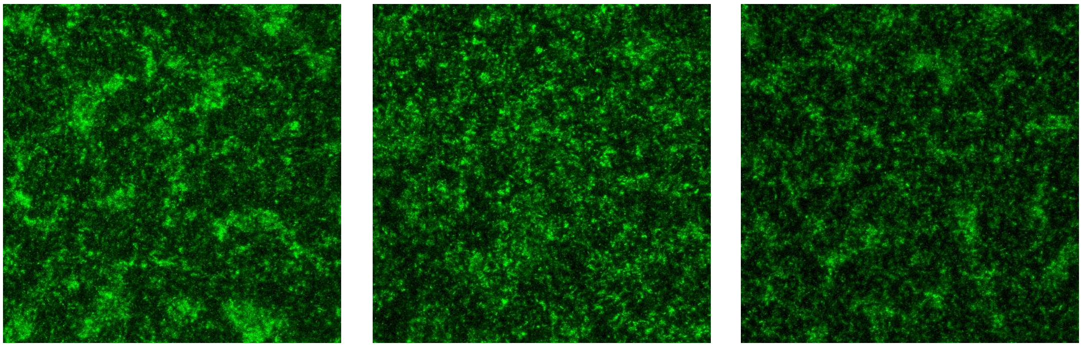

B

	Biomass (mm^3/mm^2)	Average Thickness (μm)
**PAO1**	3.97 ± 0.43	9.31 ± 0.55
**ΔPA1006**	3.82 ± 1.30	9.41 ± 1.95
**ΔPA1006 (attb:PA1006)**	4.43 ± 0.45	11.52 ± 0.96
