# Supplementary material for: Pseudomonas aeruginosa PA1006, Which Plays a Role in Molybdenum Homeostasis, Is Required for Nitrate Utilization, Biofilm Formation, and Virulence
Source: PLoS One. 2013 Feb 8;8(2):e55594. doi: 10.1371/journal.pone.0055594 (PMC3568122; doi:10.1371/journal.pone.0055594)
Supplement: File S3 — Confirmation of altered rhlA, rhlI , and rhlR gene expression in the Δ PA1006 mutant to WT when grown in the presence of nitrate. β-galactosidase -promoter fusion reporter constructs were used to determine expression levels. (PDF) [file pone.0055594.s003.pdf]

Supplemental File 3: Confirmation of altered rhlA, rhlI, and rhlR gene expression in the Δ*PA1006* mutant to WT PAO1 when grown in the presence of nitrate. β-galactosidase rhl-promoter fusion reporter constructs were used to determine expression levels.

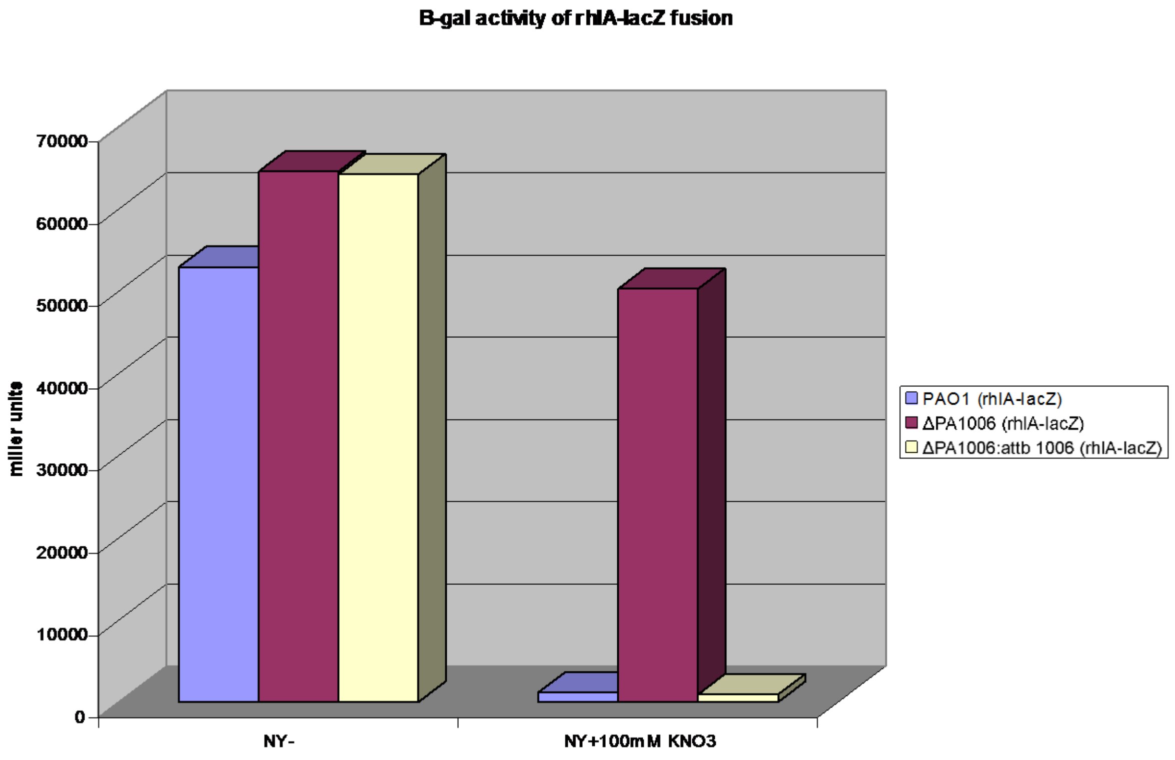

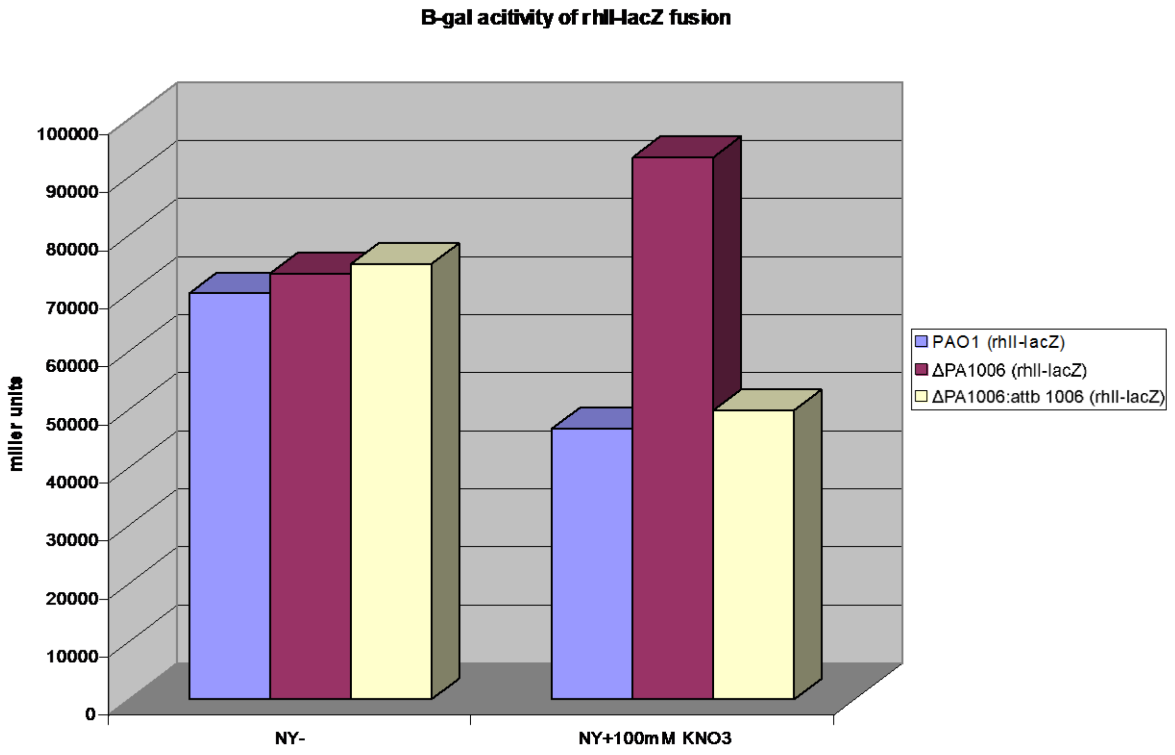

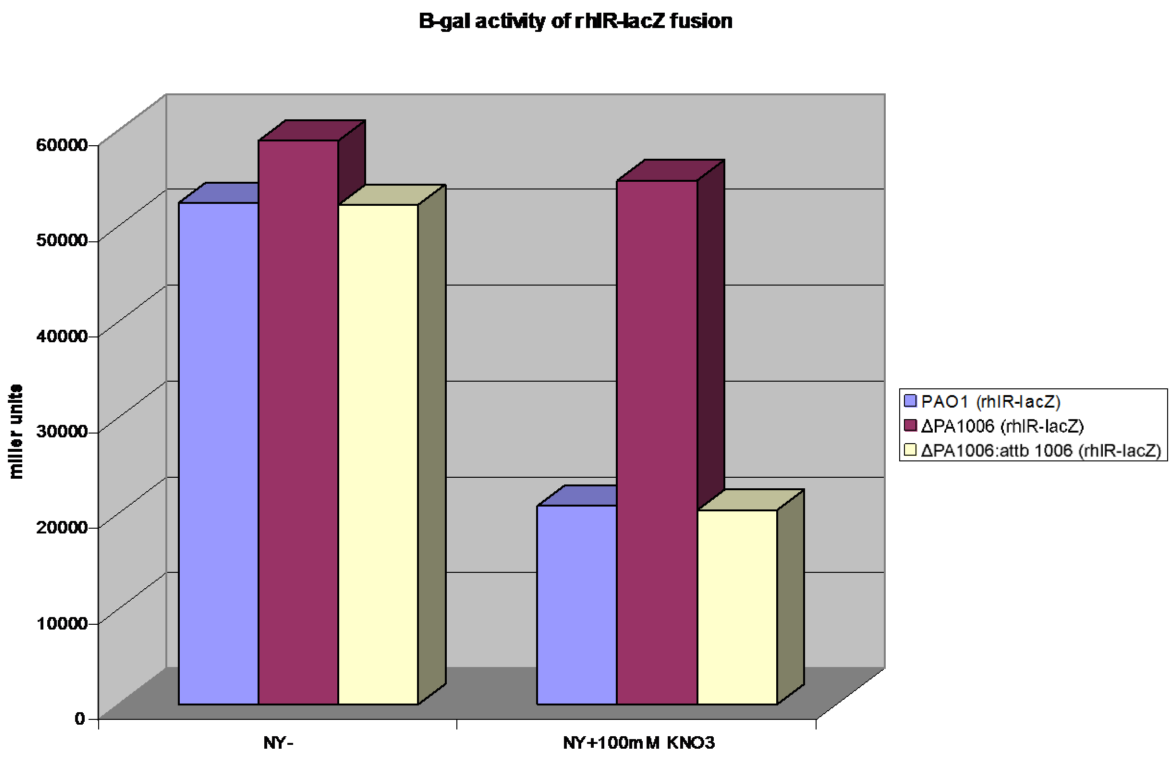

Supplemental method for transcriptional fusion assays. β-galactosidase activity was determined at 30°C as described previously [90]. Plasmids containing the *rhlA'-lacZ* [48], *rhlI'-lacZ* (Van Delden C), and *rhlR'-lacZ*, [48] were introduced into *Pae*WT strain, Δ*nbvF* mutant, and complemented Δ*nbvF* mutant by electroporation and transformants were selected by growth on PTSB with 200 µg/ml carbenicillin ($PTSB^{carb}$). Overnight cultures (16 h) were grown in $PTSB^{carb}$ at 37°C and then diluted to OD_{600} of 0.05 in $PTSB^{carb}$. Cells were collected at OD_{660} ~1.2 and assayed β-galactosidase activity as described [90]. Data presented are representative of at least two independent experiments.
