# Supplementary material for: Pseudomonas aeruginosa PA1006, Which Plays a Role in Molybdenum Homeostasis, Is Required for Nitrate Utilization, Biofilm Formation, and Virulence
Source: PLoS One. 2013 Feb 8;8(2):e55594. doi: 10.1371/journal.pone.0055594 (PMC3568122; doi:10.1371/journal.pone.0055594)
Supplement: File S9 — KEGG Pathway analysis results in PowerPoint file format. (PPT) [file pone.0055594.s009.ppt]

## Slide 1
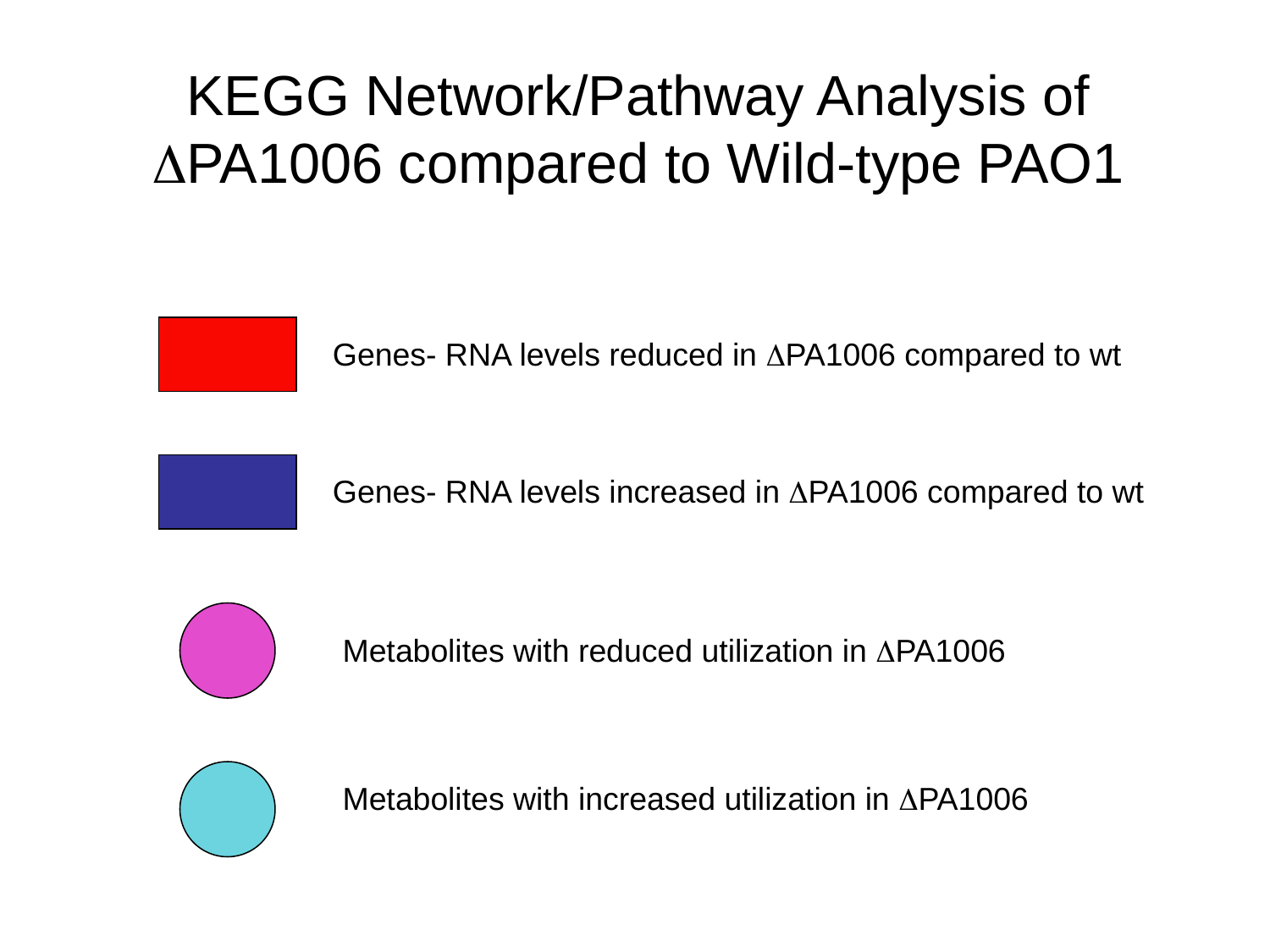

KEGG Network/Pathway Analysis of
ΔPA1006 compared to Wild-type PAO1
Genes- RNA levels reduced in ΔPA1006 compared to wt
Genes- RNA levels increased in ΔPA1006 compared to wt
Metabolites with reduced utilization in ΔPA1006
Metabolites with increased utilization in ΔPA1006

## Slide 2
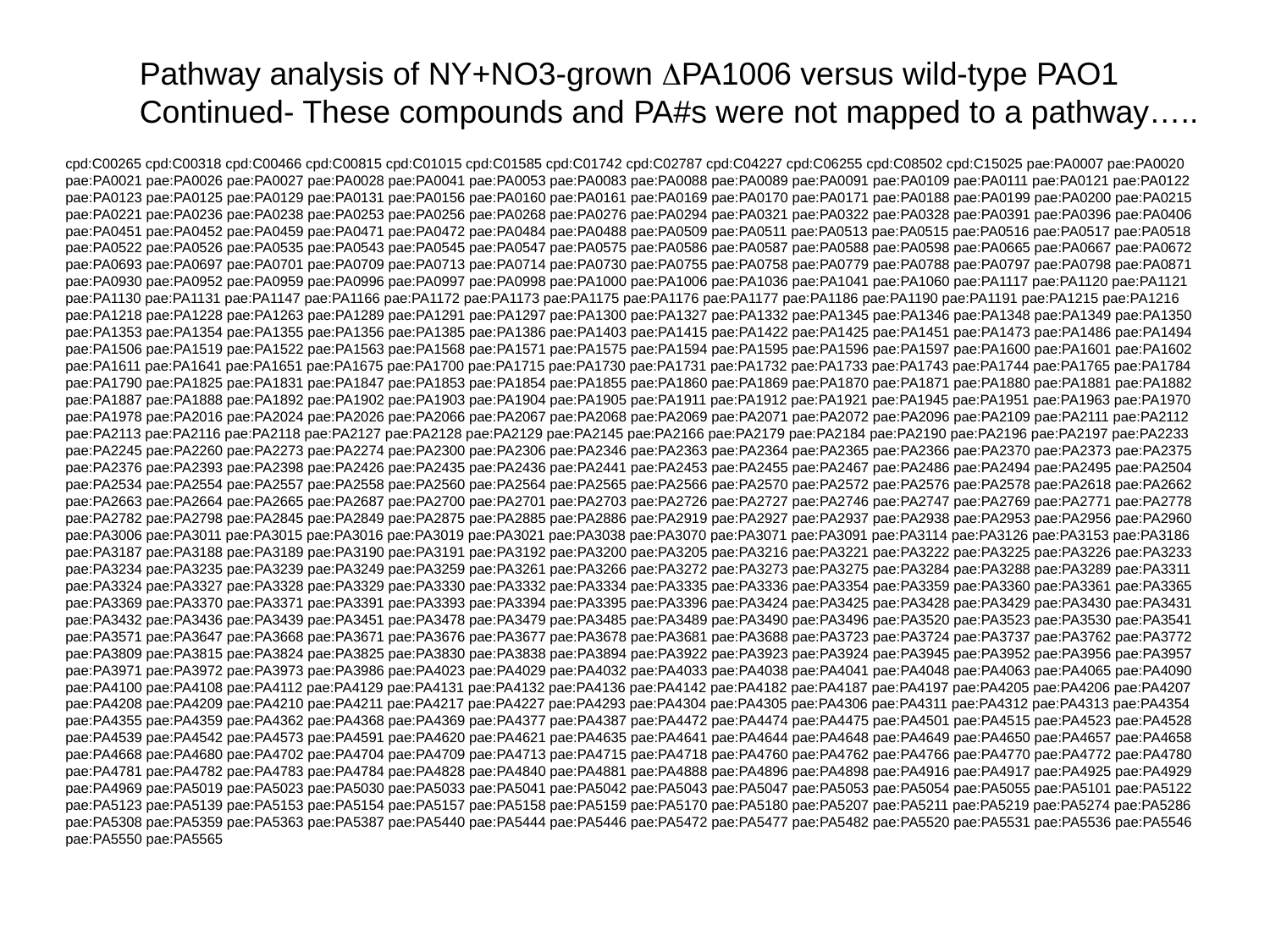

Pathway analysis of NY+NO3-grown ΔPA1006 versus wild-type PAO1
Continued- These compounds and PA#s were not mapped to a pathway…..
cpd:C00265 cpd:C00318 cpd:C00466 cpd:C00815 cpd:C01015 cpd:C01585 cpd:C01742 cpd:C02787 cpd:C04227 cpd:C06255 cpd:C08502 cpd:C15025 pae:PA0007 pae:PA0020 pae:PA0021 pae:PA0026 pae:PA0027 pae:PA0028 pae:PA0041 pae:PA0053 pae:PA0083 pae:PA0088 pae:PA0089 pae:PA0091 pae:PA0109 pae:PA0111 pae:PA0121 pae:PA0122 pae:PA0123 pae:PA0125 pae:PA0129 pae:PA0131 pae:PA0156 pae:PA0160 pae:PA0161 pae:PA0169 pae:PA0170 pae:PA0171 pae:PA0188 pae:PA0199 pae:PA0200 pae:PA0215 pae:PA0221 pae:PA0236 pae:PA0238 pae:PA0253 pae:PA0256 pae:PA0268 pae:PA0276 pae:PA0294 pae:PA0321 pae:PA0322 pae:PA0328 pae:PA0391 pae:PA0396 pae:PA0406 pae:PA0451 pae:PA0452 pae:PA0459 pae:PA0471 pae:PA0472 pae:PA0484 pae:PA0488 pae:PA0509 pae:PA0511 pae:PA0513 pae:PA0515 pae:PA0516 pae:PA0517 pae:PA0518 pae:PA0522 pae:PA0526 pae:PA0535 pae:PA0543 pae:PA0545 pae:PA0547 pae:PA0575 pae:PA0586 pae:PA0587 pae:PA0588 pae:PA0598 pae:PA0665 pae:PA0667 pae:PA0672 pae:PA0693 pae:PA0697 pae:PA0701 pae:PA0709 pae:PA0713 pae:PA0714 pae:PA0730 pae:PA0755 pae:PA0758 pae:PA0779 pae:PA0788 pae:PA0797 pae:PA0798 pae:PA0871 pae:PA0930 pae:PA0952 pae:PA0959 pae:PA0996 pae:PA0997 pae:PA0998 pae:PA1000 pae:PA1006 pae:PA1036 pae:PA1041 pae:PA1060 pae:PA1117 pae:PA1120 pae:PA1121 pae:PA1130 pae:PA1131 pae:PA1147 pae:PA1166 pae:PA1172 pae:PA1173 pae:PA1175 pae:PA1176 pae:PA1177 pae:PA1186 pae:PA1190 pae:PA1191 pae:PA1215 pae:PA1216 pae:PA1218 pae:PA1228 pae:PA1263 pae:PA1289 pae:PA1291 pae:PA1297 pae:PA1300 pae:PA1327 pae:PA1332 pae:PA1345 pae:PA1346 pae:PA1348 pae:PA1349 pae:PA1350 pae:PA1353 pae:PA1354 pae:PA1355 pae:PA1356 pae:PA1385 pae:PA1386 pae:PA1403 pae:PA1415 pae:PA1422 pae:PA1425 pae:PA1451 pae:PA1473 pae:PA1486 pae:PA1494 pae:PA1506 pae:PA1519 pae:PA1522 pae:PA1563 pae:PA1568 pae:PA1571 pae:PA1575 pae:PA1594 pae:PA1595 pae:PA1596 pae:PA1597 pae:PA1600 pae:PA1601 pae:PA1602 pae:PA1611 pae:PA1641 pae:PA1651 pae:PA1675 pae:PA1700 pae:PA1715 pae:PA1730 pae:PA1731 pae:PA1732 pae:PA1733 pae:PA1743 pae:PA1744 pae:PA1765 pae:PA1784 pae:PA1790 pae:PA1825 pae:PA1831 pae:PA1847 pae:PA1853 pae:PA1854 pae:PA1855 pae:PA1860 pae:PA1869 pae:PA1870 pae:PA1871 pae:PA1880 pae:PA1881 pae:PA1882 pae:PA1887 pae:PA1888 pae:PA1892 pae:PA1902 pae:PA1903 pae:PA1904 pae:PA1905 pae:PA1911 pae:PA1912 pae:PA1921 pae:PA1945 pae:PA1951 pae:PA1963 pae:PA1970 pae:PA1978 pae:PA2016 pae:PA2024 pae:PA2026 pae:PA2066 pae:PA2067 pae:PA2068 pae:PA2069 pae:PA2071 pae:PA2072 pae:PA2096 pae:PA2109 pae:PA2111 pae:PA2112 pae:PA2113 pae:PA2116 pae:PA2118 pae:PA2127 pae:PA2128 pae:PA2129 pae:PA2145 pae:PA2166 pae:PA2179 pae:PA2184 pae:PA2190 pae:PA2196 pae:PA2197 pae:PA2233 pae:PA2245 pae:PA2260 pae:PA2273 pae:PA2274 pae:PA2300 pae:PA2306 pae:PA2346 pae:PA2363 pae:PA2364 pae:PA2365 pae:PA2366 pae:PA2370 pae:PA2373 pae:PA2375 pae:PA2376 pae:PA2393 pae:PA2398 pae:PA2426 pae:PA2435 pae:PA2436 pae:PA2441 pae:PA2453 pae:PA2455 pae:PA2467 pae:PA2486 pae:PA2494 pae:PA2495 pae:PA2504 pae:PA2534 pae:PA2554 pae:PA2557 pae:PA2558 pae:PA2560 pae:PA2564 pae:PA2565 pae:PA2566 pae:PA2570 pae:PA2572 pae:PA2576 pae:PA2578 pae:PA2618 pae:PA2662 pae:PA2663 pae:PA2664 pae:PA2665 pae:PA2687 pae:PA2700 pae:PA2701 pae:PA2703 pae:PA2726 pae:PA2727 pae:PA2746 pae:PA2747 pae:PA2769 pae:PA2771 pae:PA2778 pae:PA2782 pae:PA2798 pae:PA2845 pae:PA2849 pae:PA2875 pae:PA2885 pae:PA2886 pae:PA2919 pae:PA2927 pae:PA2937 pae:PA2938 pae:PA2953 pae:PA2956 pae:PA2960 pae:PA3006 pae:PA3011 pae:PA3015 pae:PA3016 pae:PA3019 pae:PA3021 pae:PA3038 pae:PA3070 pae:PA3071 pae:PA3091 pae:PA3114 pae:PA3126 pae:PA3153 pae:PA3186 pae:PA3187 pae:PA3188 pae:PA3189 pae:PA3190 pae:PA3191 pae:PA3192 pae:PA3200 pae:PA3205 pae:PA3216 pae:PA3221 pae:PA3222 pae:PA3225 pae:PA3226 pae:PA3233 pae:PA3234 pae:PA3235 pae:PA3239 pae:PA3249 pae:PA3259 pae:PA3261 pae:PA3266 pae:PA3272 pae:PA3273 pae:PA3275 pae:PA3284 pae:PA3288 pae:PA3289 pae:PA3311 pae:PA3324 pae:PA3327 pae:PA3328 pae:PA3329 pae:PA3330 pae:PA3332 pae:PA3334 pae:PA3335 pae:PA3336 pae:PA3354 pae:PA3359 pae:PA3360 pae:PA3361 pae:PA3365 pae:PA3369 pae:PA3370 pae:PA3371 pae:PA3391 pae:PA3393 pae:PA3394 pae:PA3395 pae:PA3396 pae:PA3424 pae:PA3425 pae:PA3428 pae:PA3429 pae:PA3430 pae:PA3431 pae:PA3432 pae:PA3436 pae:PA3439 pae:PA3451 pae:PA3478 pae:PA3479 pae:PA3485 pae:PA3489 pae:PA3490 pae:PA3496 pae:PA3520 pae:PA3523 pae:PA3530 pae:PA3541 pae:PA3571 pae:PA3647 pae:PA3668 pae:PA3671 pae:PA3676 pae:PA3677 pae:PA3678 pae:PA3681 pae:PA3688 pae:PA3723 pae:PA3724 pae:PA3737 pae:PA3762 pae:PA3772 pae:PA3809 pae:PA3815 pae:PA3824 pae:PA3825 pae:PA3830 pae:PA3838 pae:PA3894 pae:PA3922 pae:PA3923 pae:PA3924 pae:PA3945 pae:PA3952 pae:PA3956 pae:PA3957 pae:PA3971 pae:PA3972 pae:PA3973 pae:PA3986 pae:PA4023 pae:PA4029 pae:PA4032 pae:PA4033 pae:PA4038 pae:PA4041 pae:PA4048 pae:PA4063 pae:PA4065 pae:PA4090 pae:PA4100 pae:PA4108 pae:PA4112 pae:PA4129 pae:PA4131 pae:PA4132 pae:PA4136 pae:PA4142 pae:PA4182 pae:PA4187 pae:PA4197 pae:PA4205 pae:PA4206 pae:PA4207 pae:PA4208 pae:PA4209 pae:PA4210 pae:PA4211 pae:PA4217 pae:PA4227 pae:PA4293 pae:PA4304 pae:PA4305 pae:PA4306 pae:PA4311 pae:PA4312 pae:PA4313 pae:PA4354 pae:PA4355 pae:PA4359 pae:PA4362 pae:PA4368 pae:PA4369 pae:PA4377 pae:PA4387 pae:PA4472 pae:PA4474 pae:PA4475 pae:PA4501 pae:PA4515 pae:PA4523 pae:PA4528 pae:PA4539 pae:PA4542 pae:PA4573 pae:PA4591 pae:PA4620 pae:PA4621 pae:PA4635 pae:PA4641 pae:PA4644 pae:PA4648 pae:PA4649 pae:PA4650 pae:PA4657 pae:PA4658 pae:PA4668 pae:PA4680 pae:PA4702 pae:PA4704 pae:PA4709 pae:PA4713 pae:PA4715 pae:PA4718 pae:PA4760 pae:PA4762 pae:PA4766 pae:PA4770 pae:PA4772 pae:PA4780 pae:PA4781 pae:PA4782 pae:PA4783 pae:PA4784 pae:PA4828 pae:PA4840 pae:PA4881 pae:PA4888 pae:PA4896 pae:PA4898 pae:PA4916 pae:PA4917 pae:PA4925 pae:PA4929 pae:PA4969 pae:PA5019 pae:PA5023 pae:PA5030 pae:PA5033 pae:PA5041 pae:PA5042 pae:PA5043 pae:PA5047 pae:PA5053 pae:PA5054 pae:PA5055 pae:PA5101 pae:PA5122 pae:PA5123 pae:PA5139 pae:PA5153 pae:PA5154 pae:PA5157 pae:PA5158 pae:PA5159 pae:PA5170 pae:PA5180 pae:PA5207 pae:PA5211 pae:PA5219 pae:PA5274 pae:PA5286 pae:PA5308 pae:PA5359 pae:PA5363 pae:PA5387 pae:PA5440 pae:PA5444 pae:PA5446 pae:PA5472 pae:PA5477 pae:PA5482 pae:PA5520 pae:PA5531 pae:PA5536 pae:PA5546 pae:PA5550 pae:PA5565

## Slide 3
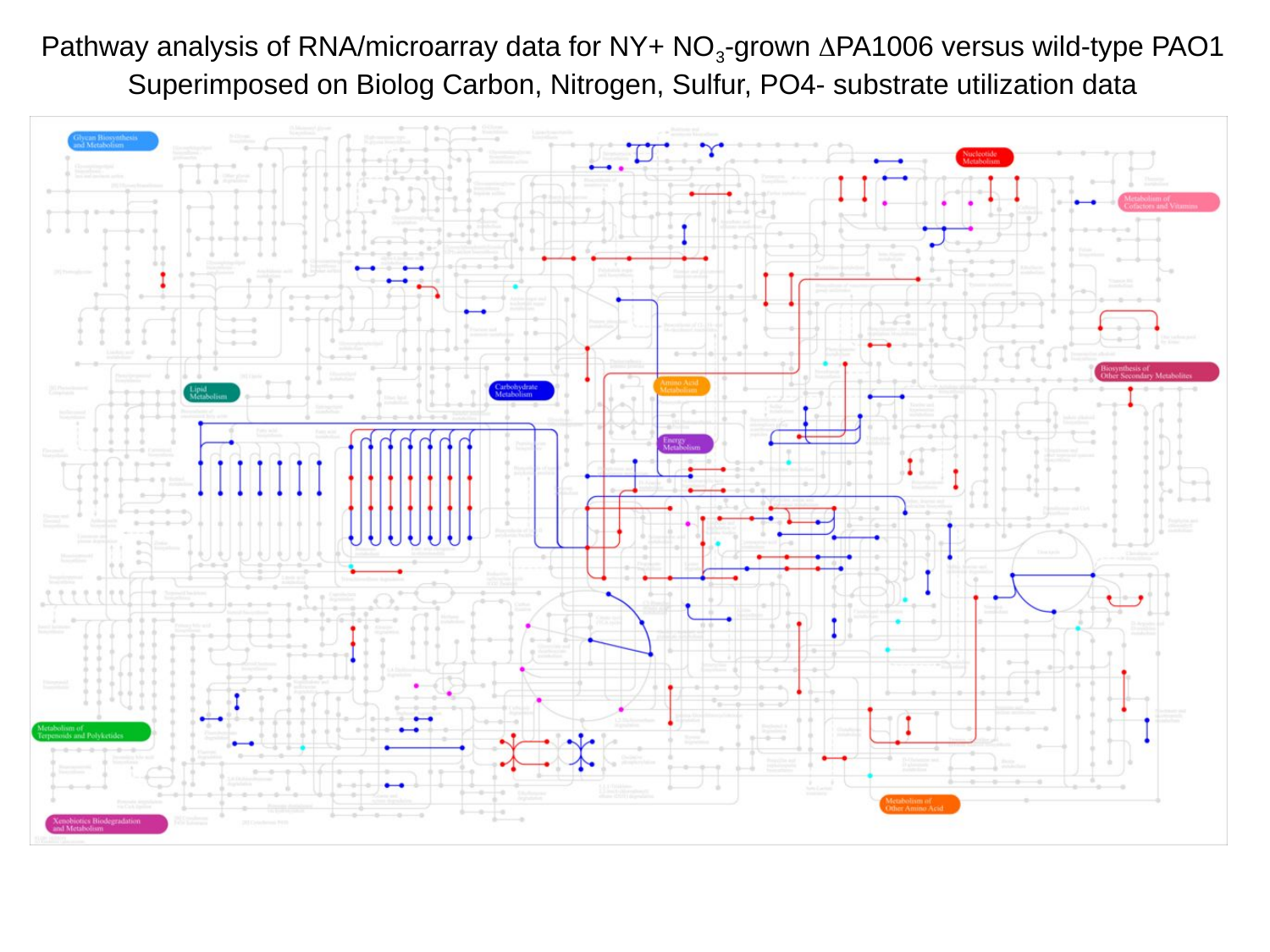

Pathway analysis of RNA/microarray data for NY+ NO3-grown ΔPA1006 versus wild-type PAO1
Superimposed on Biolog Carbon, Nitrogen, Sulfur, PO4- substrate utilization data

## Slide 4
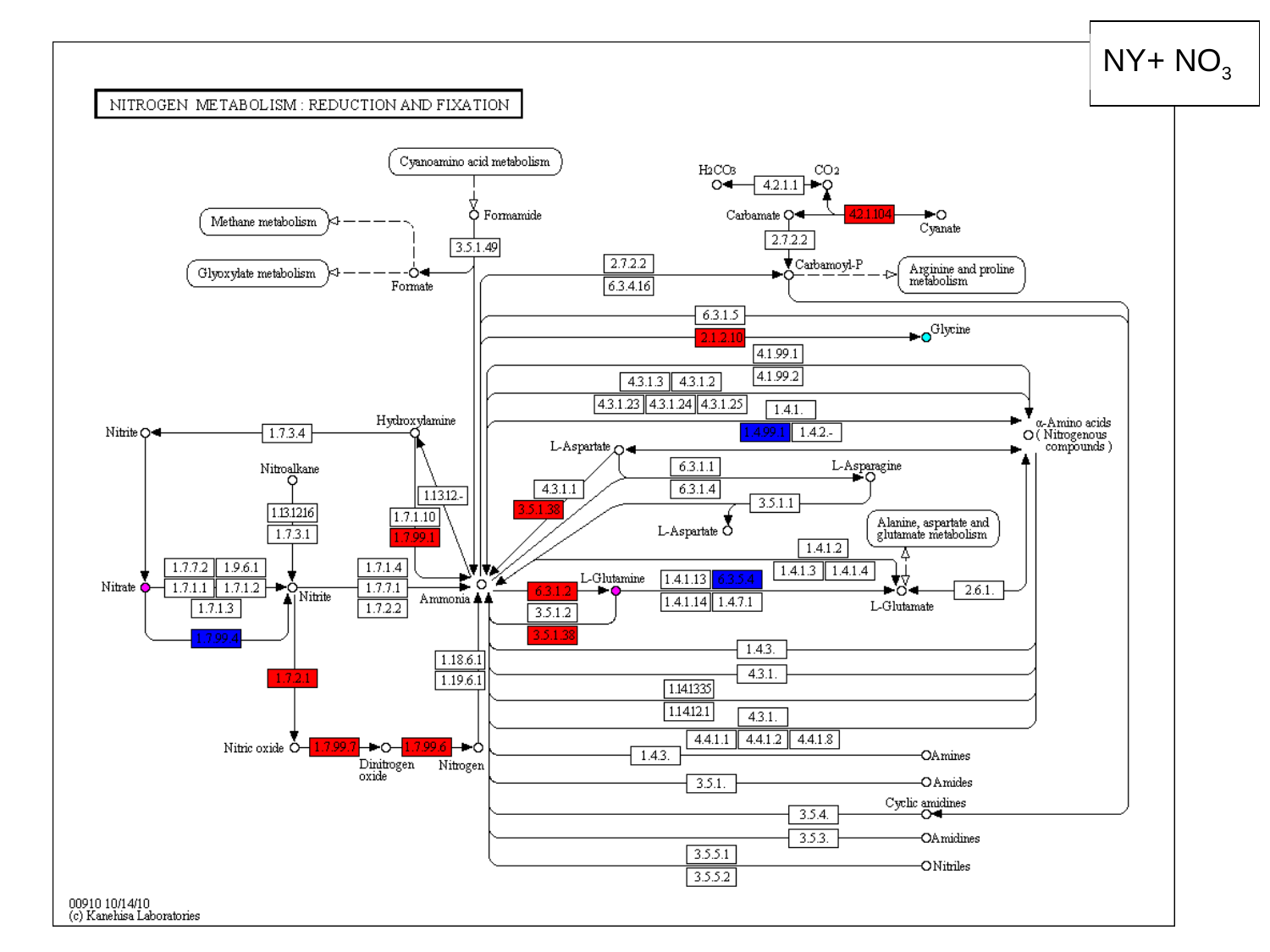

NY+ NO3

## Slide 5
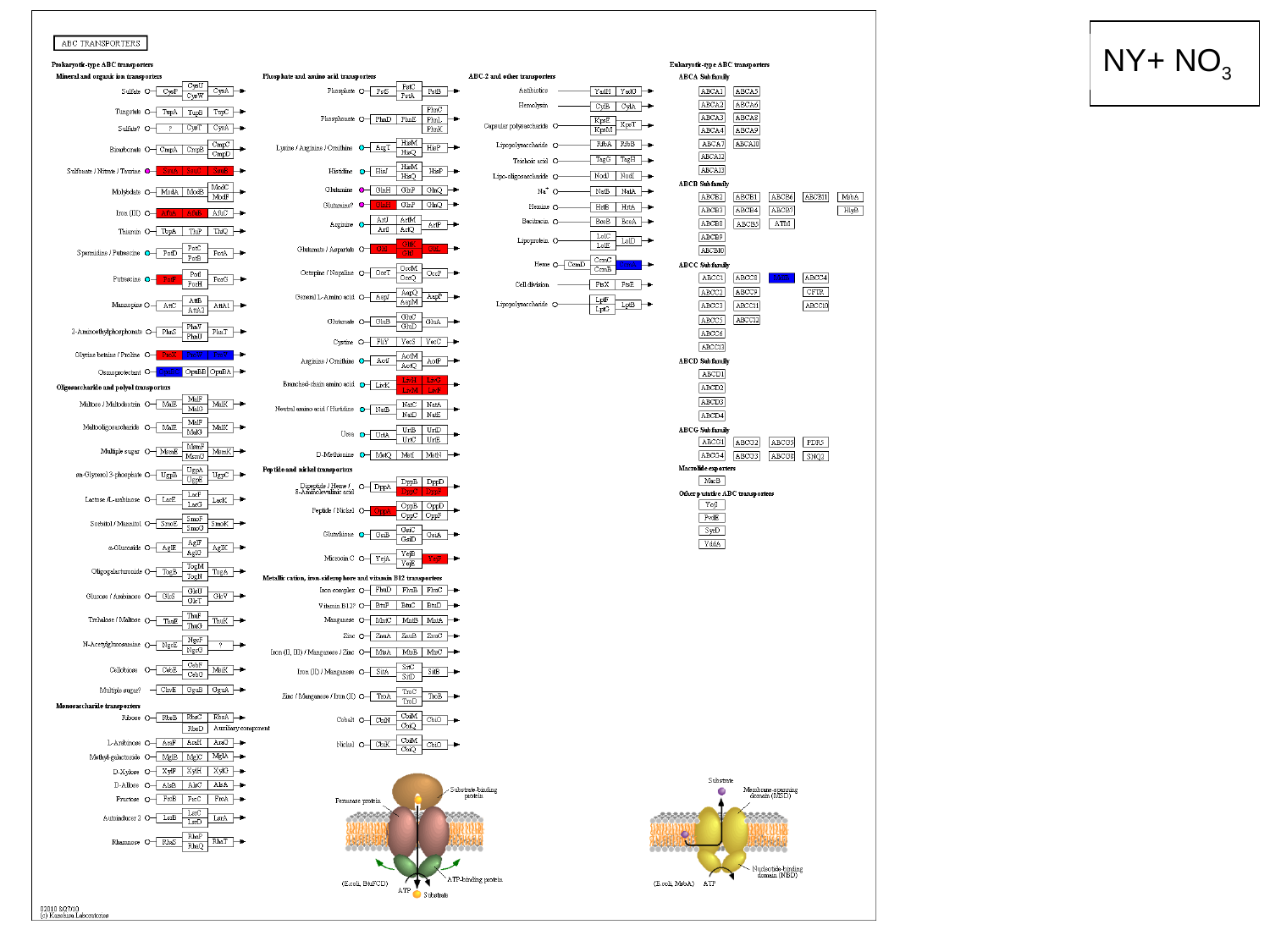

NY+ NO3

## Slide 6
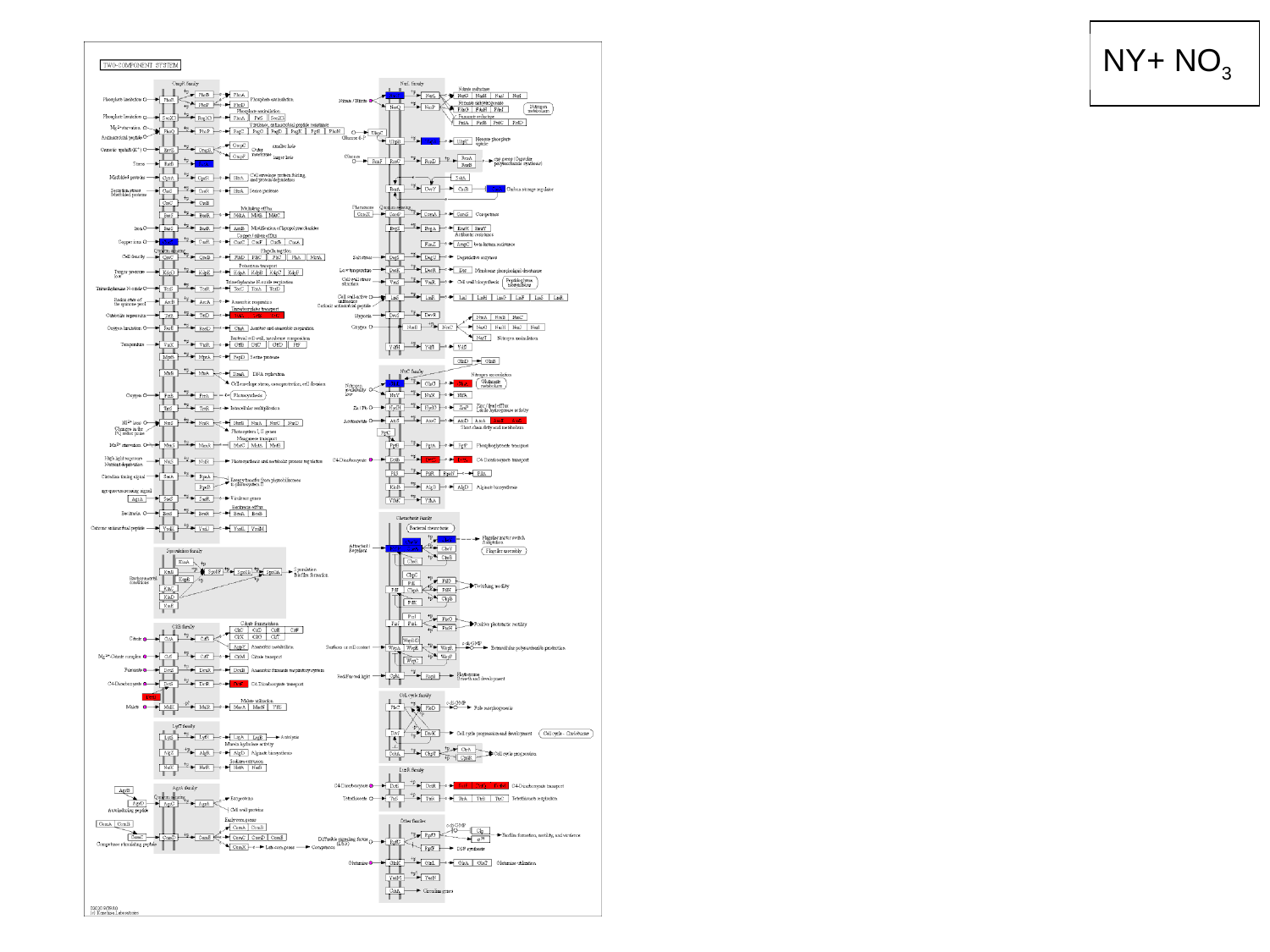

NY+ NO3

## Slide 7
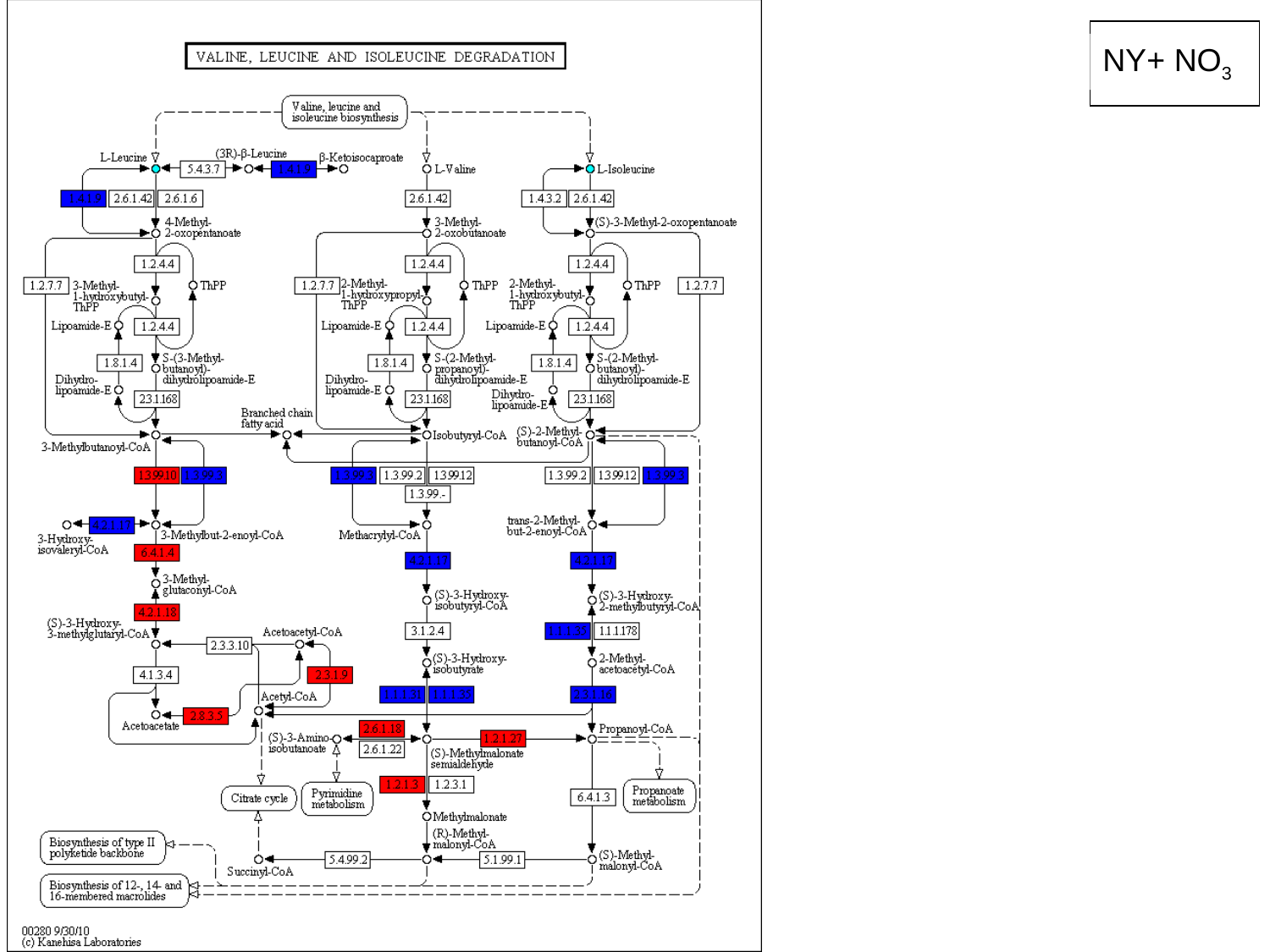

NY+ NO3

## Slide 8
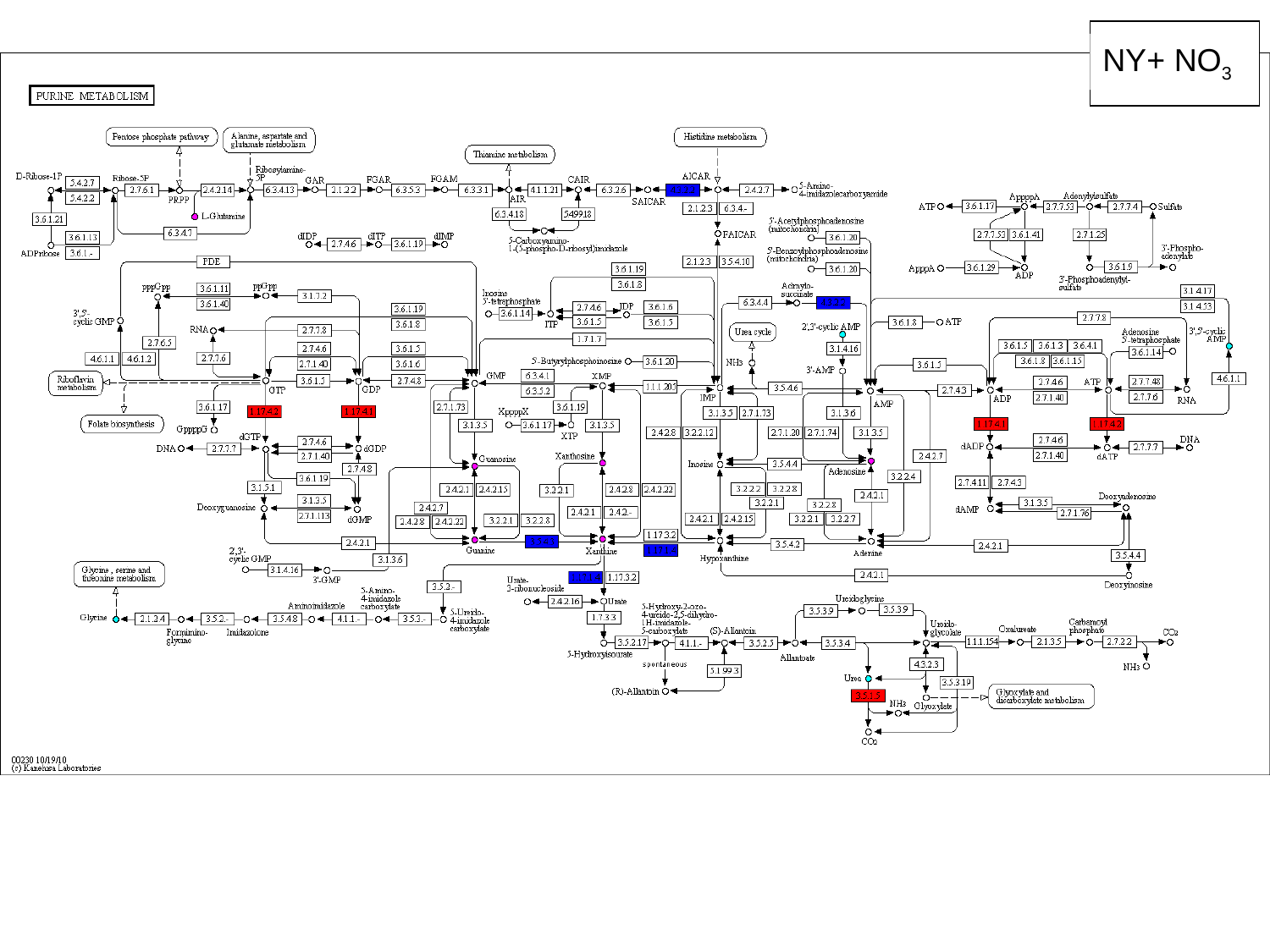

NY+ NO3

## Slide 9
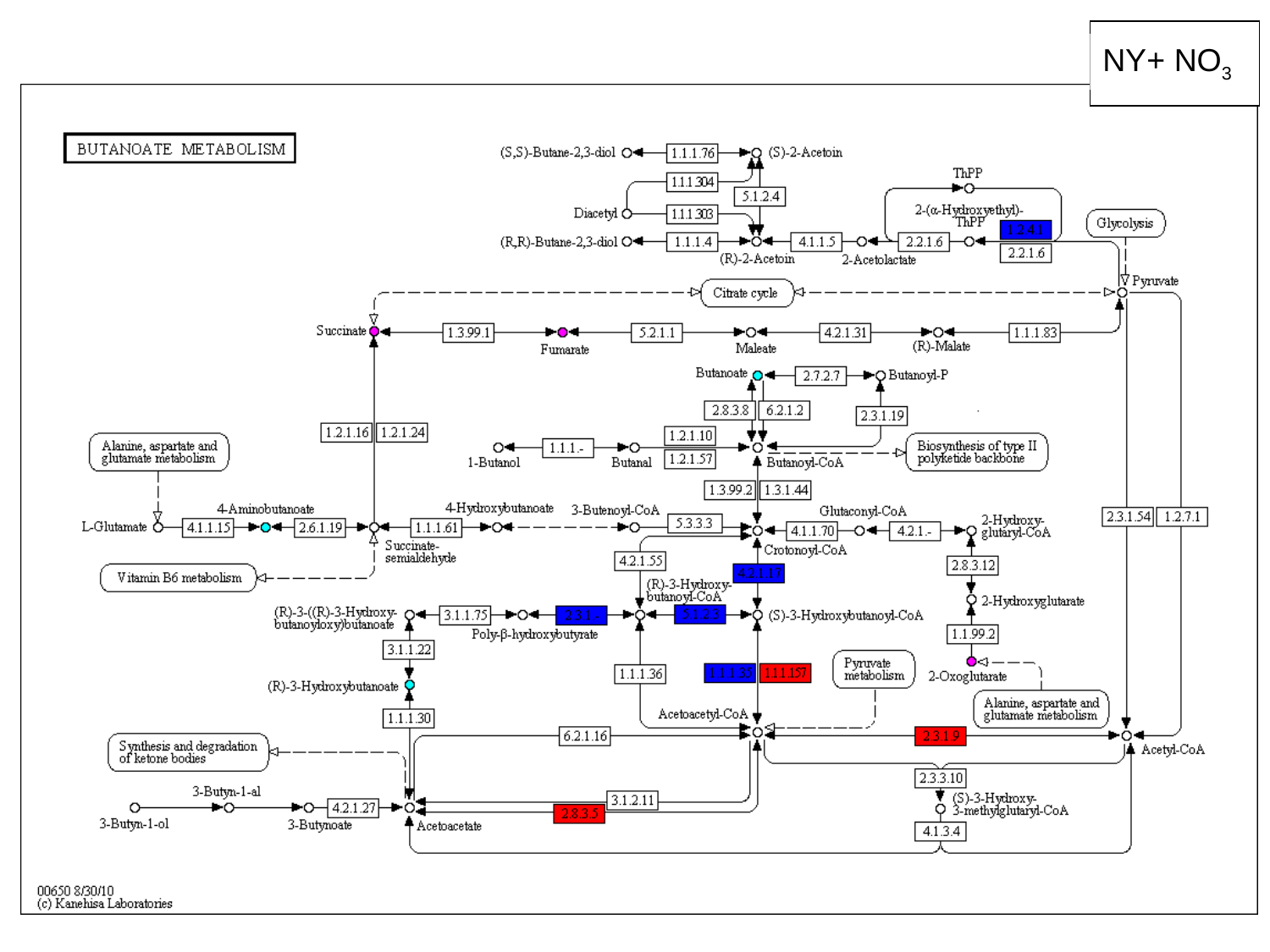

NY+ NO3

## Slide 10
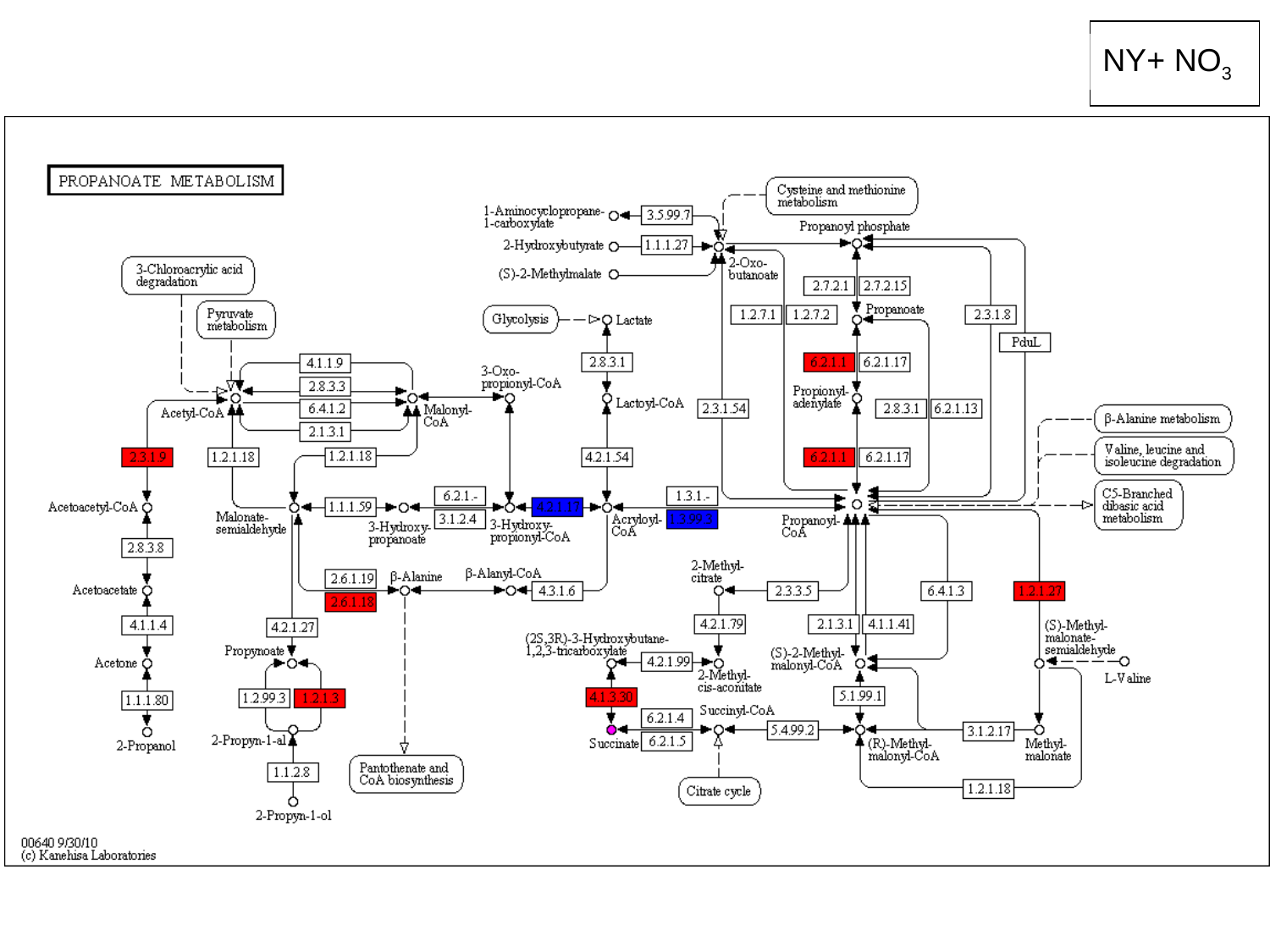

NY+ NO3

## Slide 11
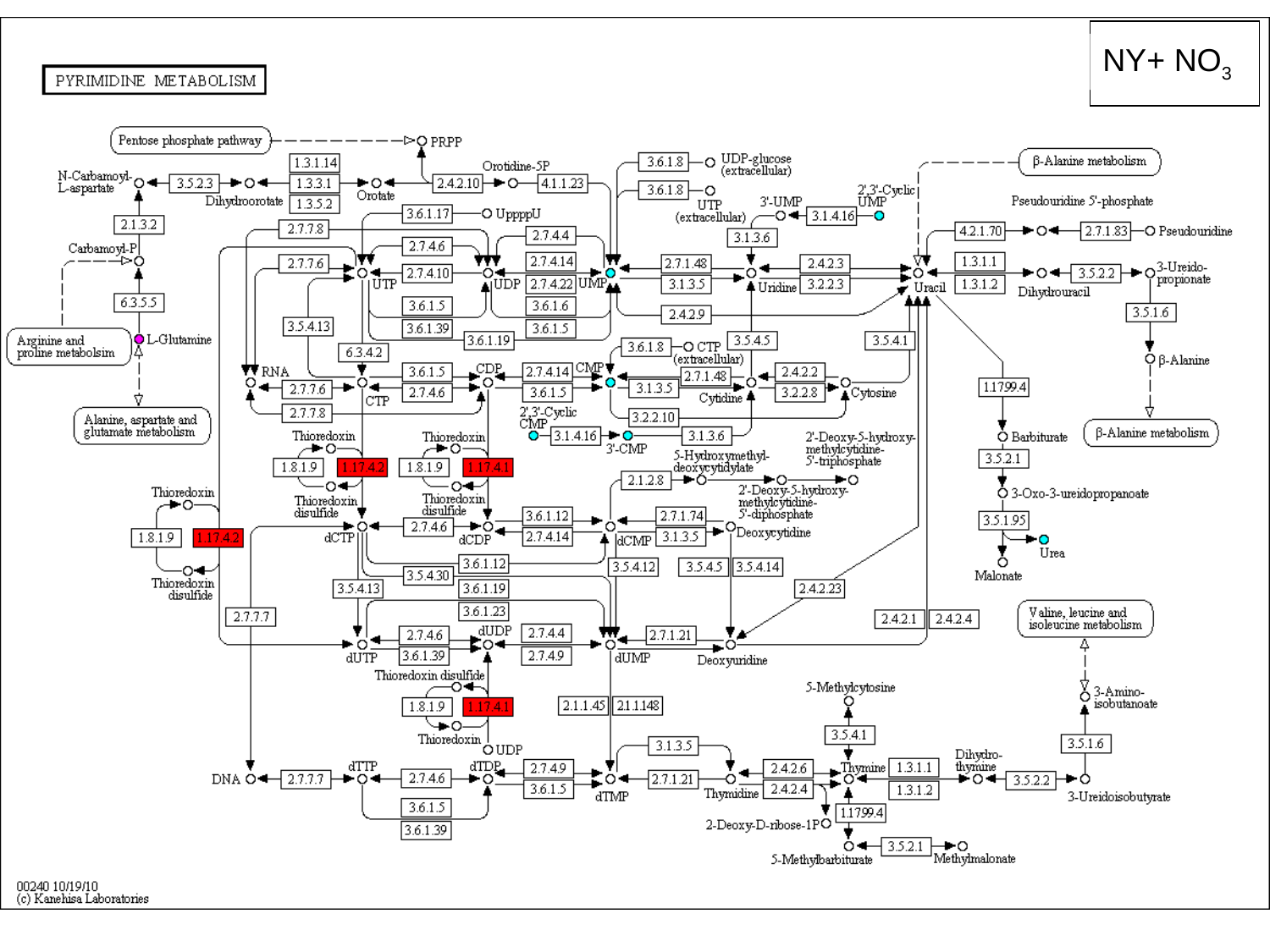

NY+ NO3

## Slide 12
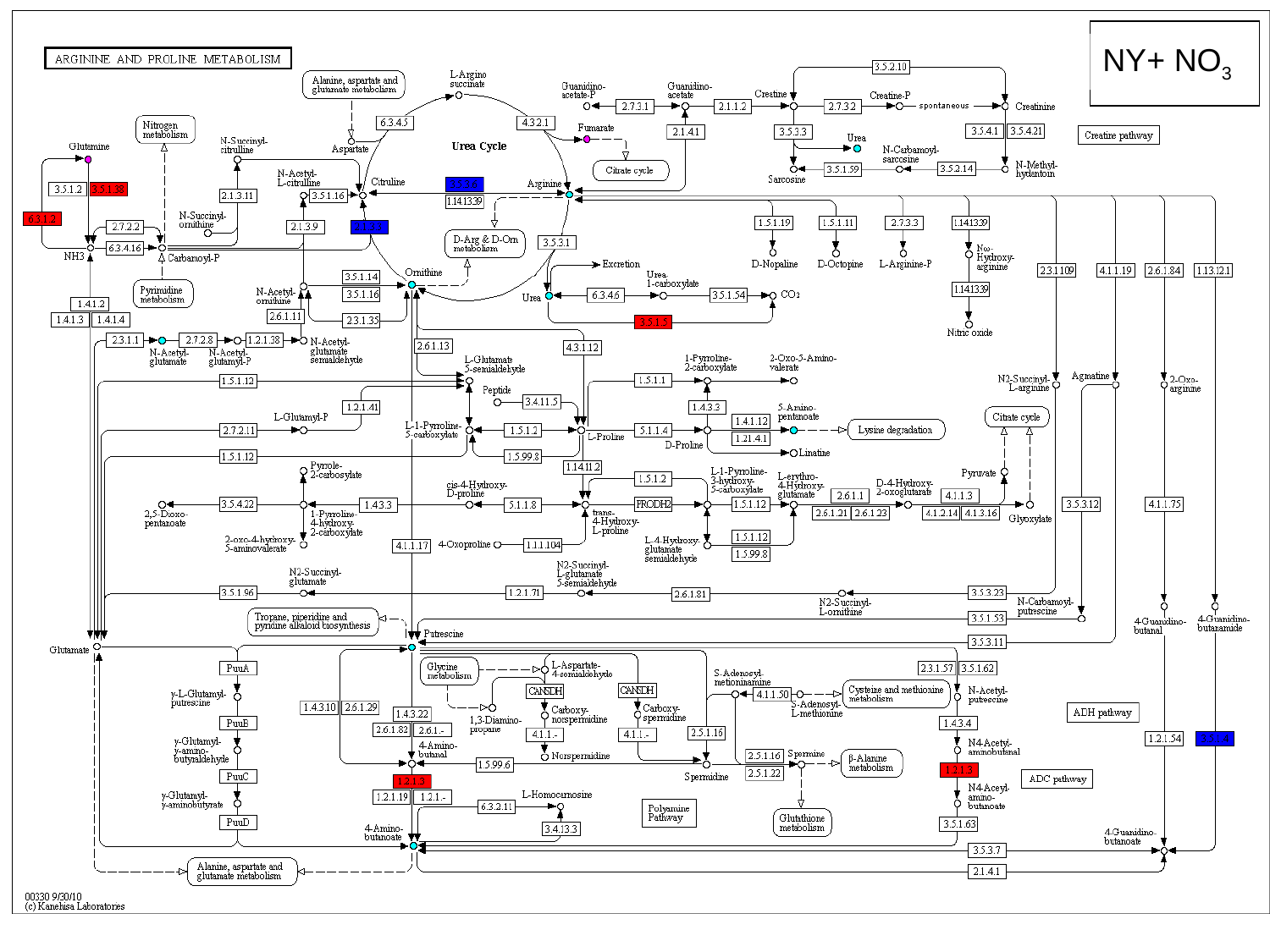

NY+ NO3

## Slide 13
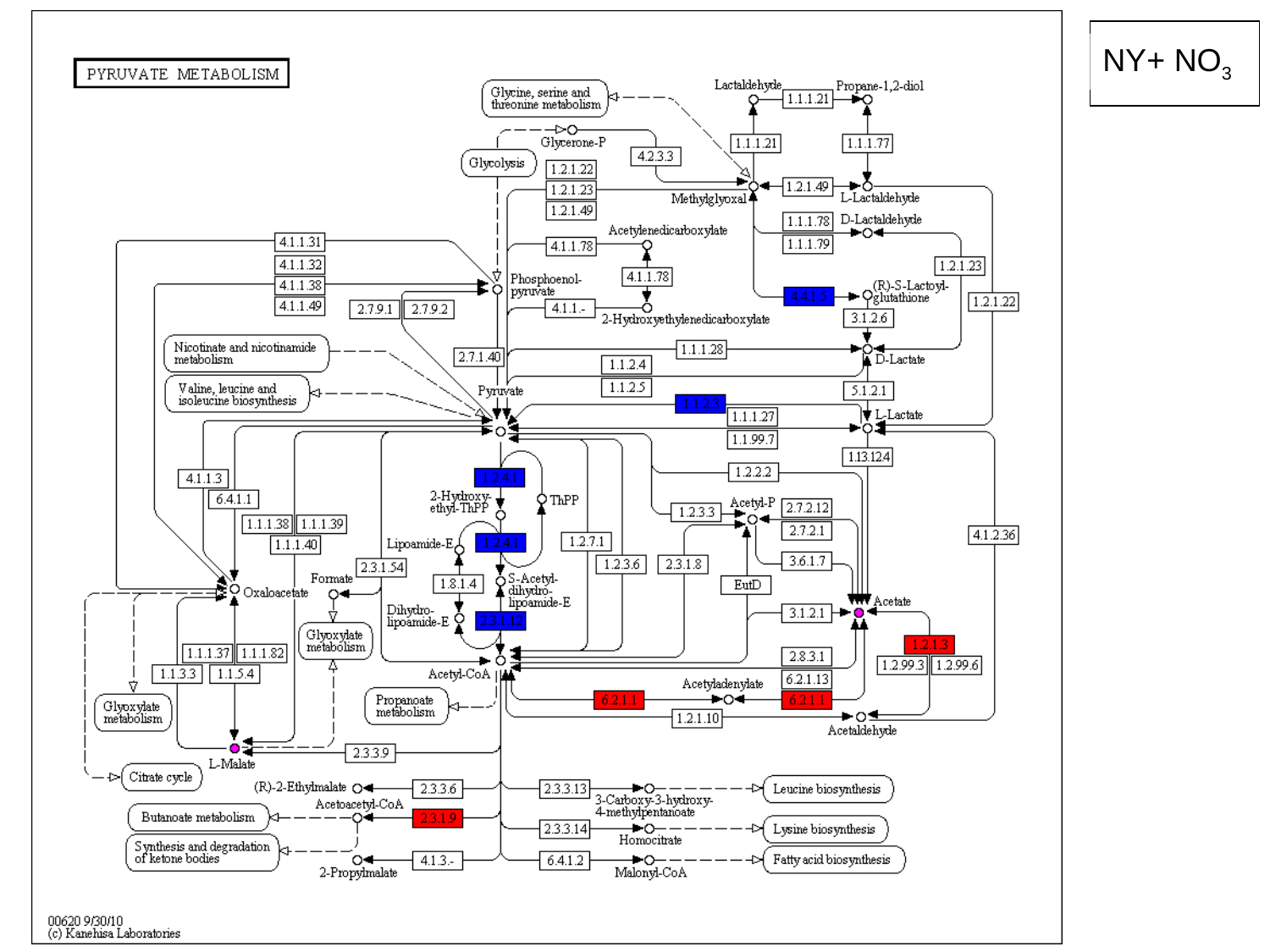

NY+ NO3

## Slide 14
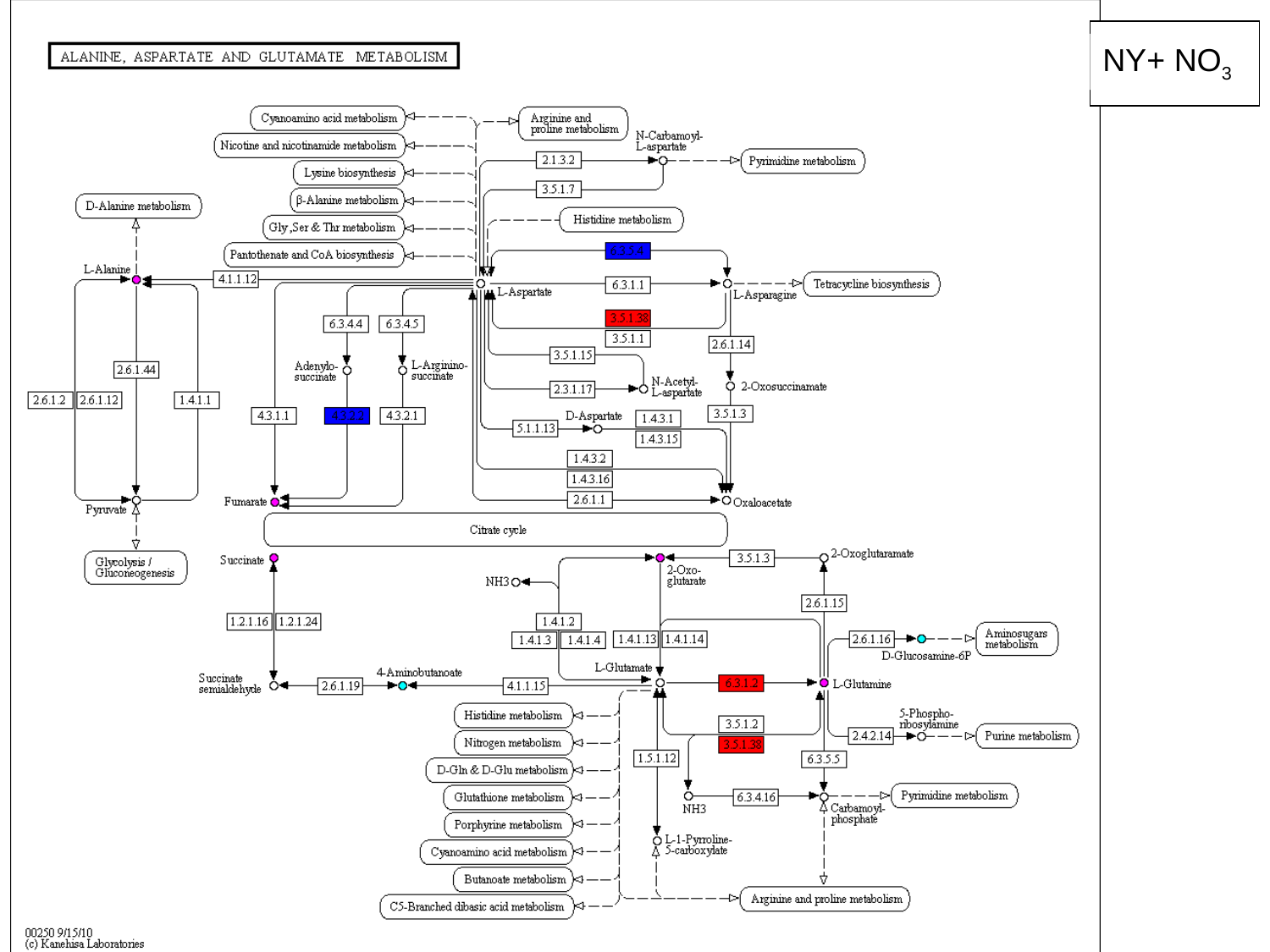

NY+ NO3

## Slide 15
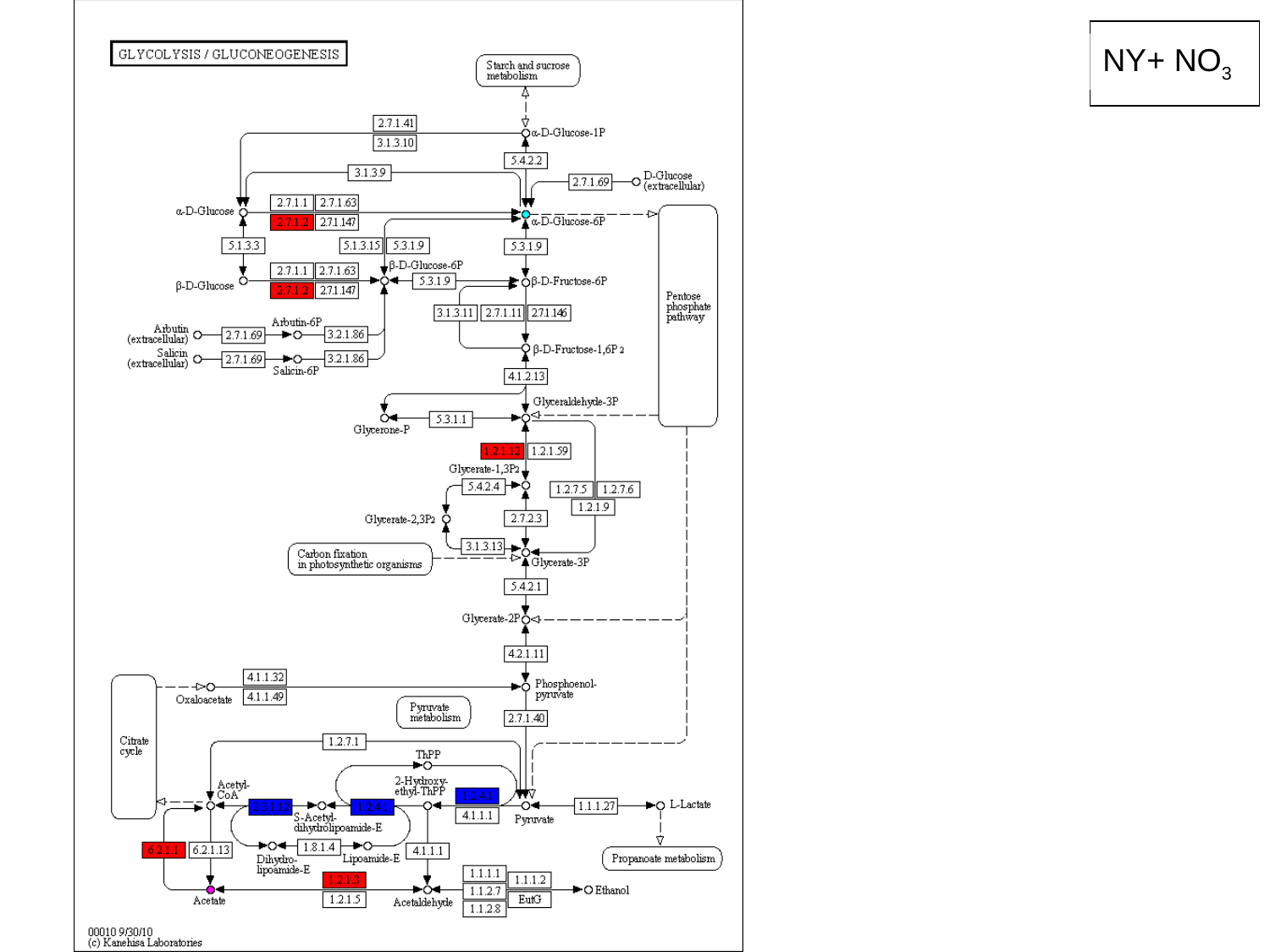

NY+ NO3

## Slide 16
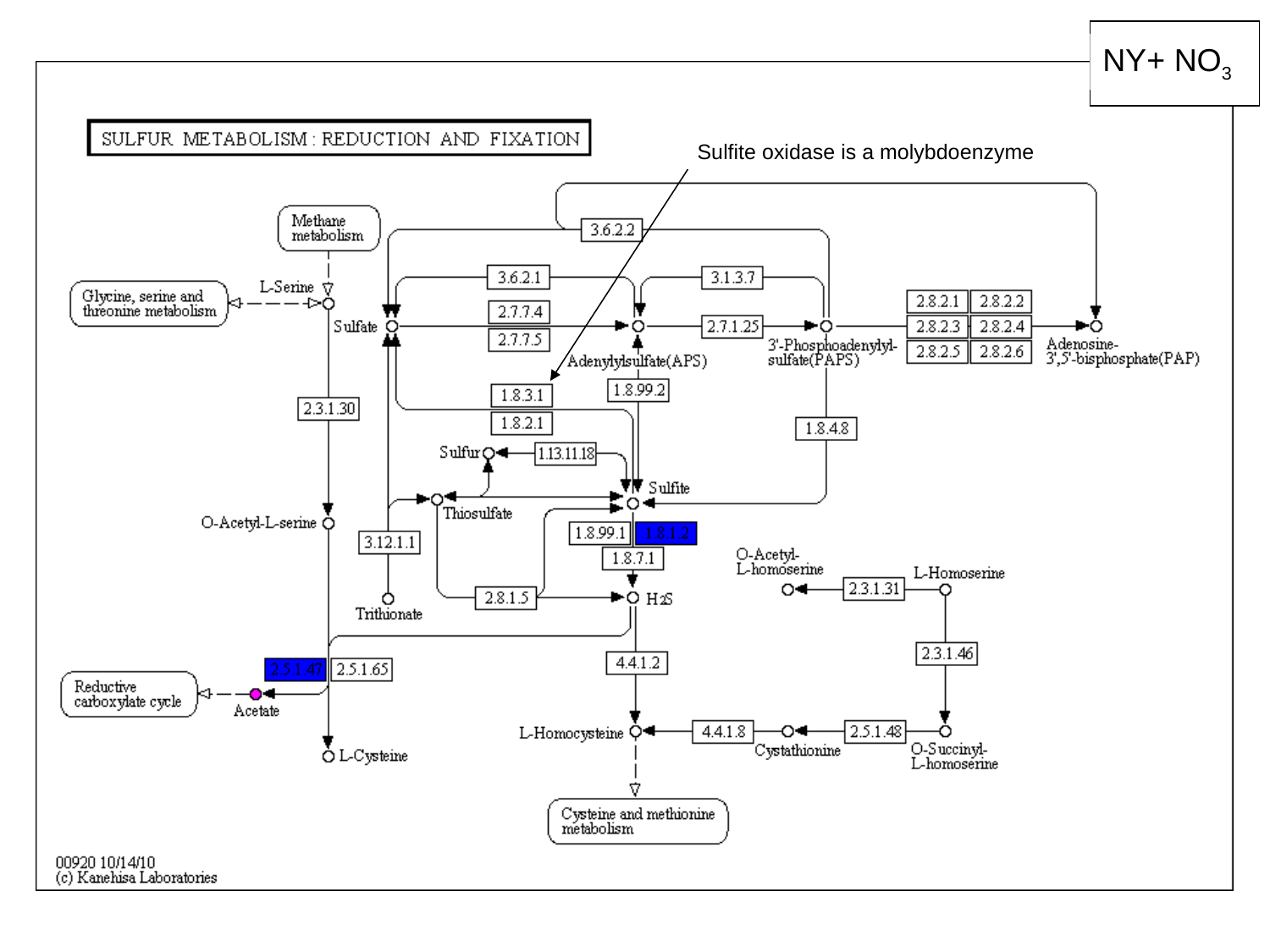

NY+ NO3
Sulfite oxidase is a molybdoenzyme

## Slide 17
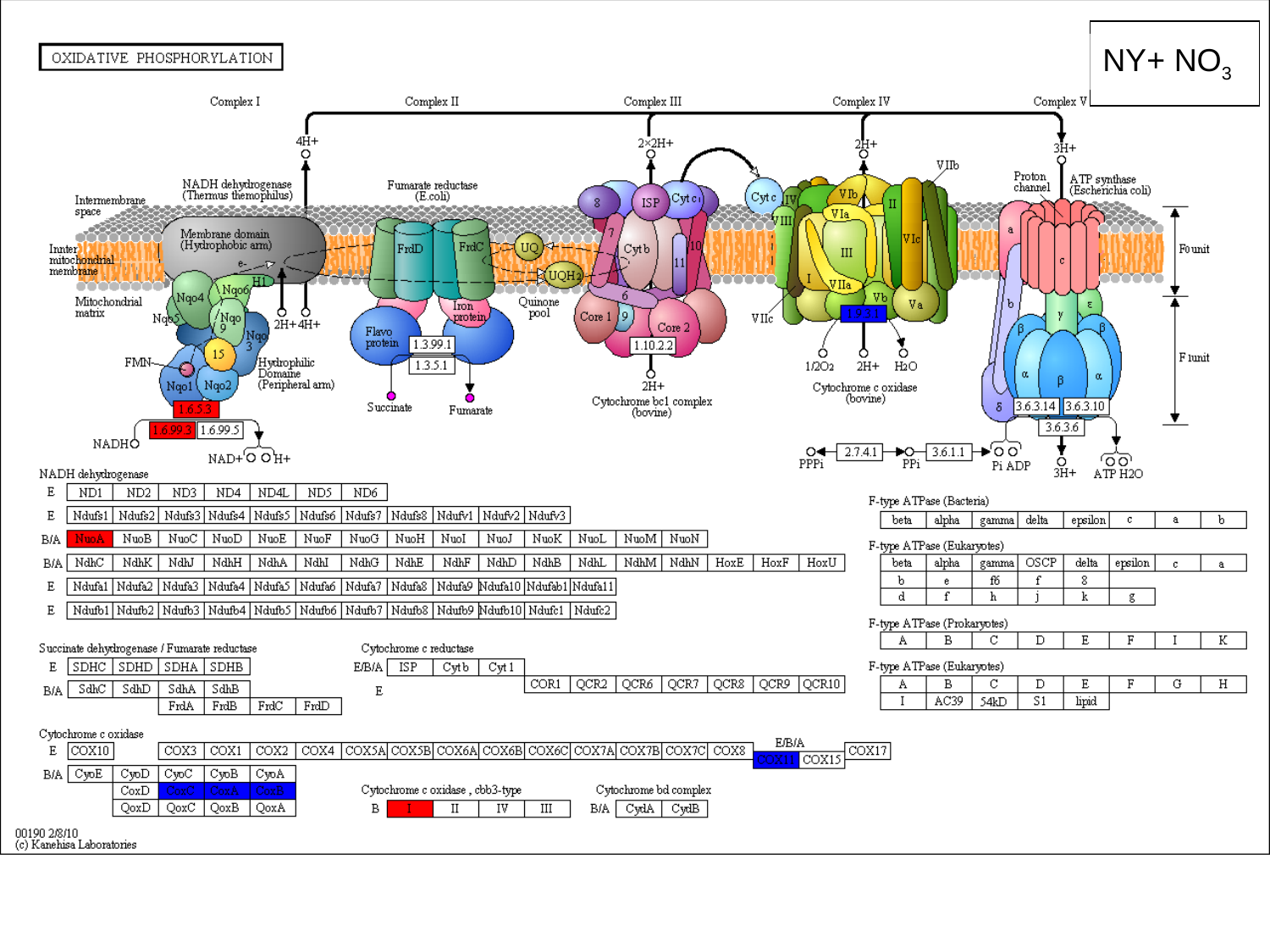

NY+ NO3

## Slide 18
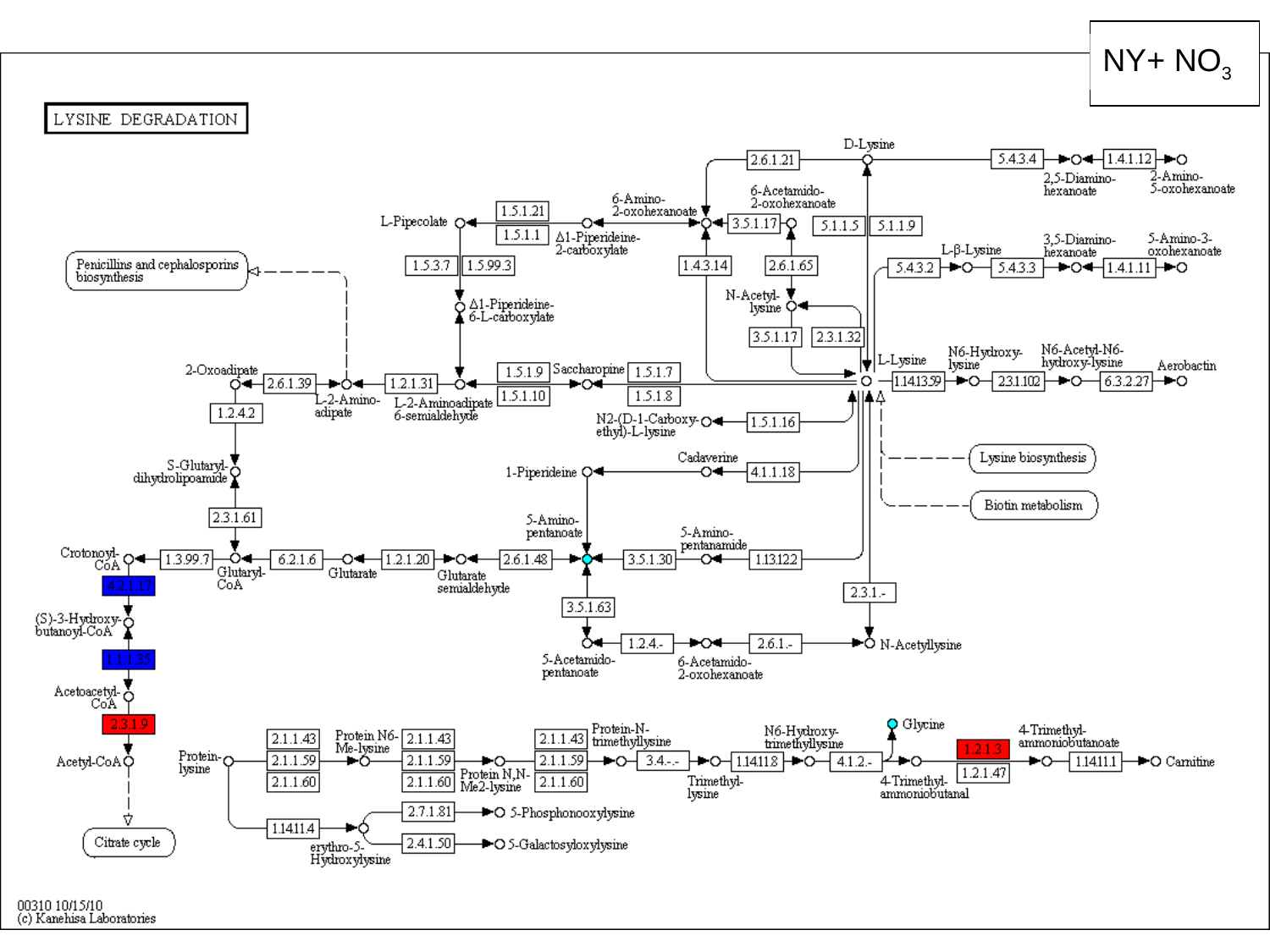

NY+ NO3

## Slide 19
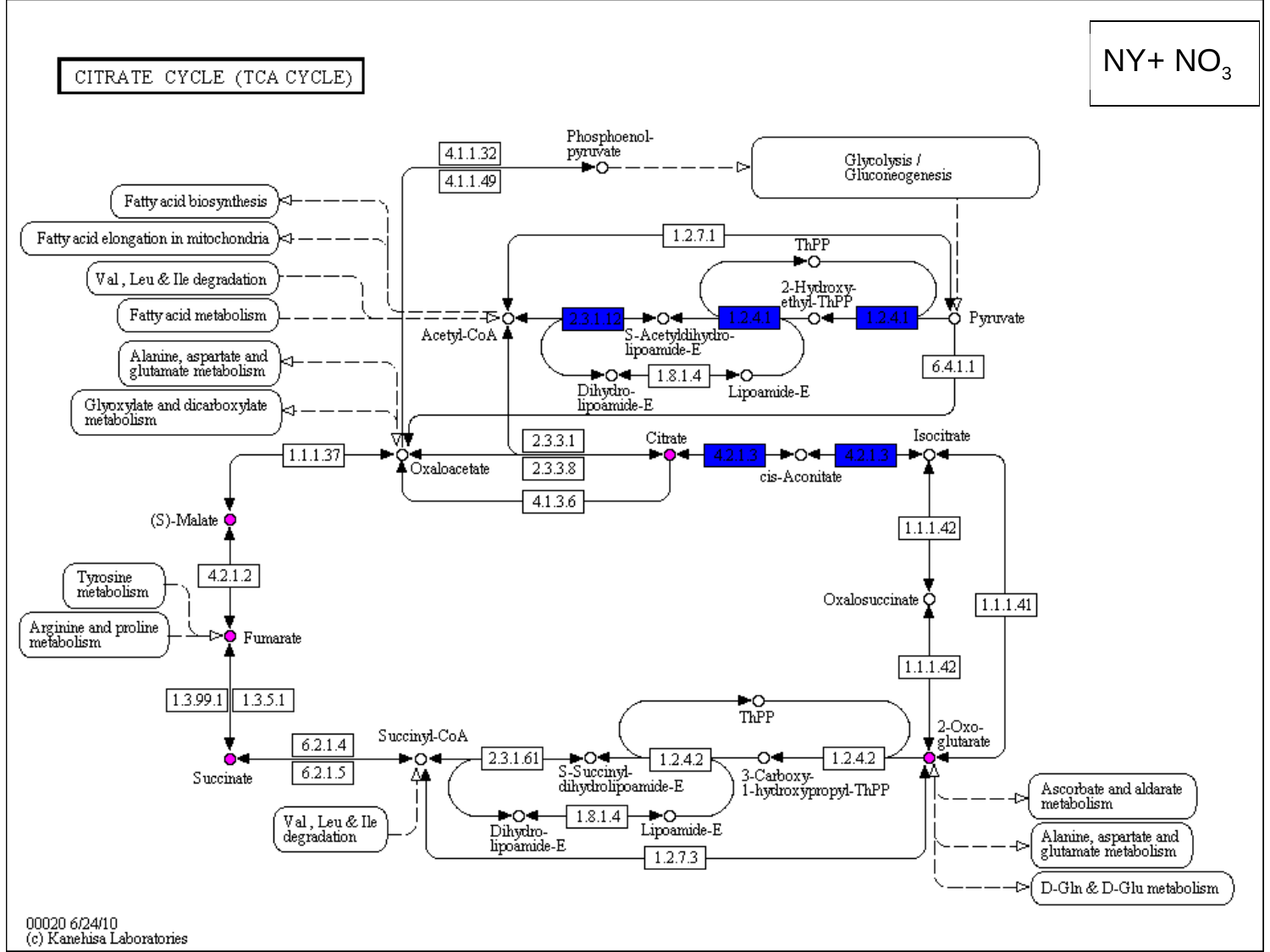

NY+ NO3

## Slide 20
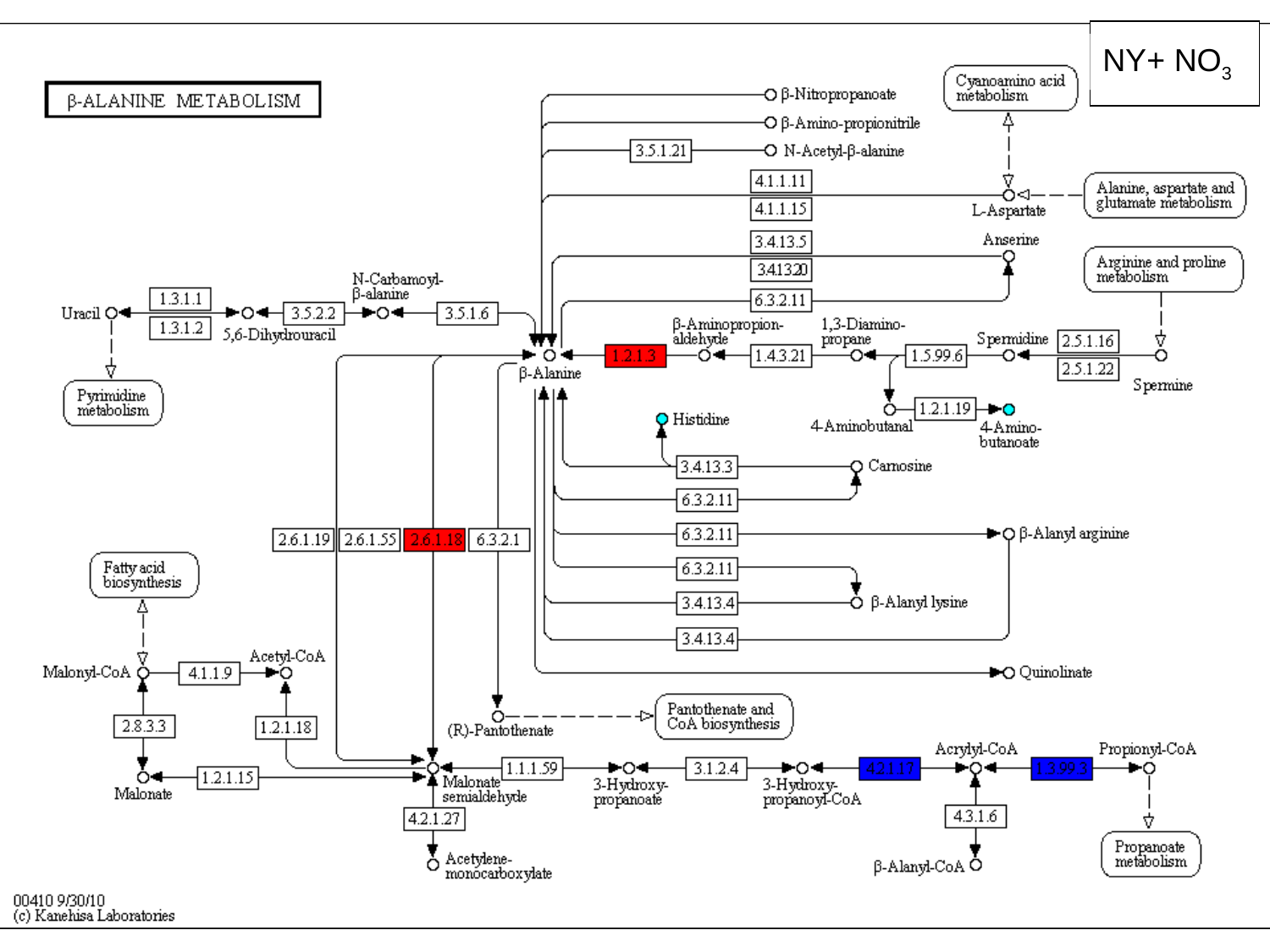

NY+ NO3

## Slide 21
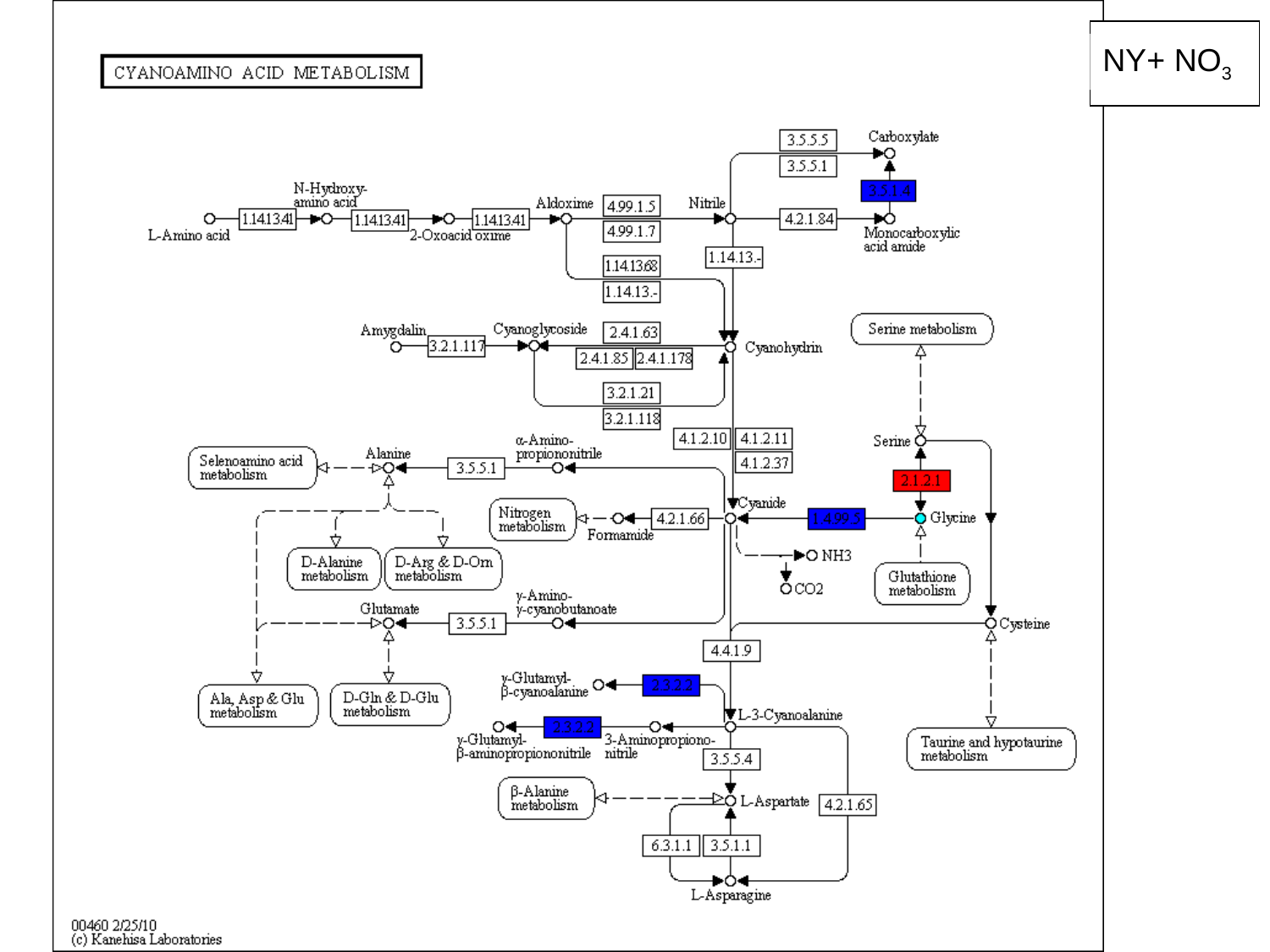

NY+ NO3

## Slide 22
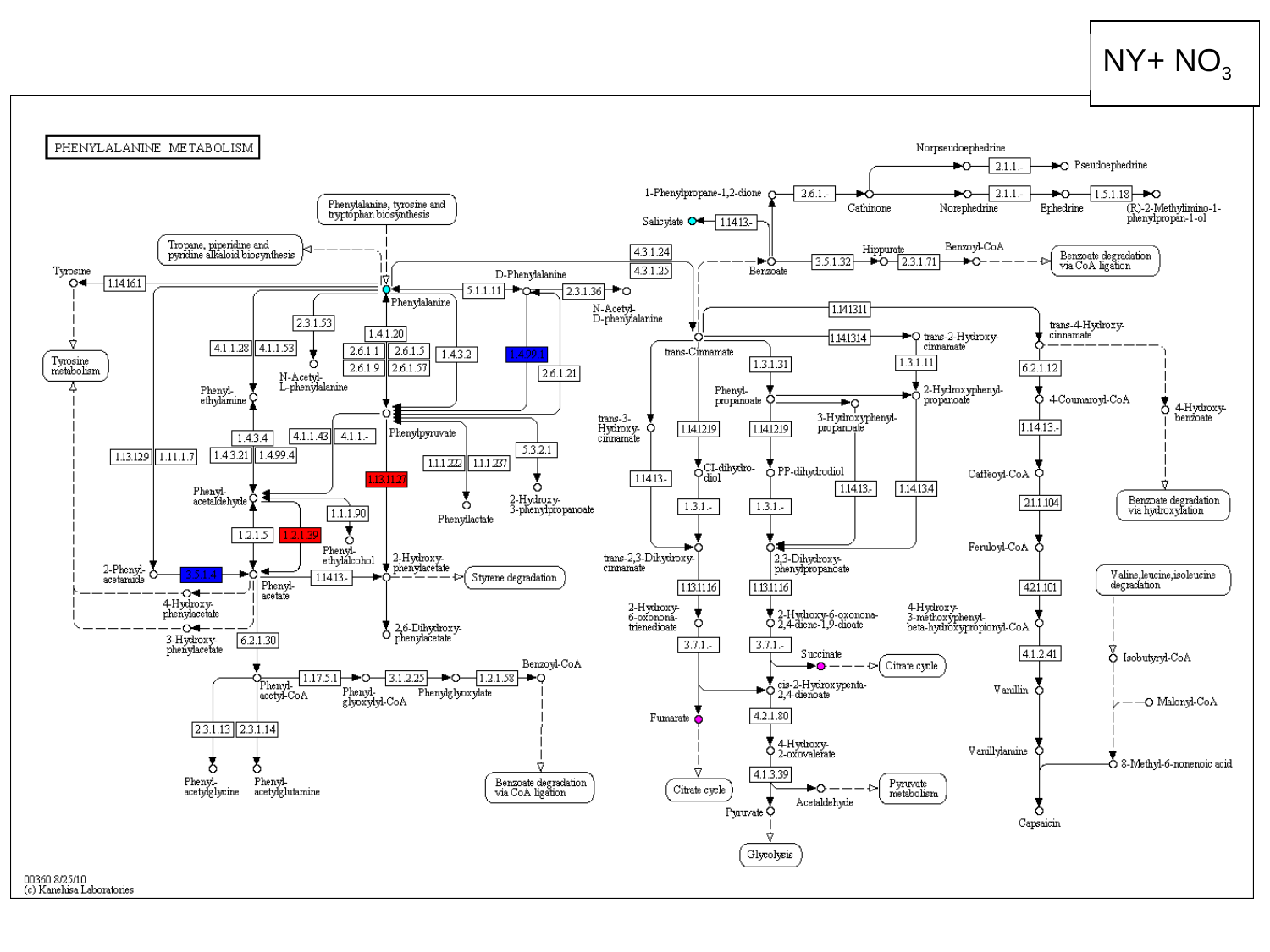

NY+ NO3

## Slide 23
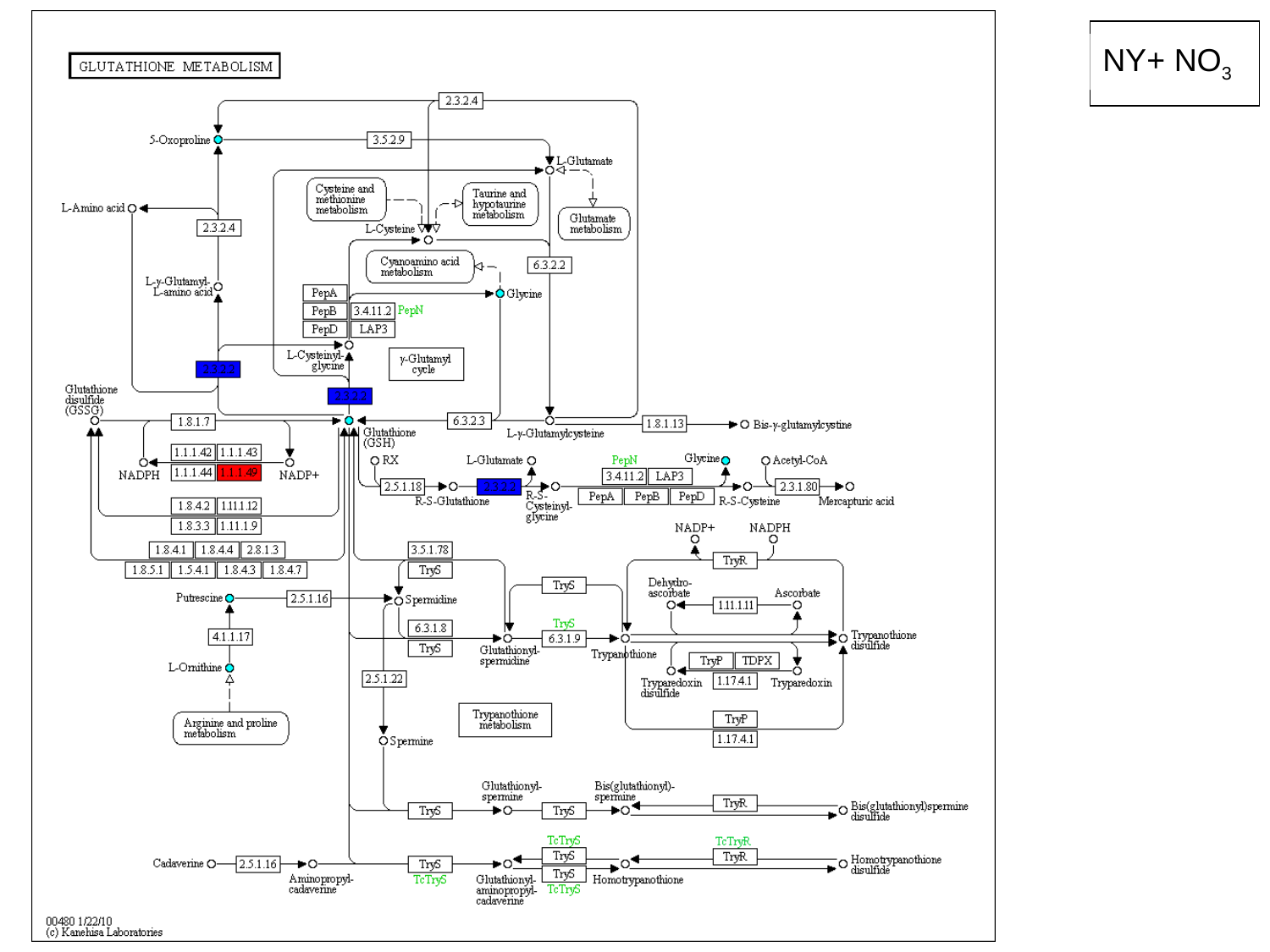

NY+ NO3

## Slide 24
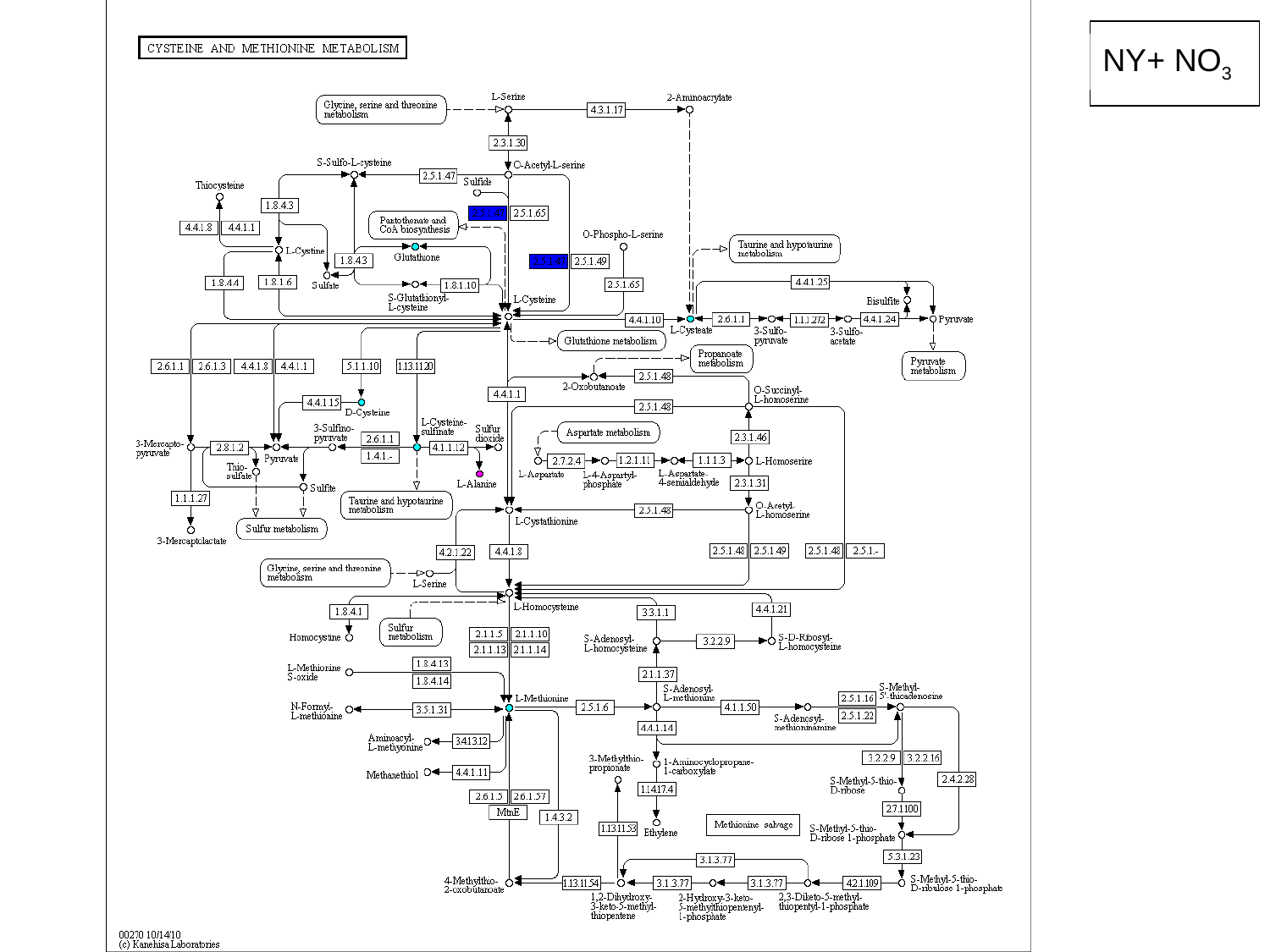

NY+ NO3

## Slide 25
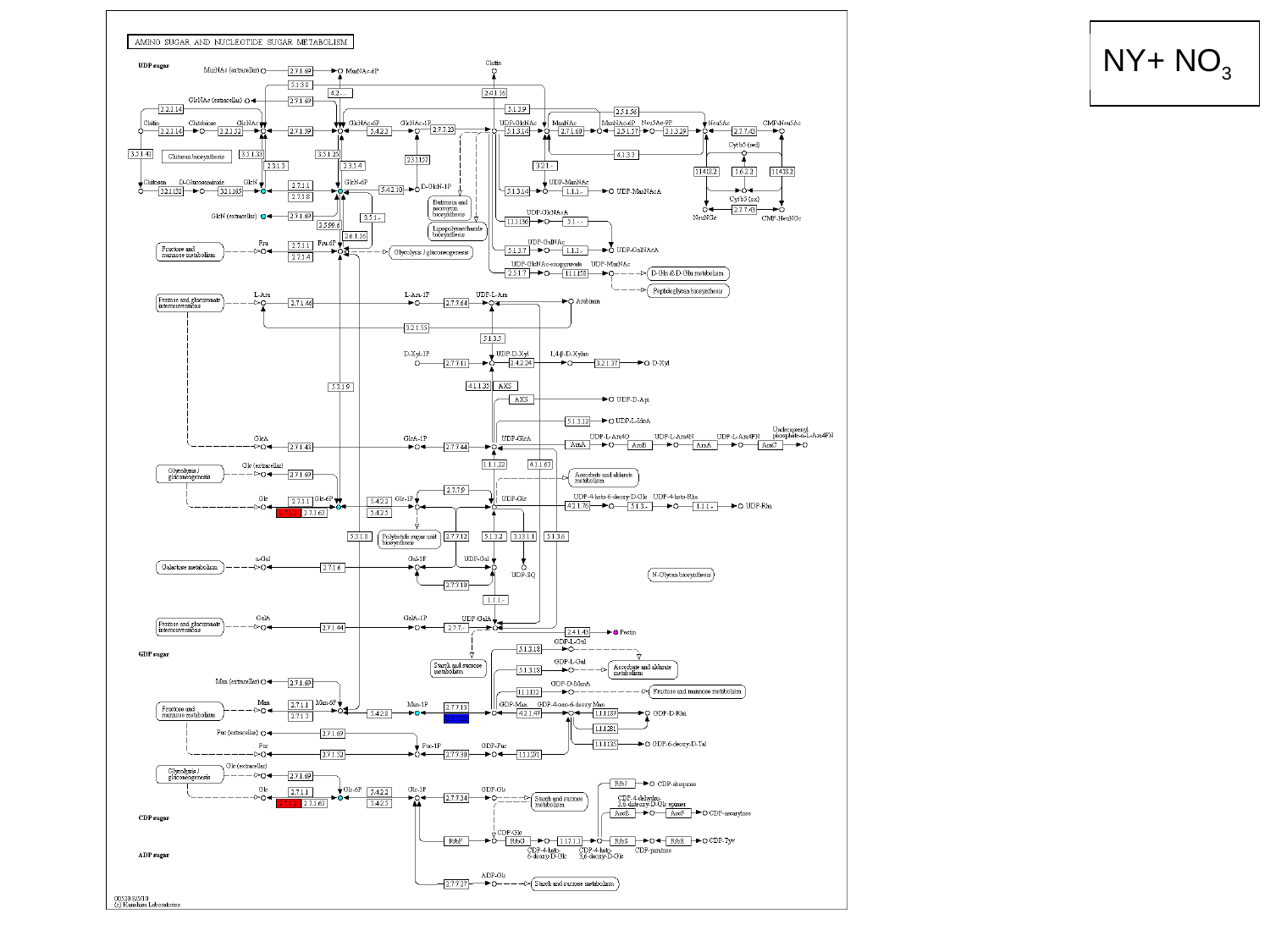

NY+ NO3

## Slide 26
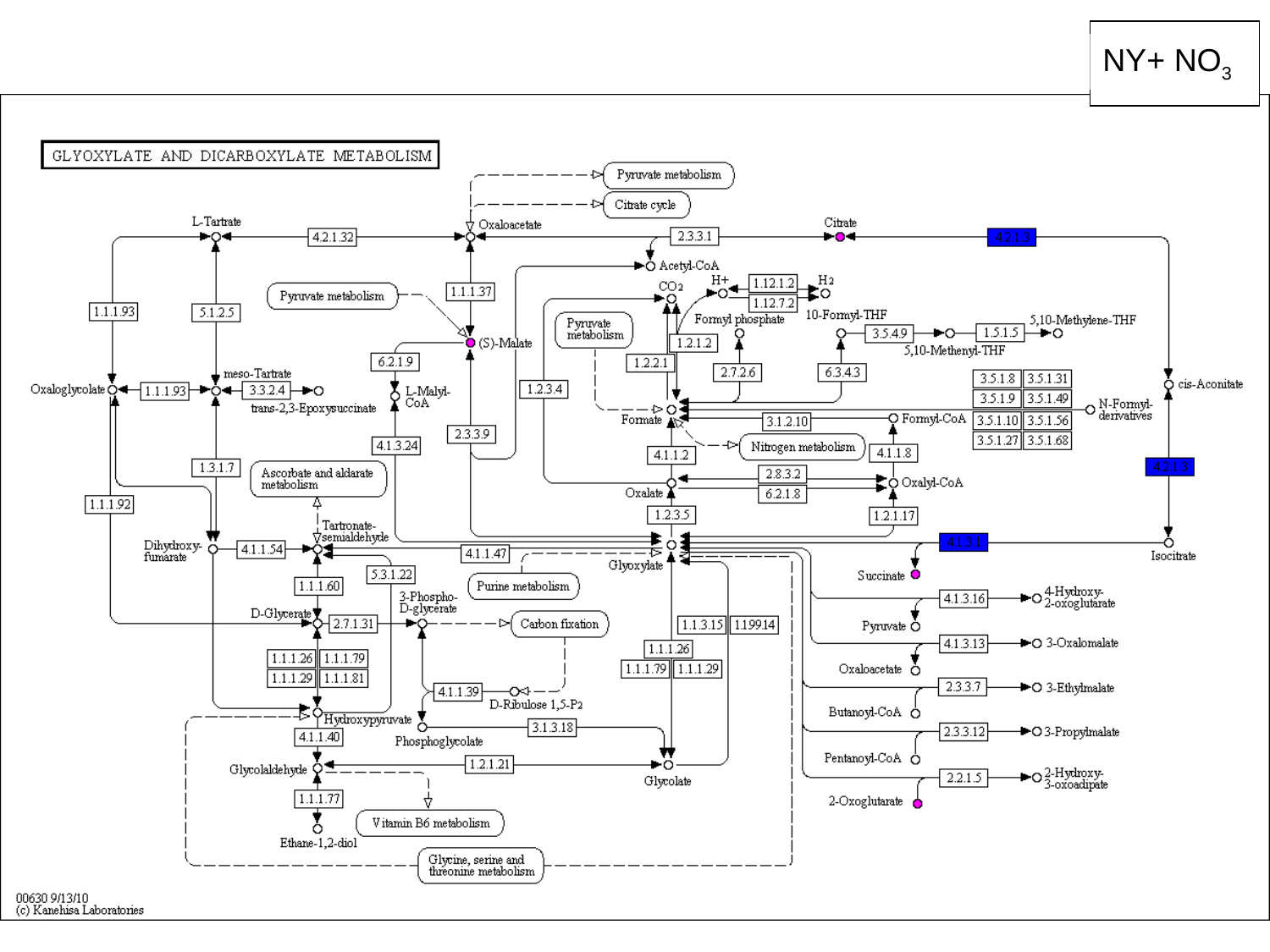

NY+ NO3

## Slide 27
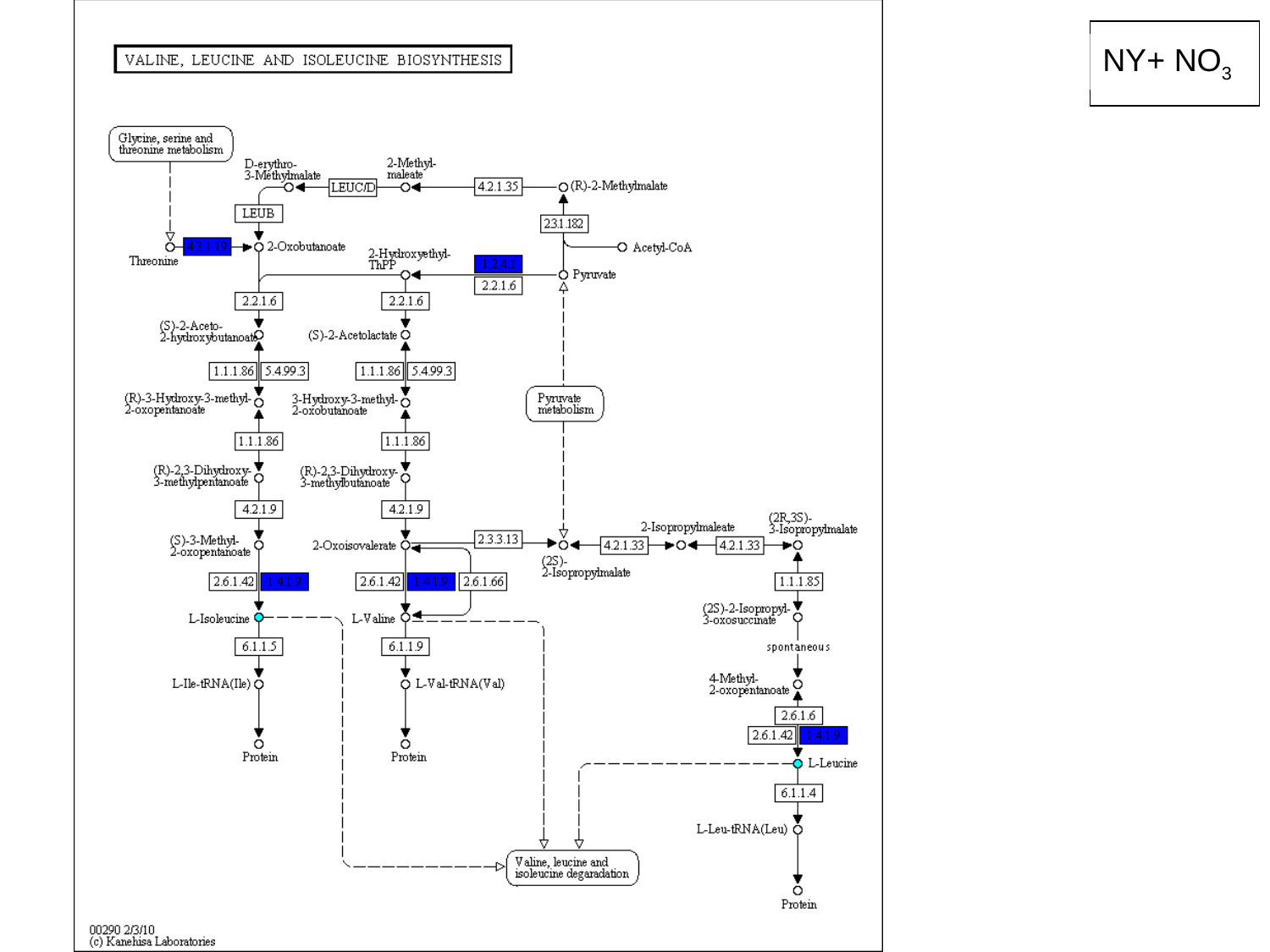

NY+ NO3

## Slide 28
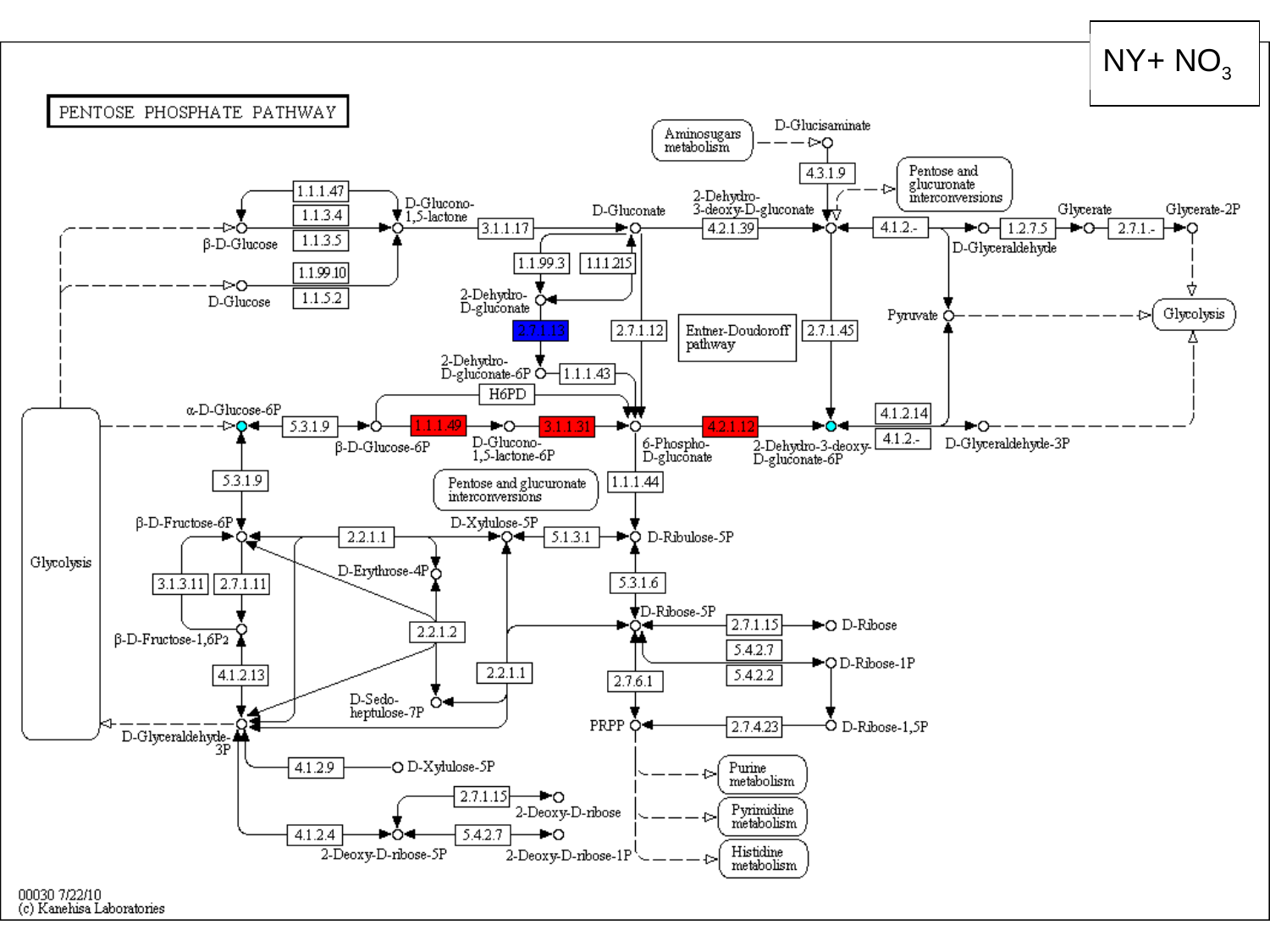

NY+ NO3

## Slide 29
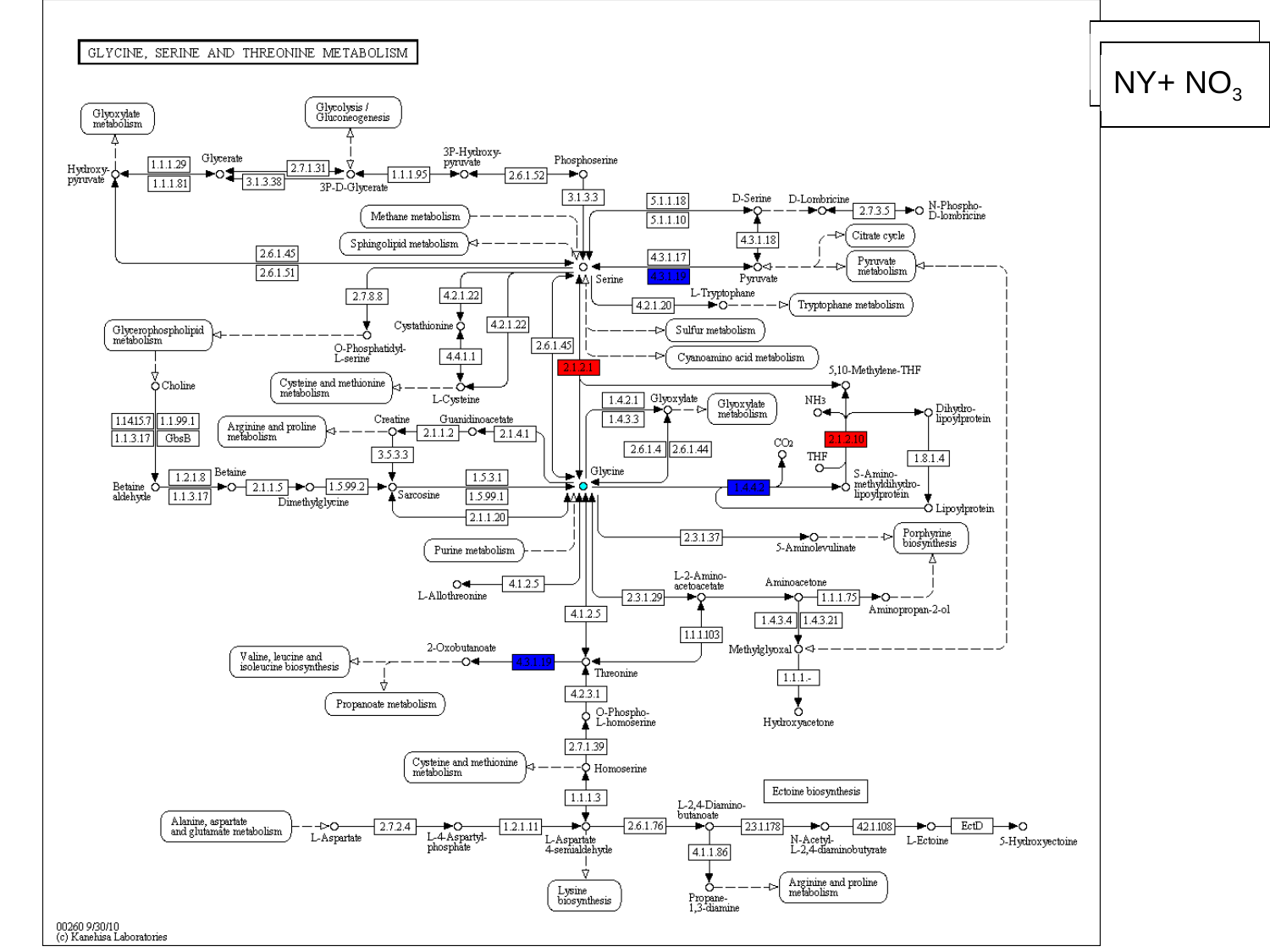

NY+ NO3
NY+ NO3

## Slide 30
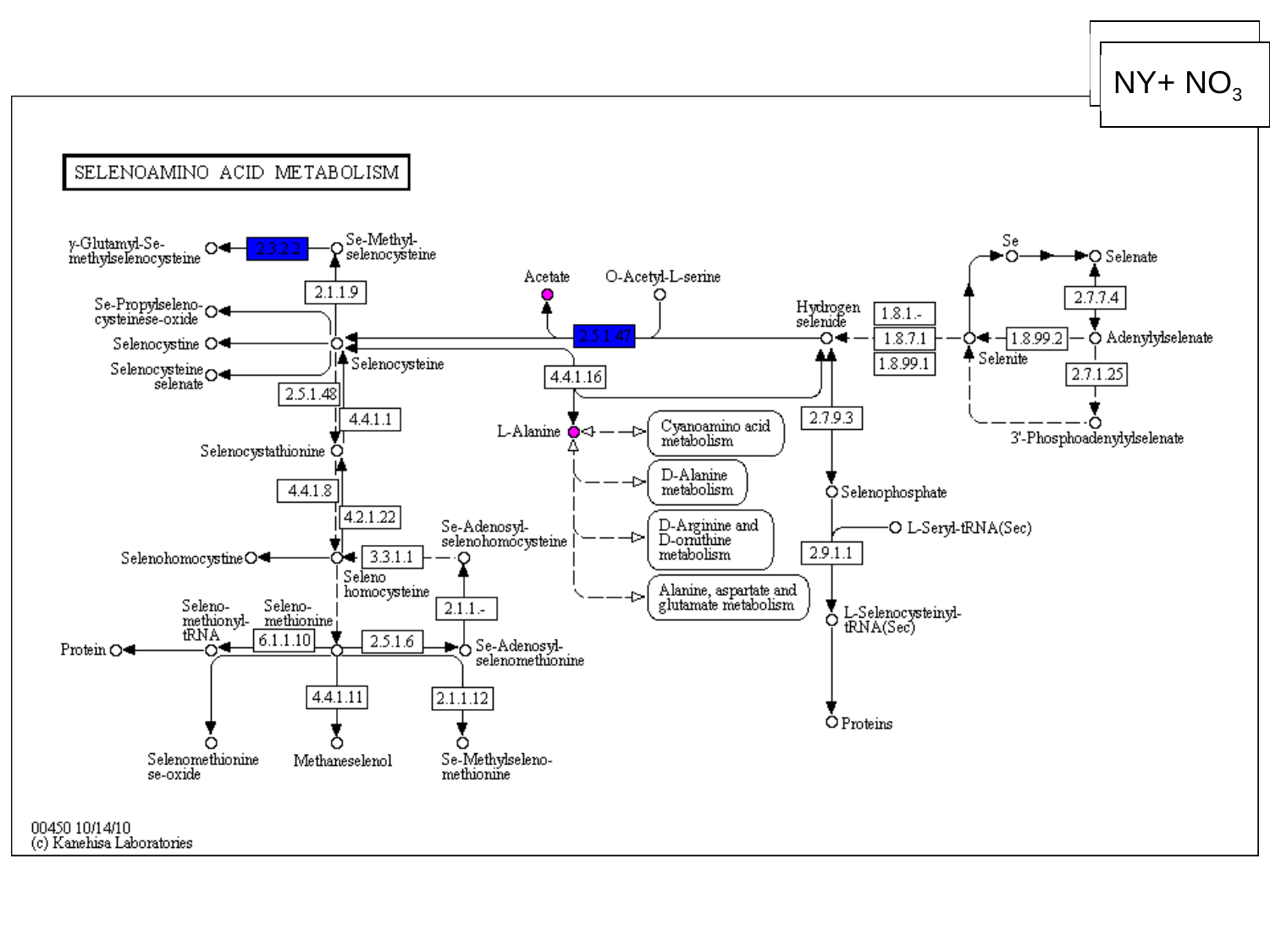

NY+ NO3
NY+ NO3

## Slide 31
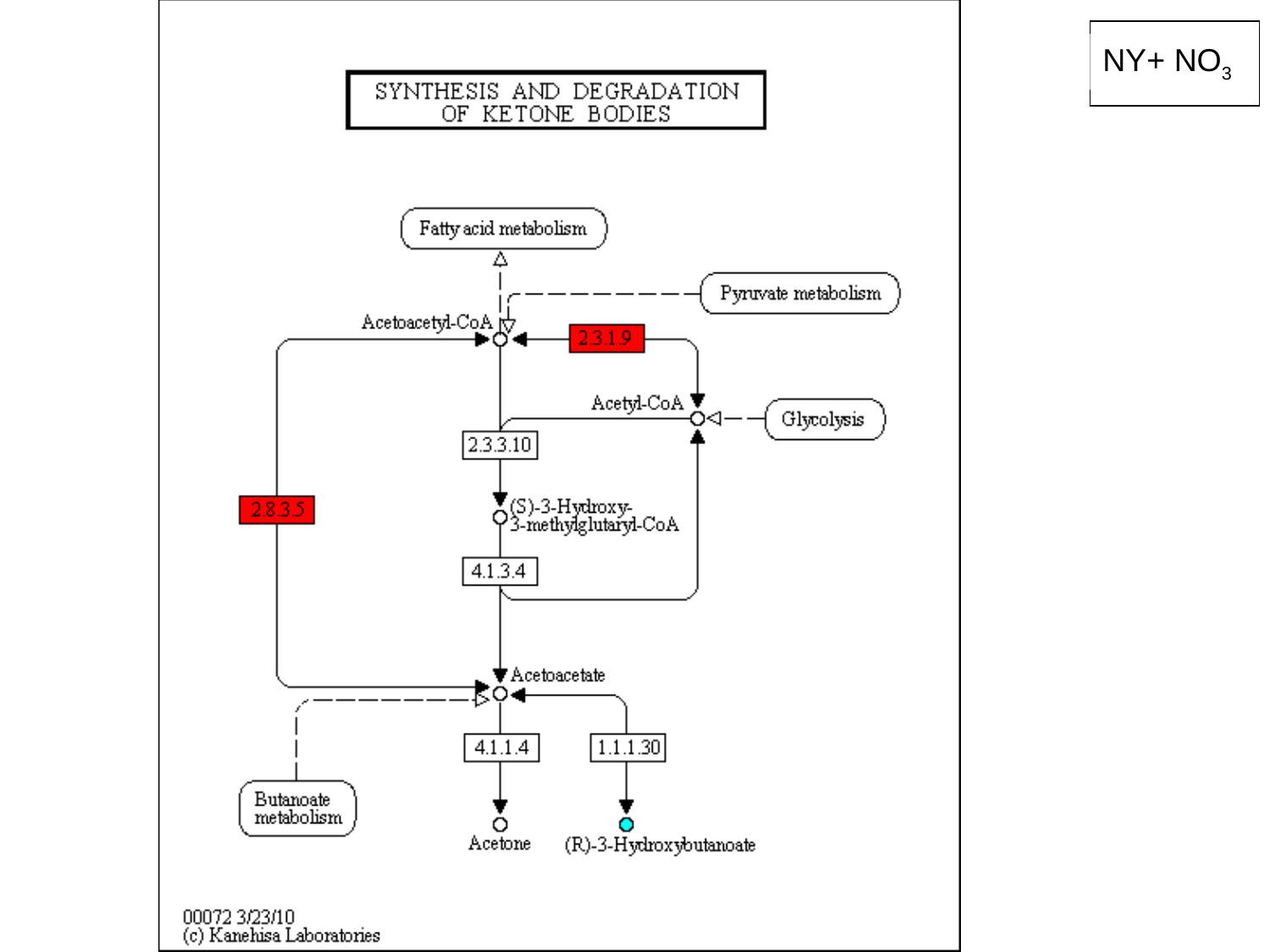

NY+ NO3

## Slide 32
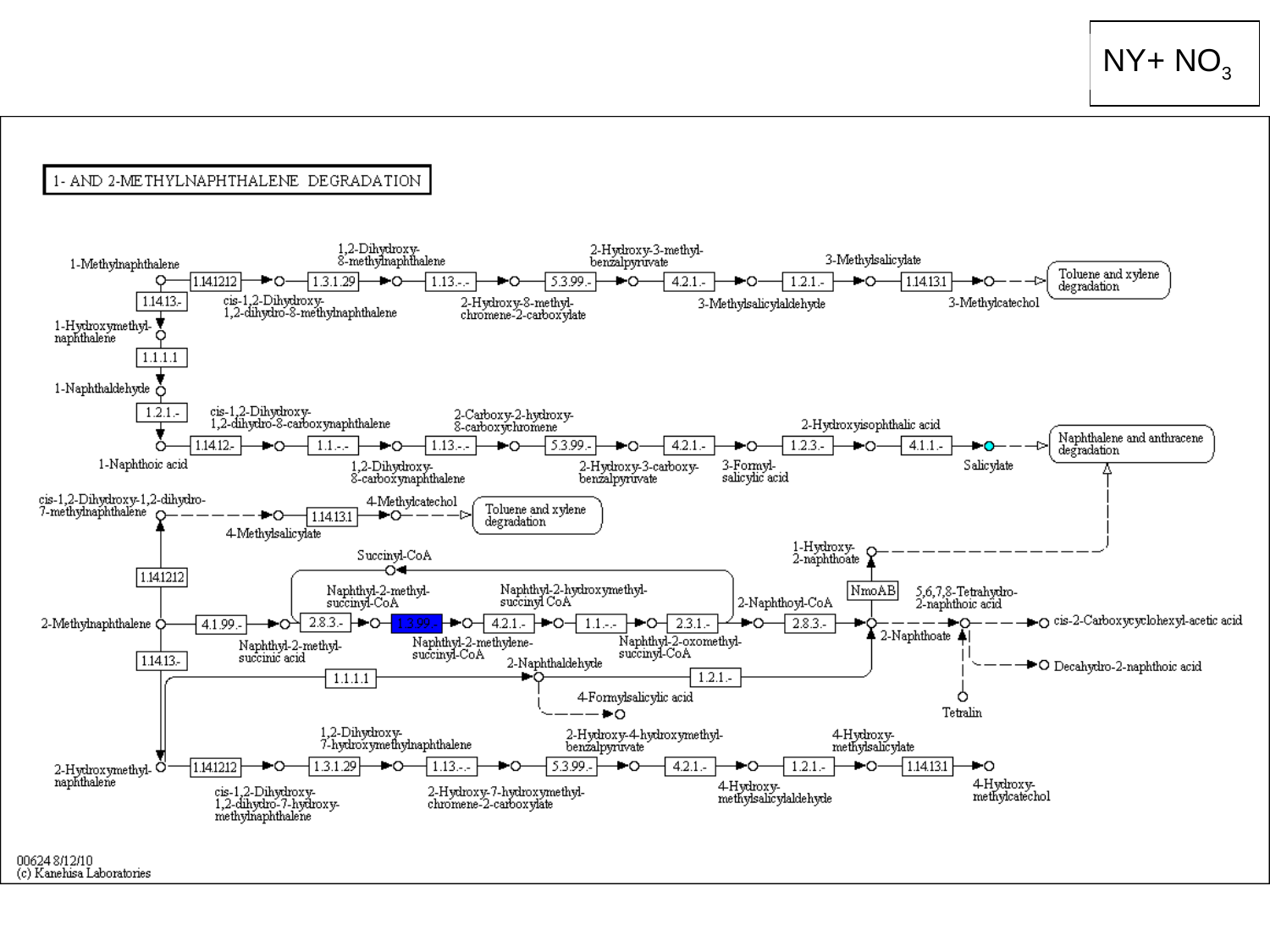

NY+ NO3

## Slide 33
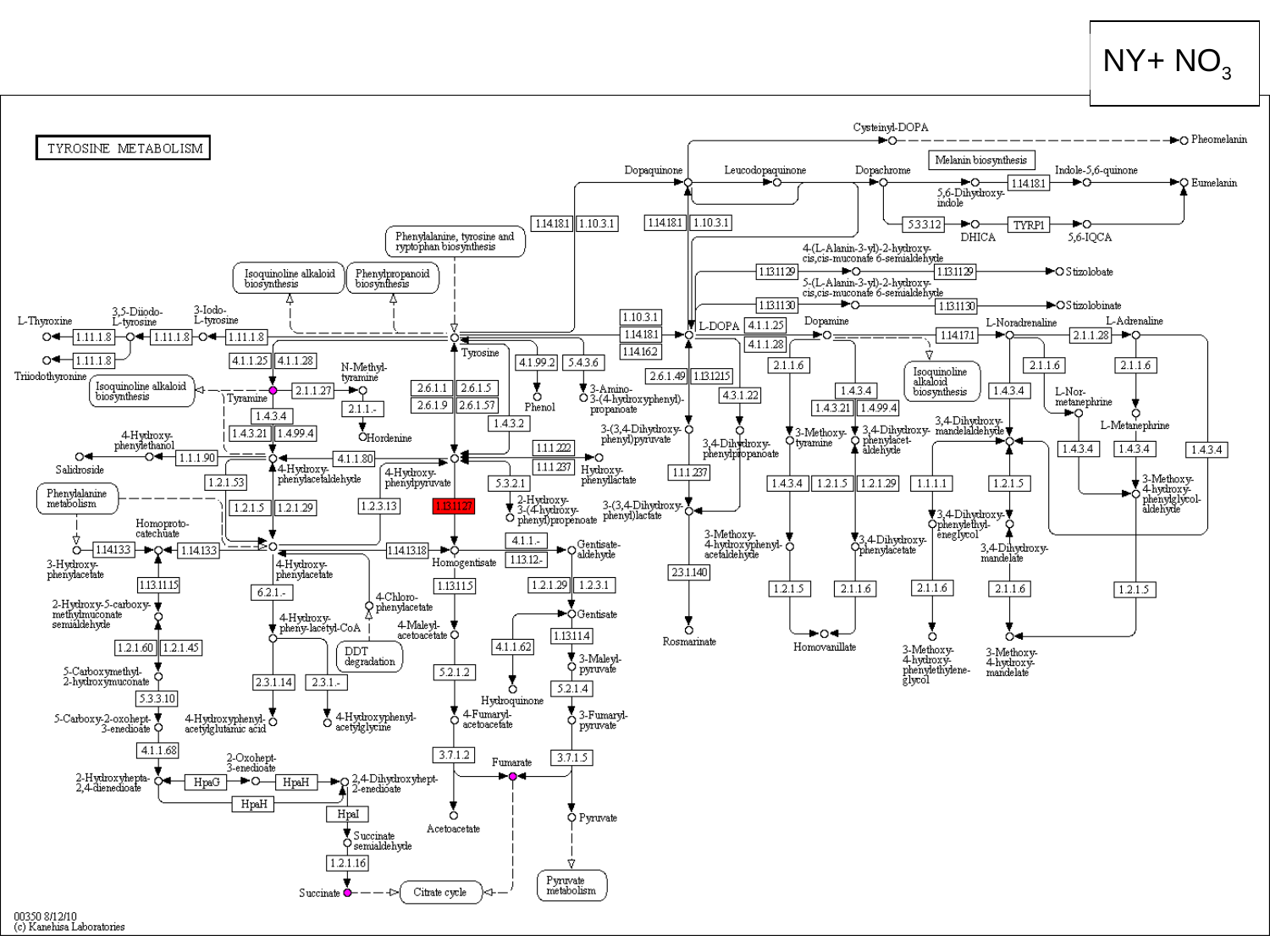

NY+ NO3

## Slide 34
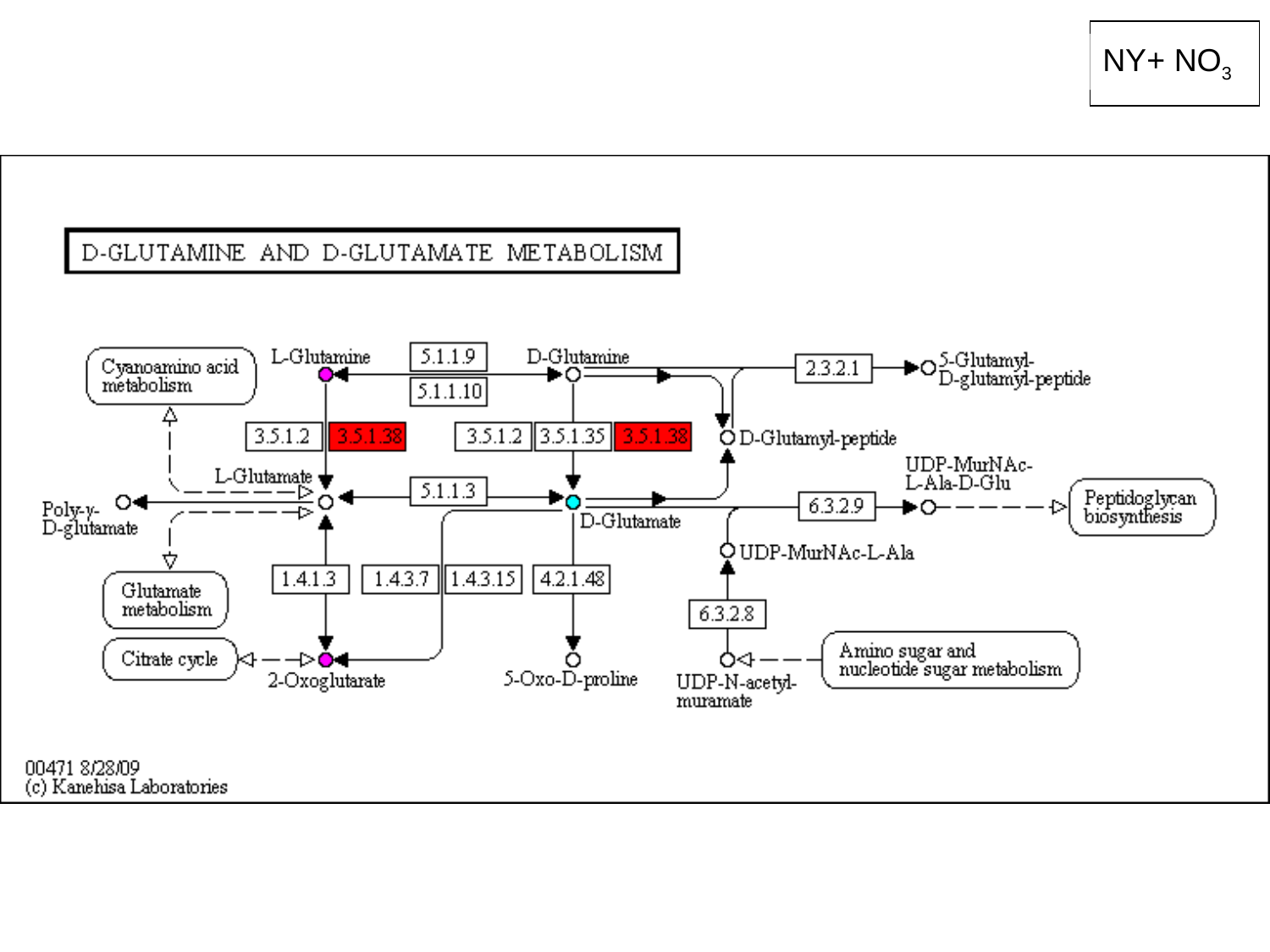

NY+ NO3

## Slide 35
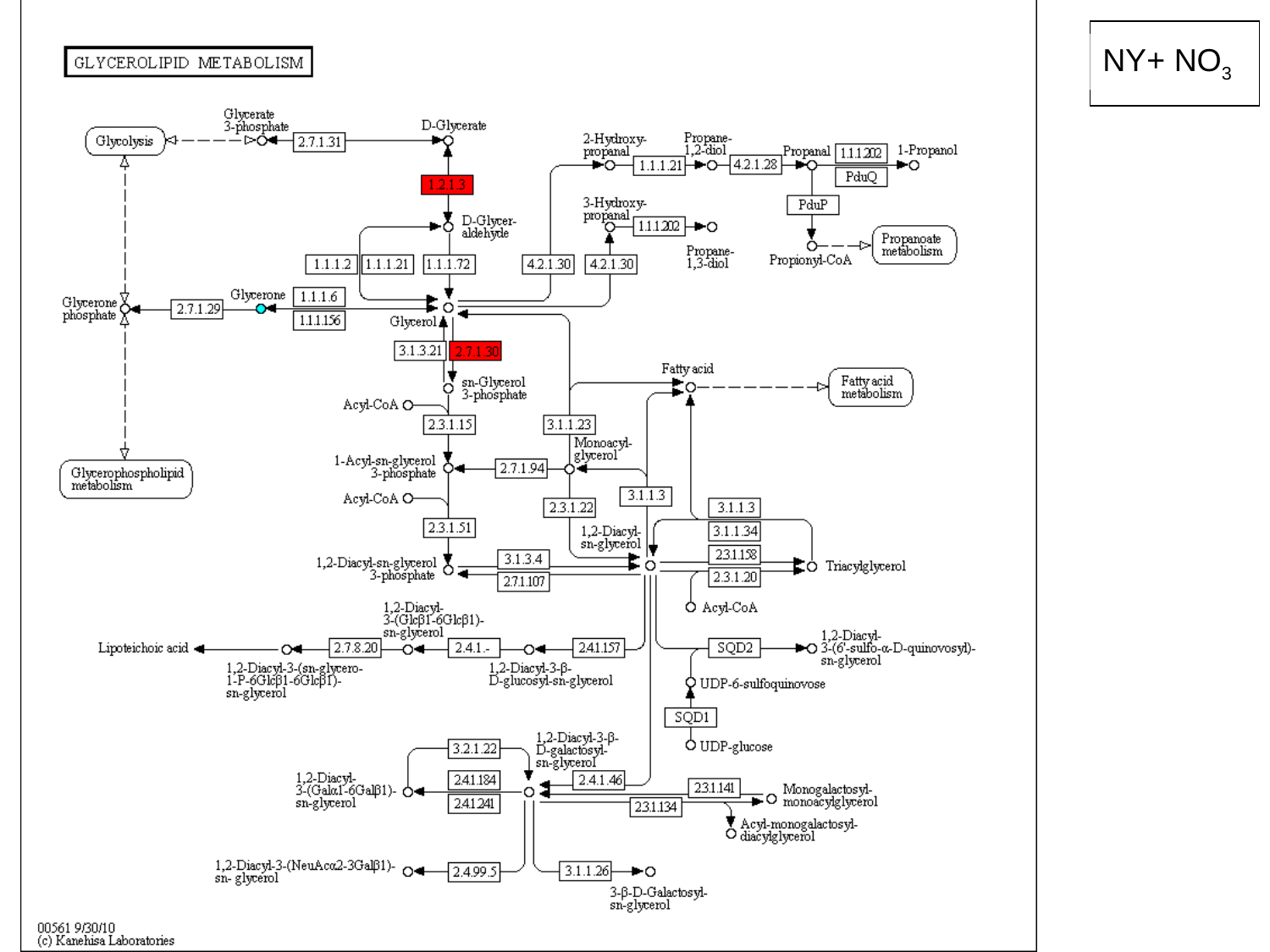

NY+ NO3

## Slide 36
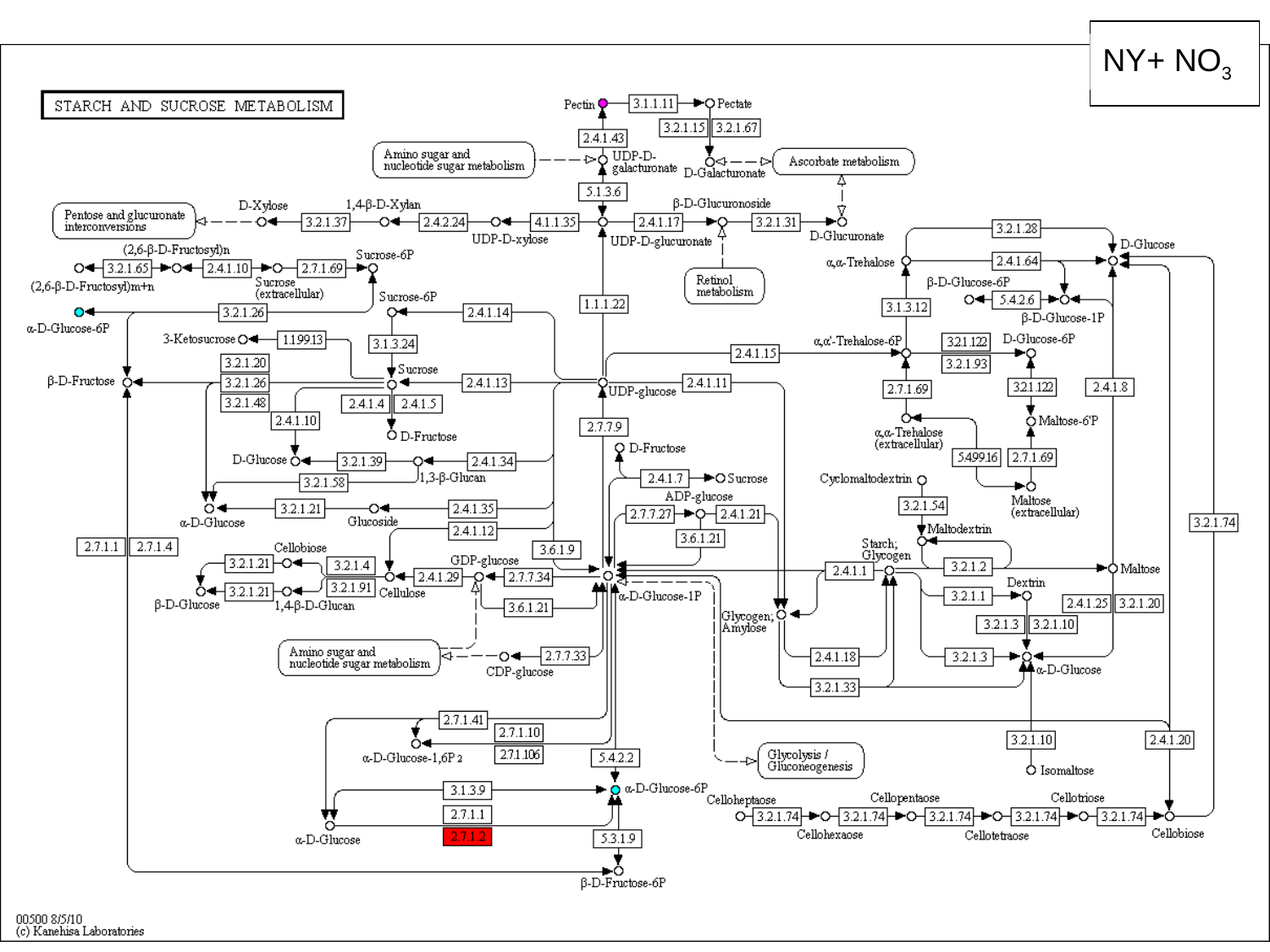

NY+ NO3

## Slide 37
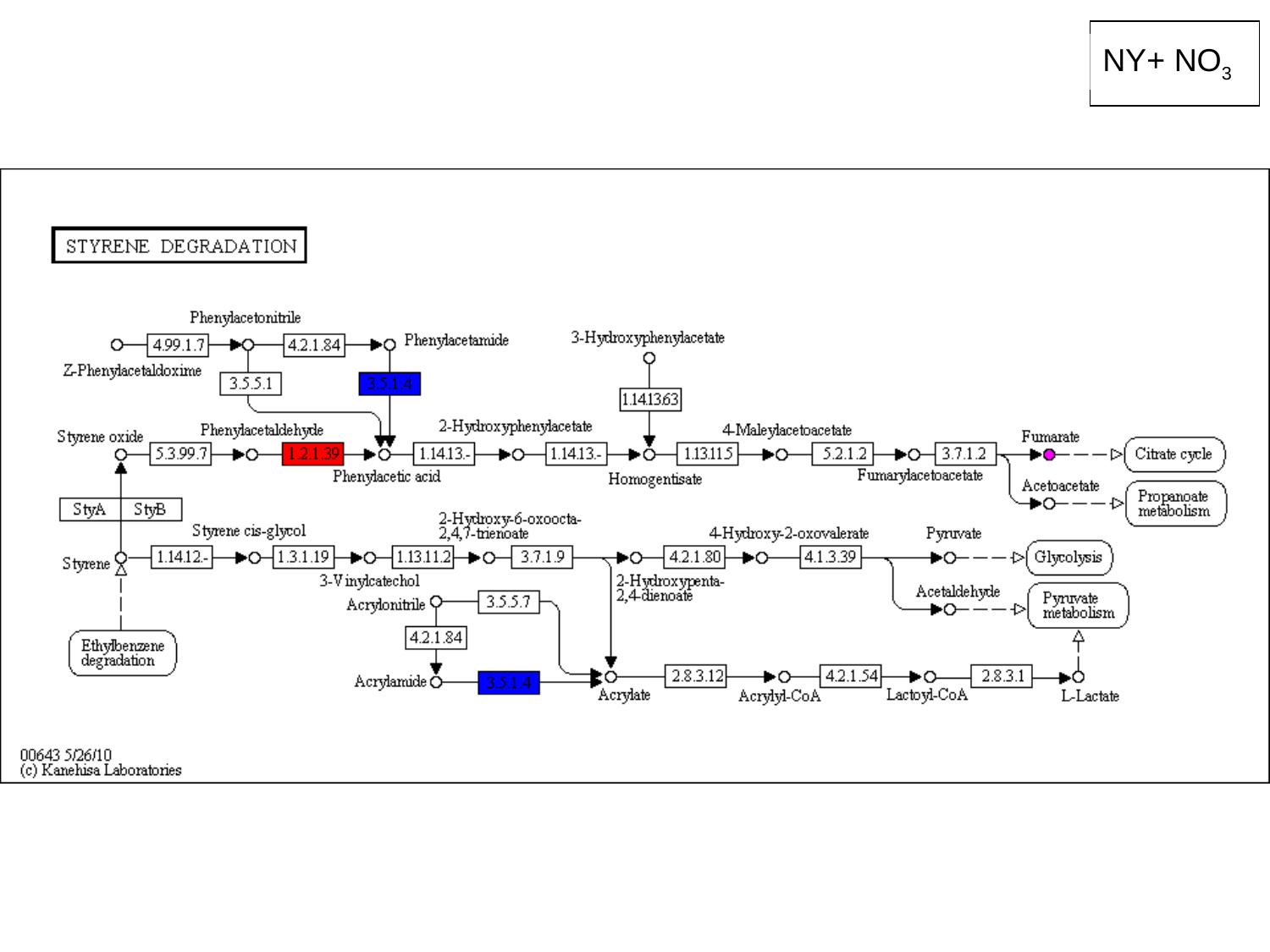

NY+ NO3

## Slide 38
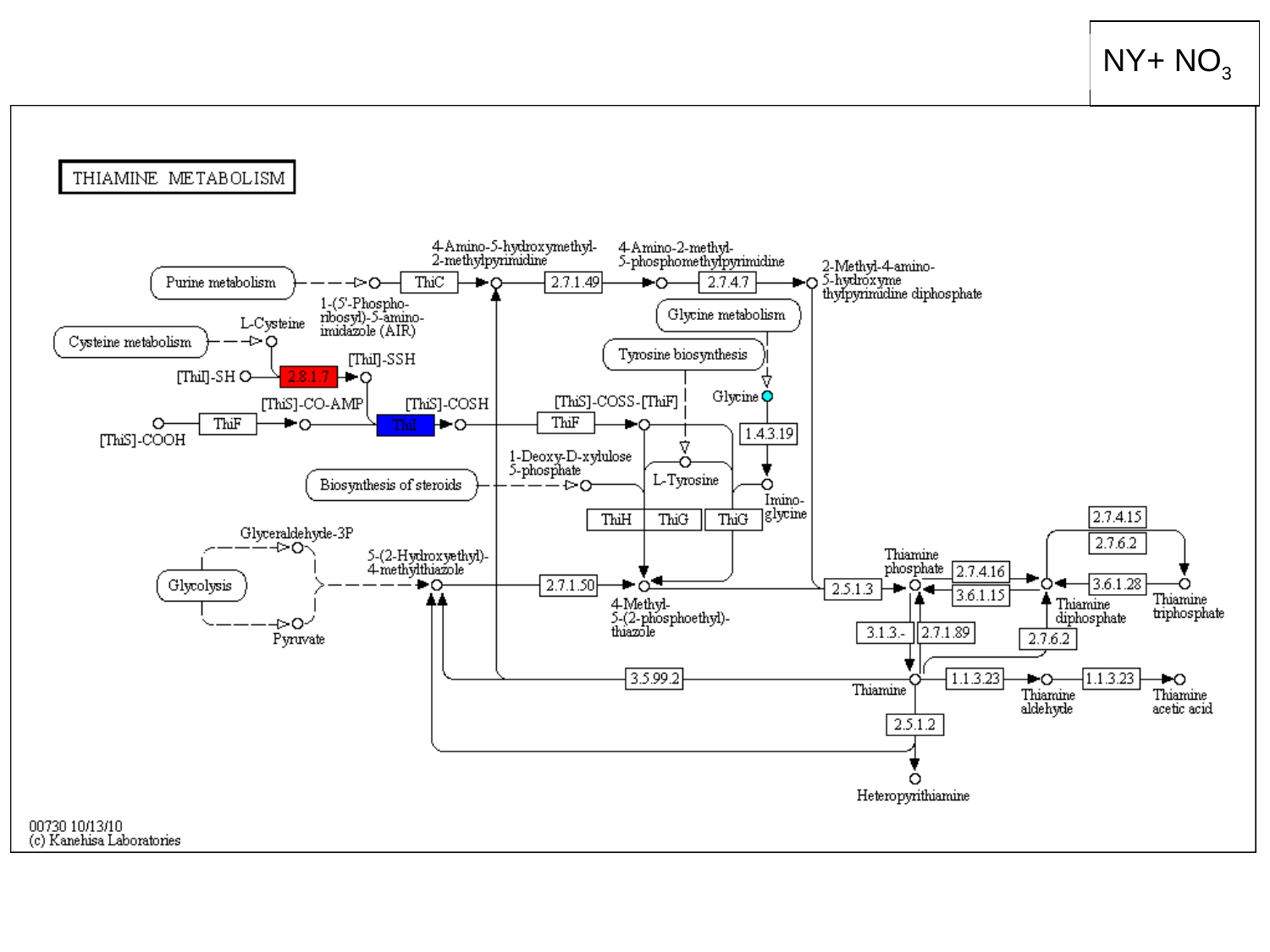

NY+ NO3

## Slide 39
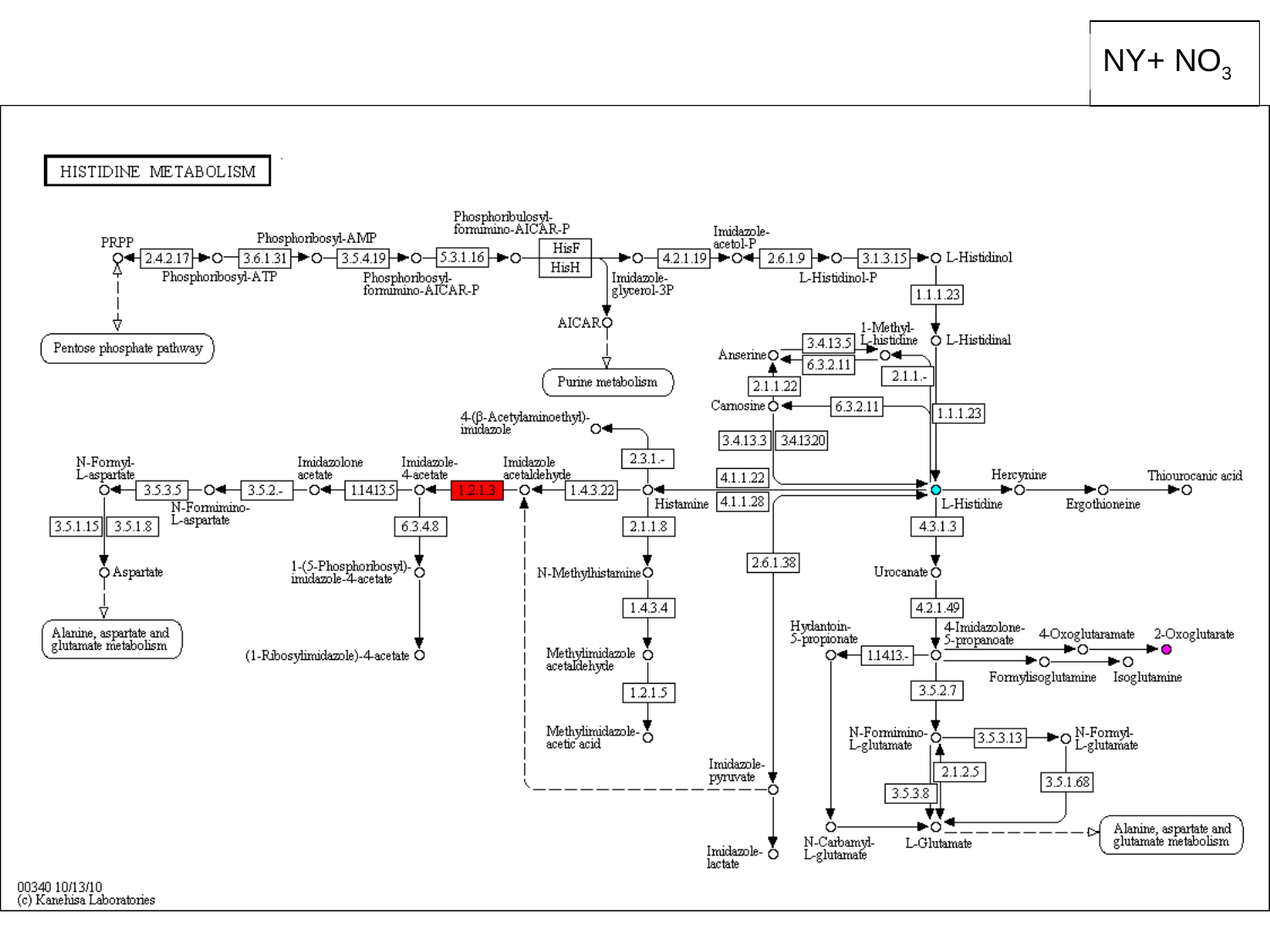

NY+ NO3

## Slide 40
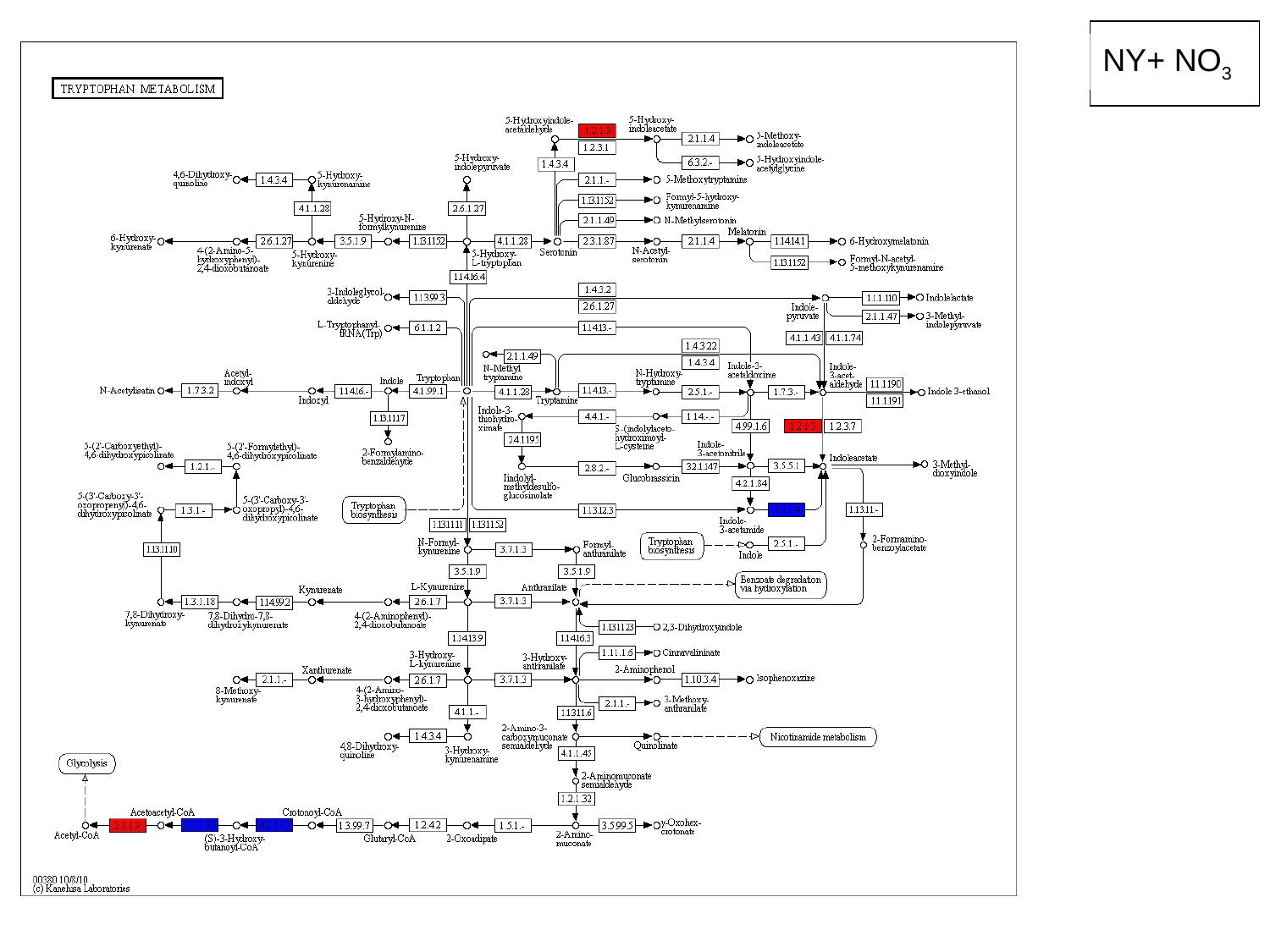

NY+ NO3

## Slide 41
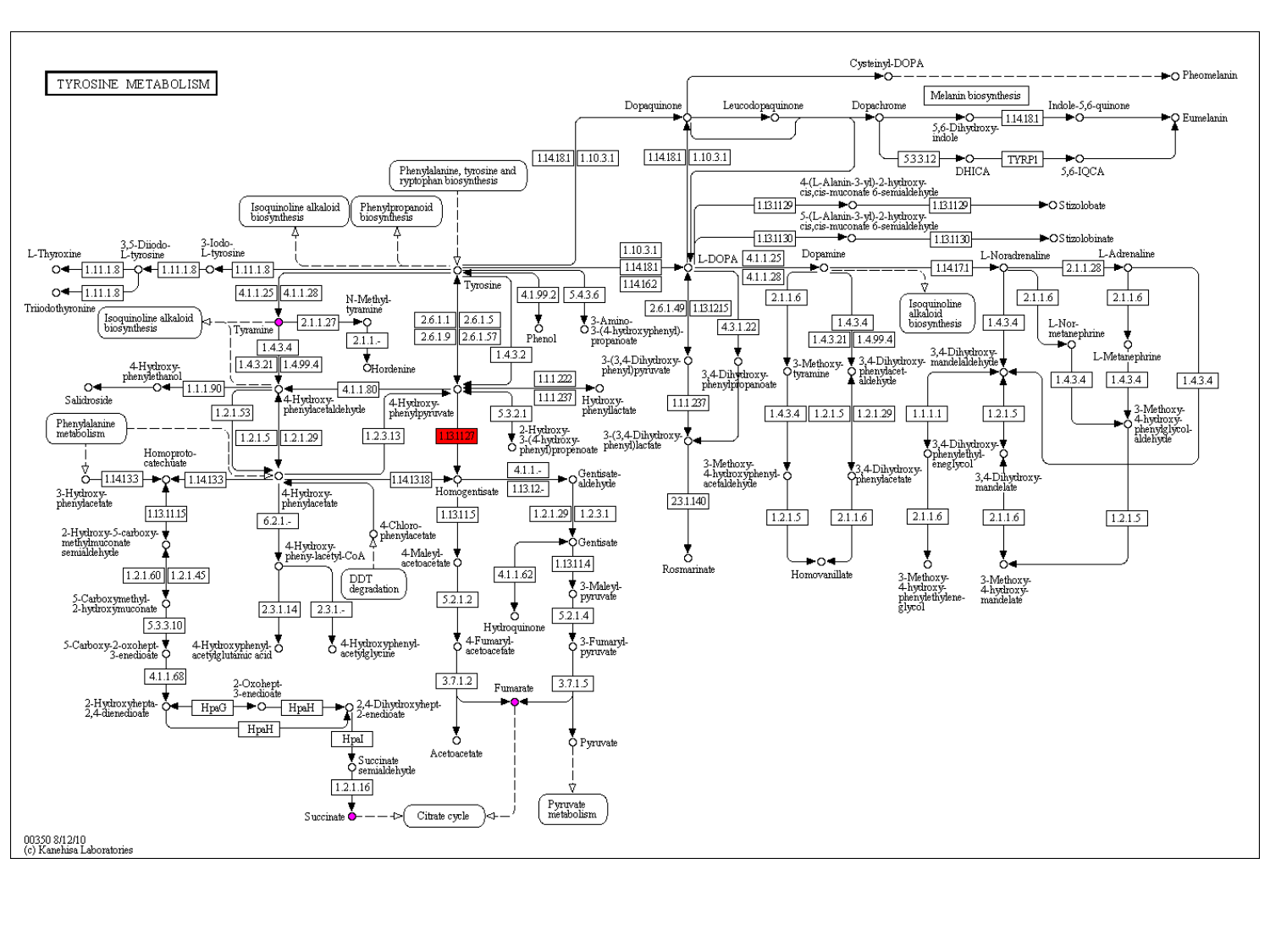

## Slide 42
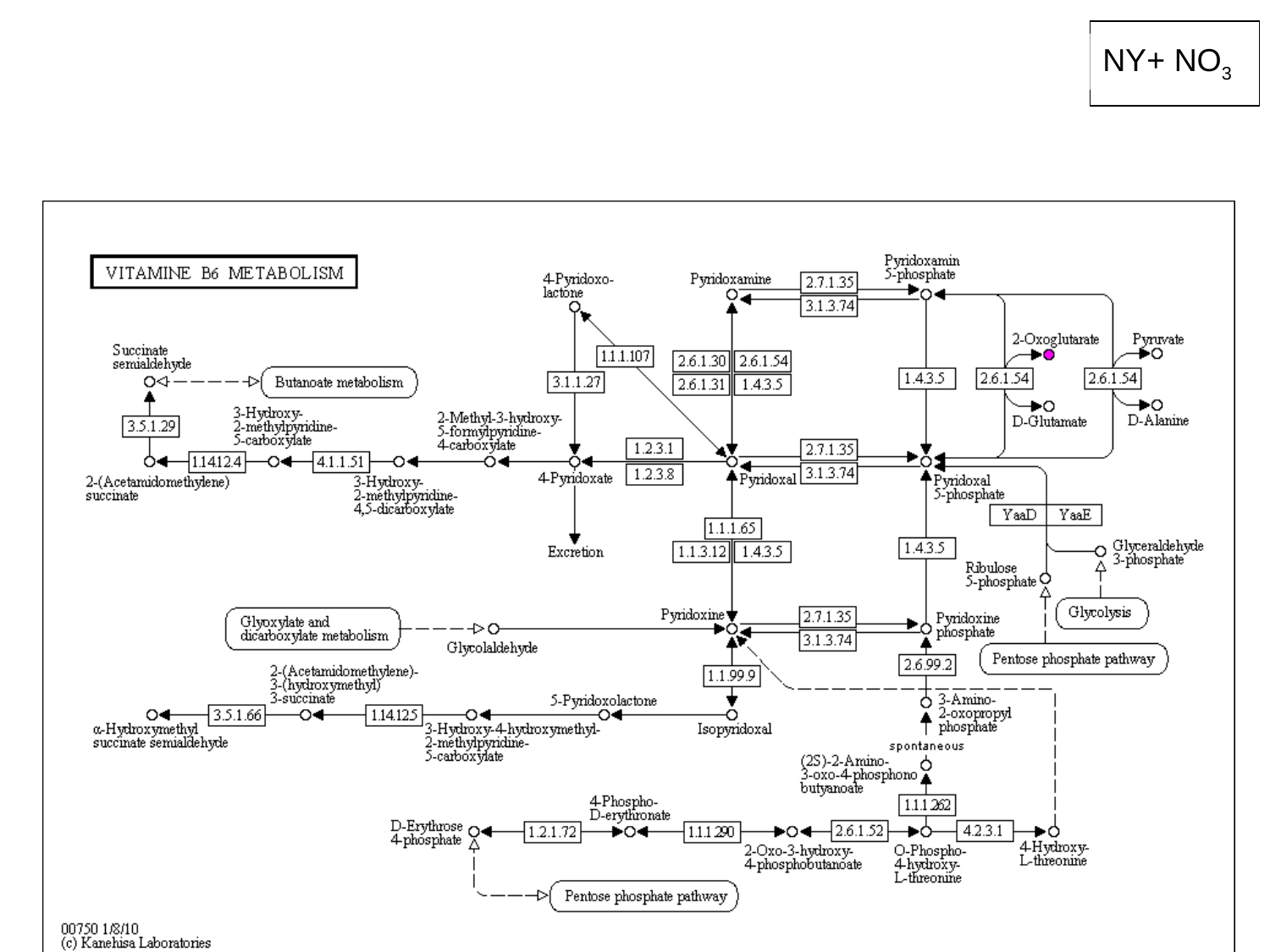

NY+ NO3

## Slide 43
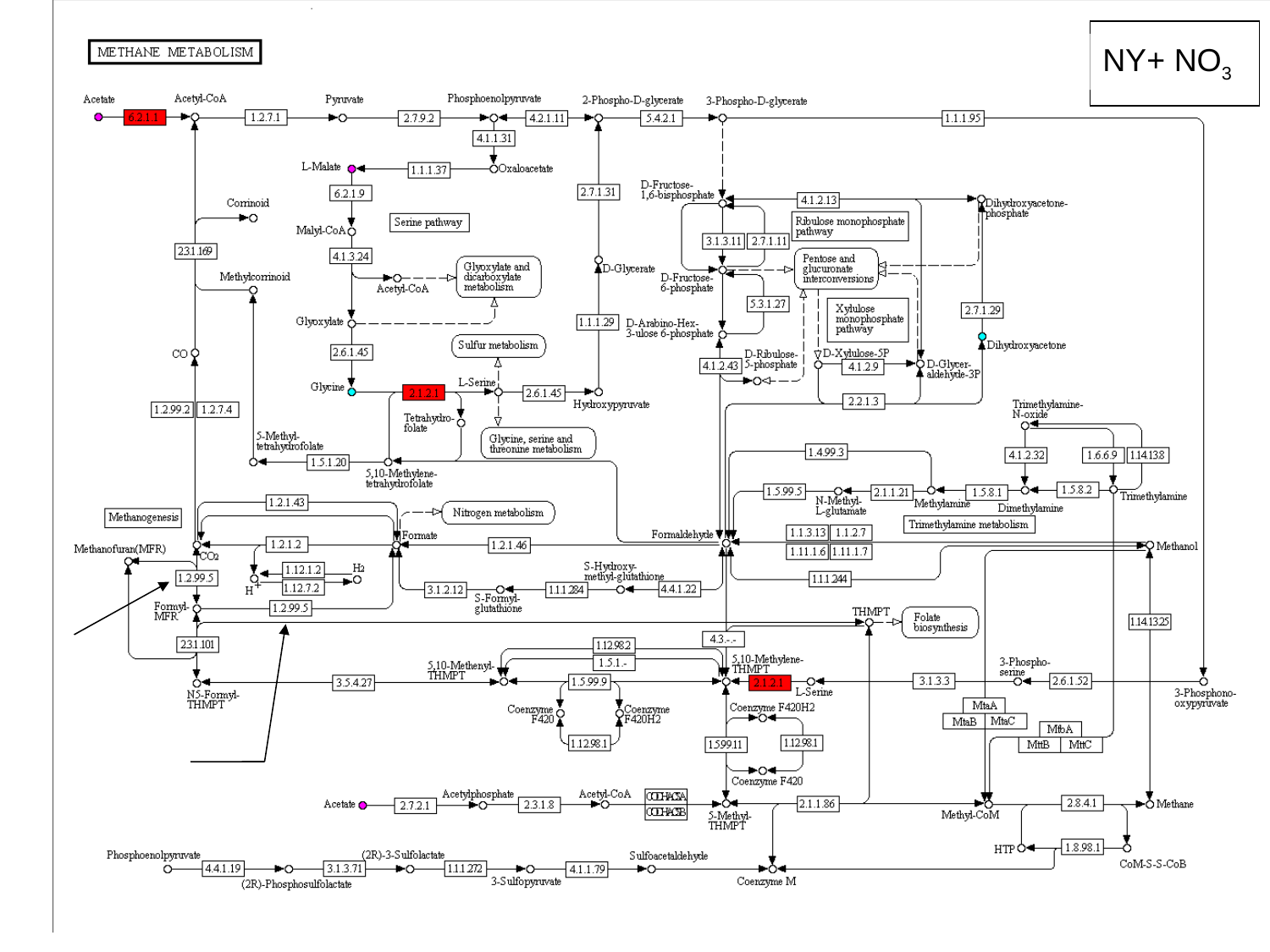

NY+ NO3

## Slide 44
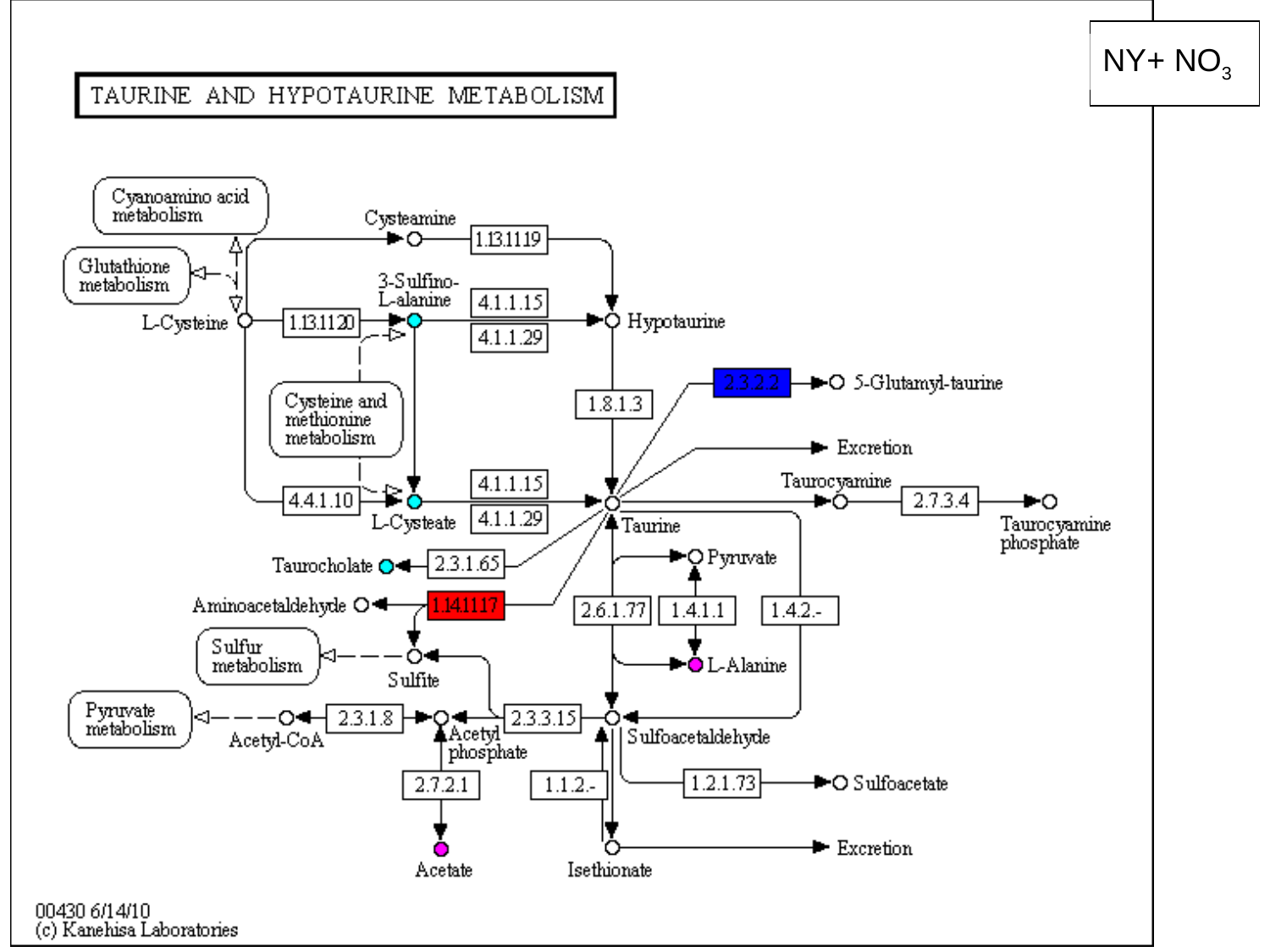

NY+ NO3
